# Supplementary figures and images for: Genetically incorporated crosslinkers reveal NleE attenuates host autophagy dependent on PSMD10 (part 1 of 2)
Source: eLife. 2021 Jul 13;10:e69047. doi: 10.7554/eLife.69047 (PMC8324295; doi:10.7554/eLife.69047)

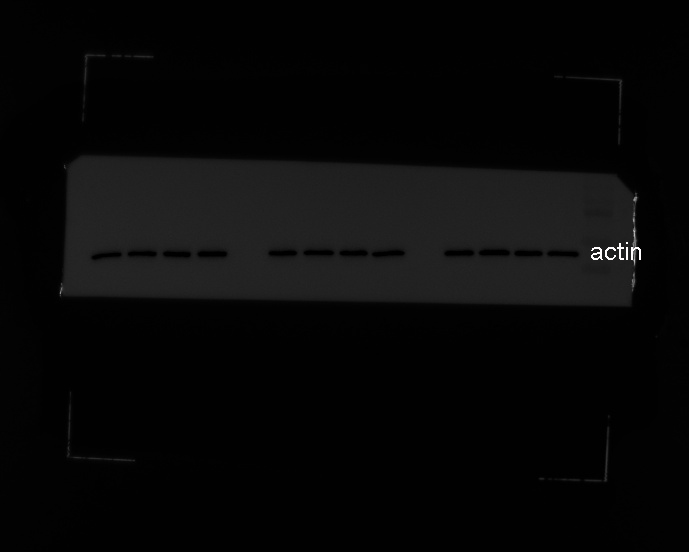

Supplement: Figure 1—source data 9. [file elife-69047-fig1-data9.zip › Figure 1-source data 9. Original western blot files for Figure 1/Figure 1-source data B1.jpg]

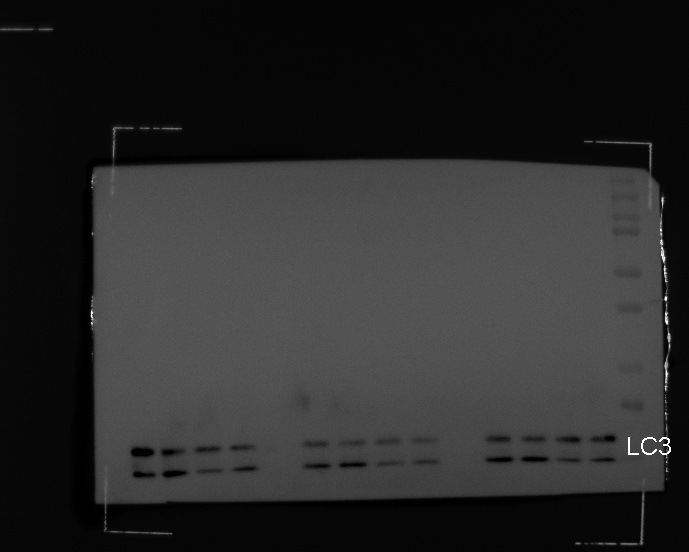

Supplement: Figure 1—source data 9. [file elife-69047-fig1-data9.zip › Figure 1-source data 9. Original western blot files for Figure 1/Figure 1-source data B2.jpg]

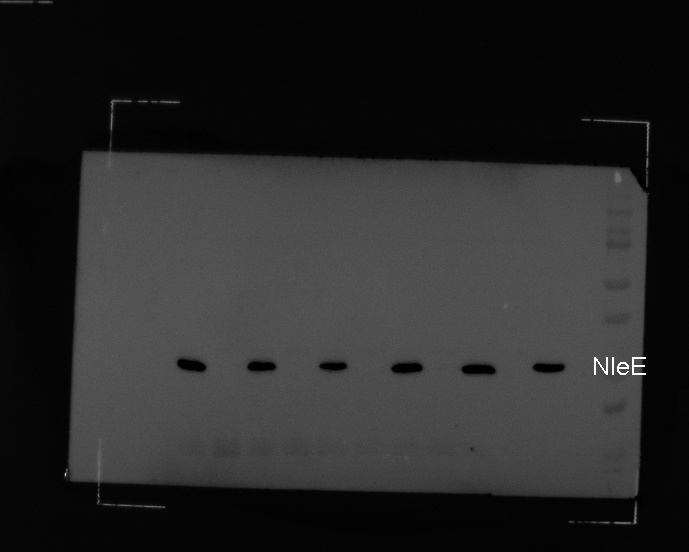

Supplement: Figure 1—source data 9. [file elife-69047-fig1-data9.zip › Figure 1-source data 9. Original western blot files for Figure 1/Figure 1-source data B3.jpg]

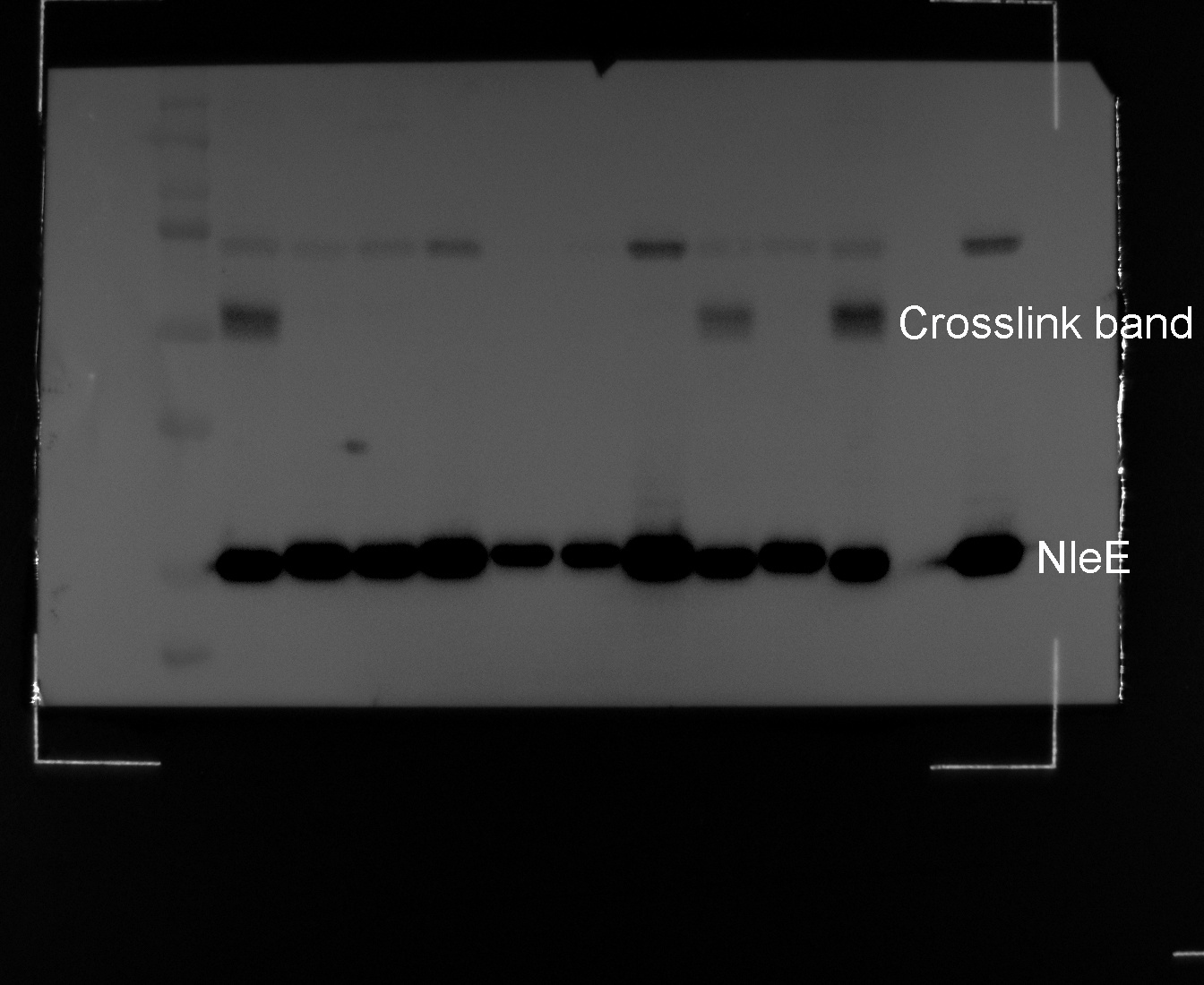

Supplement: Figure 2—source data 1. [file elife-69047-fig2-data1.zip › Figure 2-source data 1. Original western blot files for Figure 2/Figure 2-source data C.jpg]

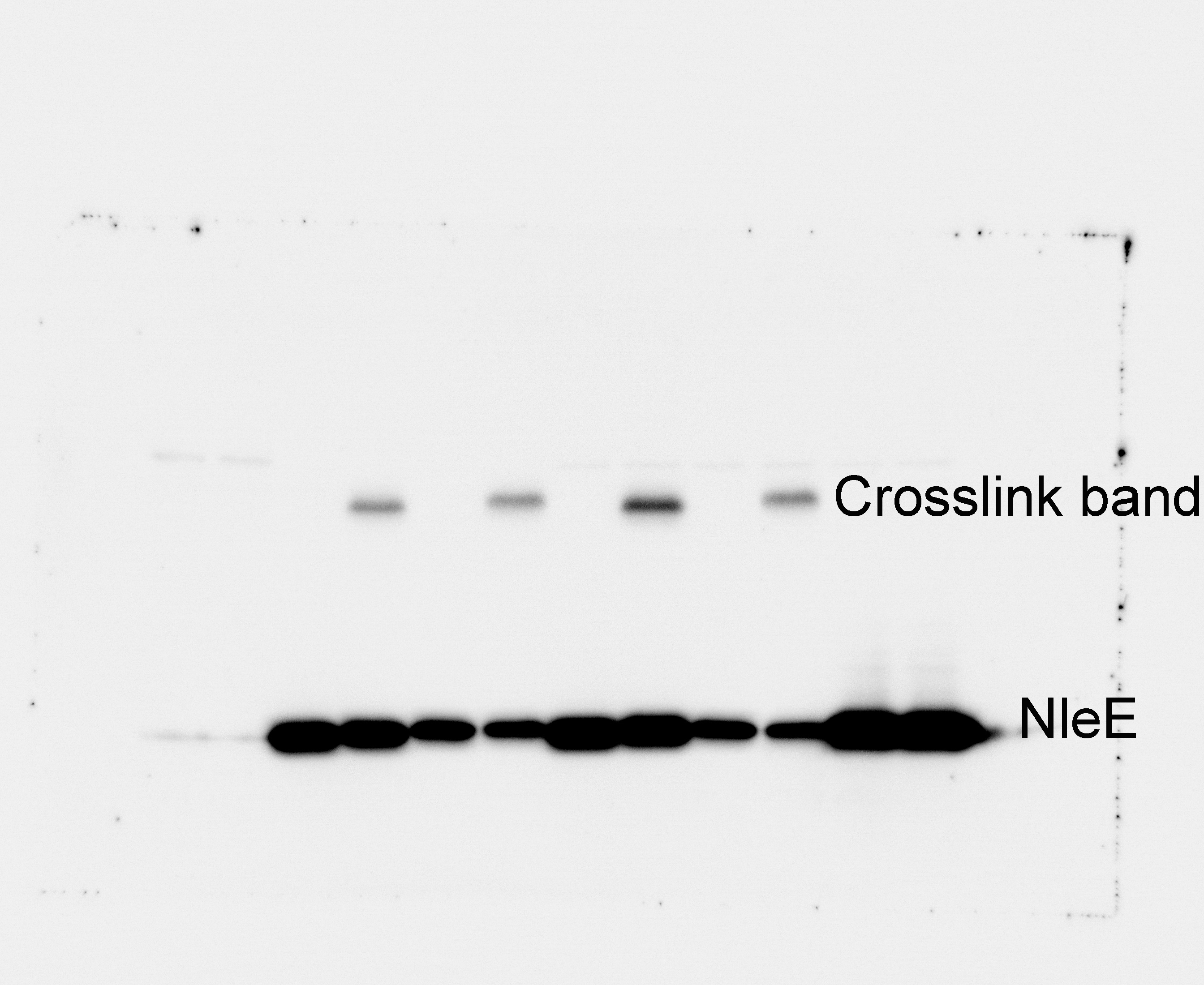

Supplement: Figure 2—source data 1. [file elife-69047-fig2-data1.zip › Figure 2-source data 1. Original western blot files for Figure 2/Figure 2-source data D.jpg]

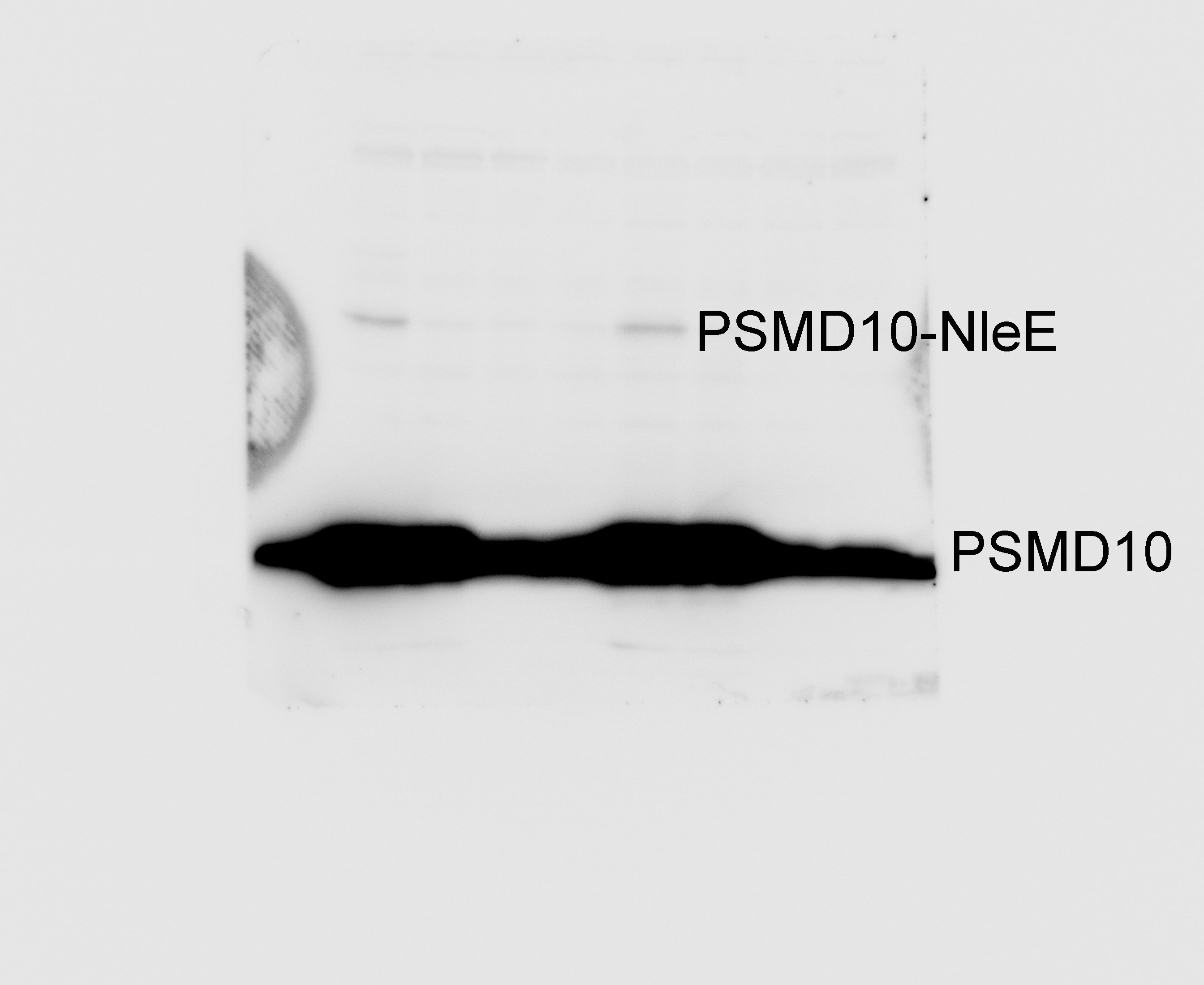

Supplement: Figure 2—source data 1. [file elife-69047-fig2-data1.zip › Figure 2-source data 1. Original western blot files for Figure 2/Figure 2-source data E1.jpg]

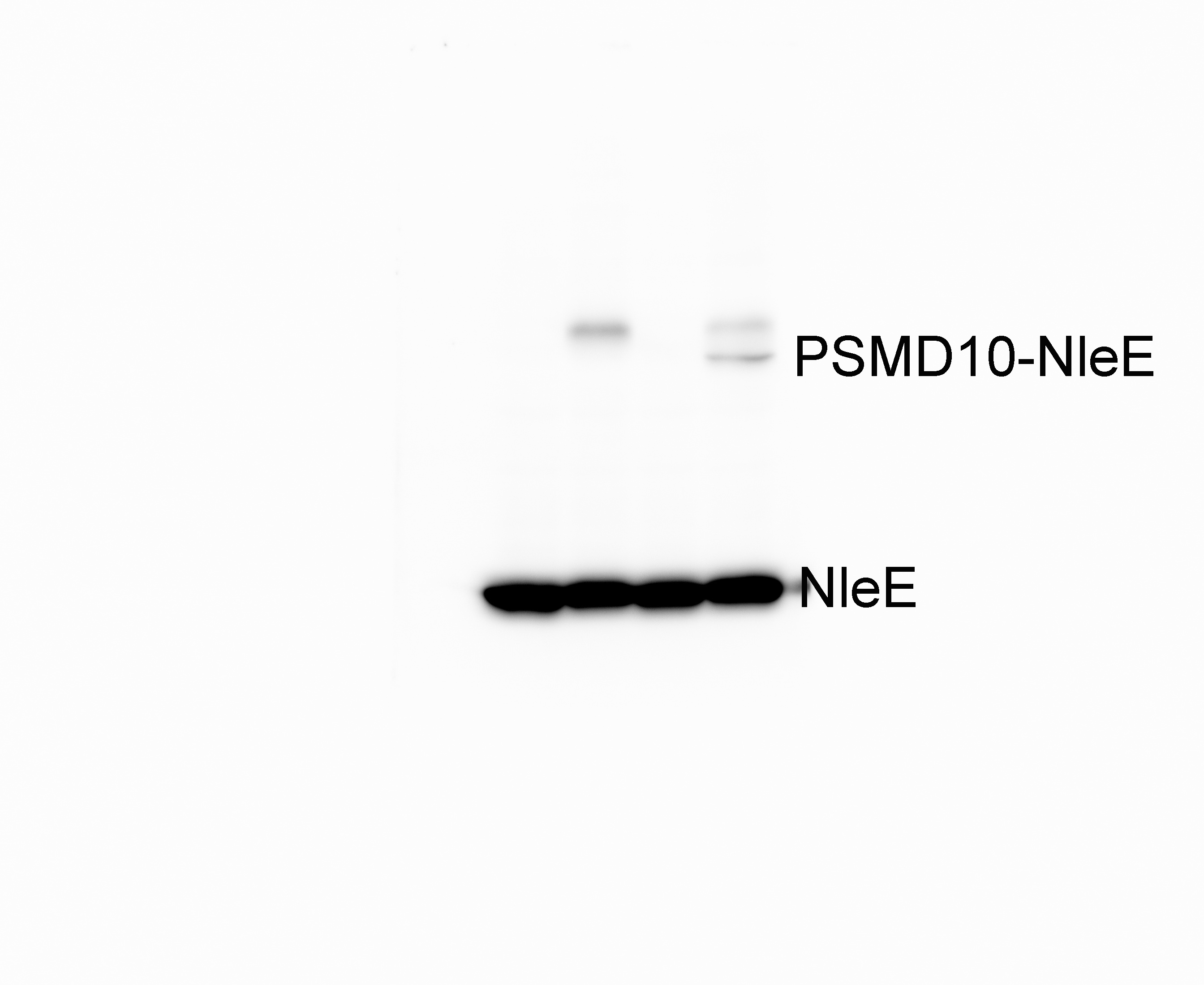

Supplement: Figure 2—source data 1. [file elife-69047-fig2-data1.zip › Figure 2-source data 1. Original western blot files for Figure 2/Figure 2-source data E2.jpg]

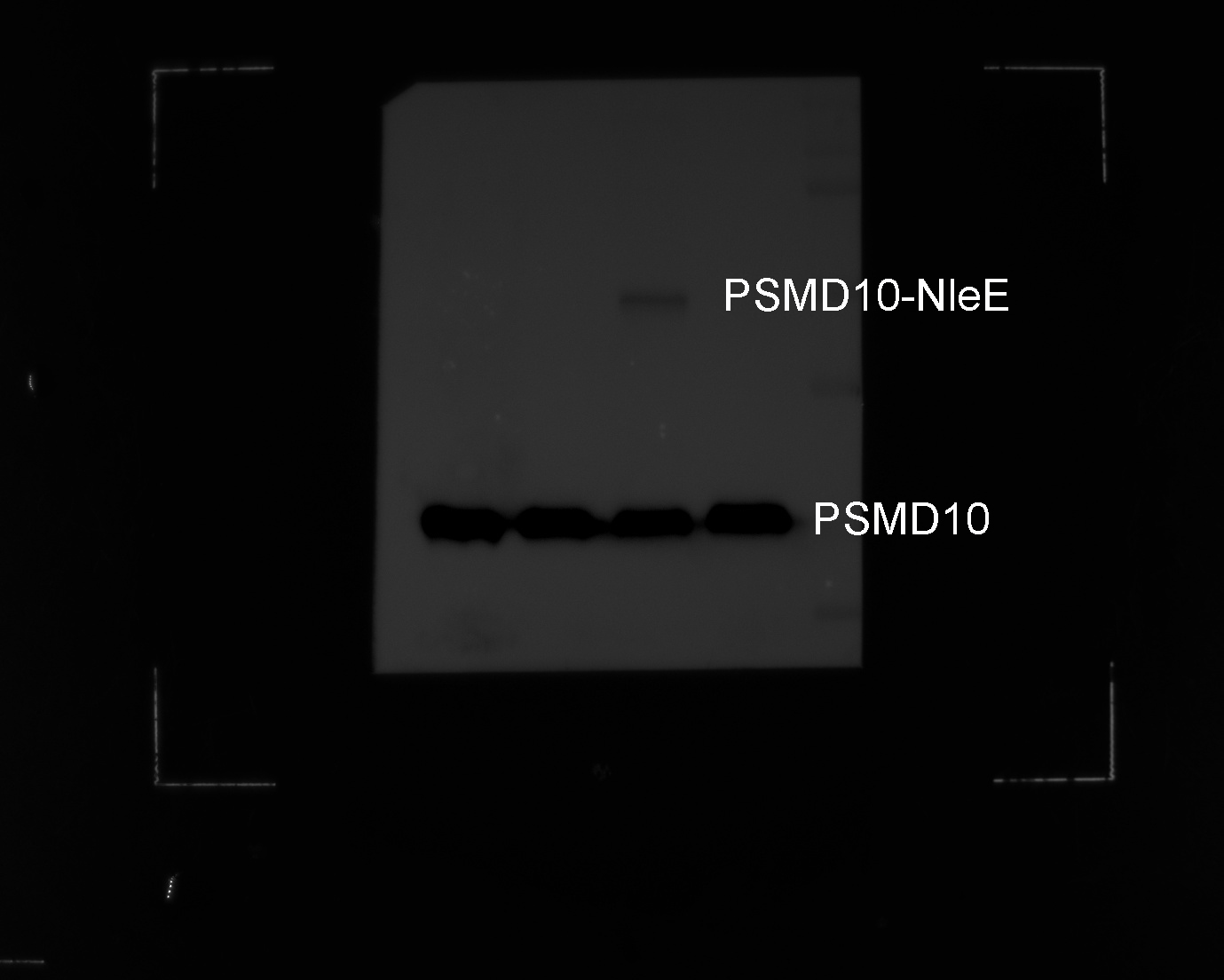

Supplement: Figure 2—source data 1. [file elife-69047-fig2-data1.zip › Figure 2-source data 1. Original western blot files for Figure 2/Figure 2-source data E3.jpg]

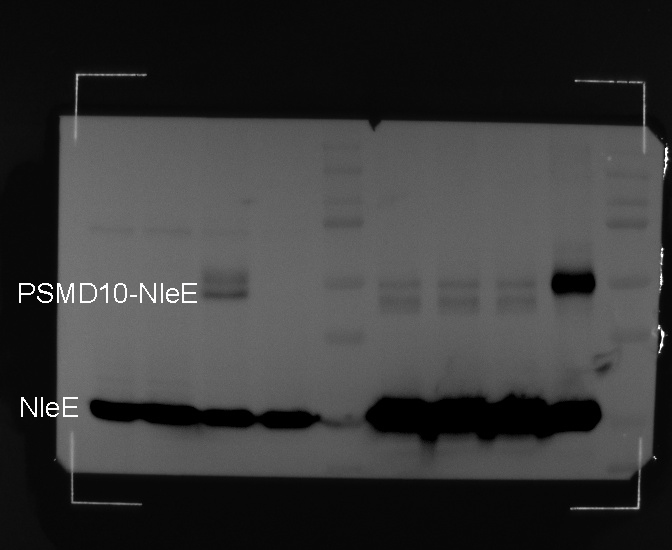

Supplement: Figure 2—source data 1. [file elife-69047-fig2-data1.zip › Figure 2-source data 1. Original western blot files for Figure 2/Figure 2-source data E4.jpg]

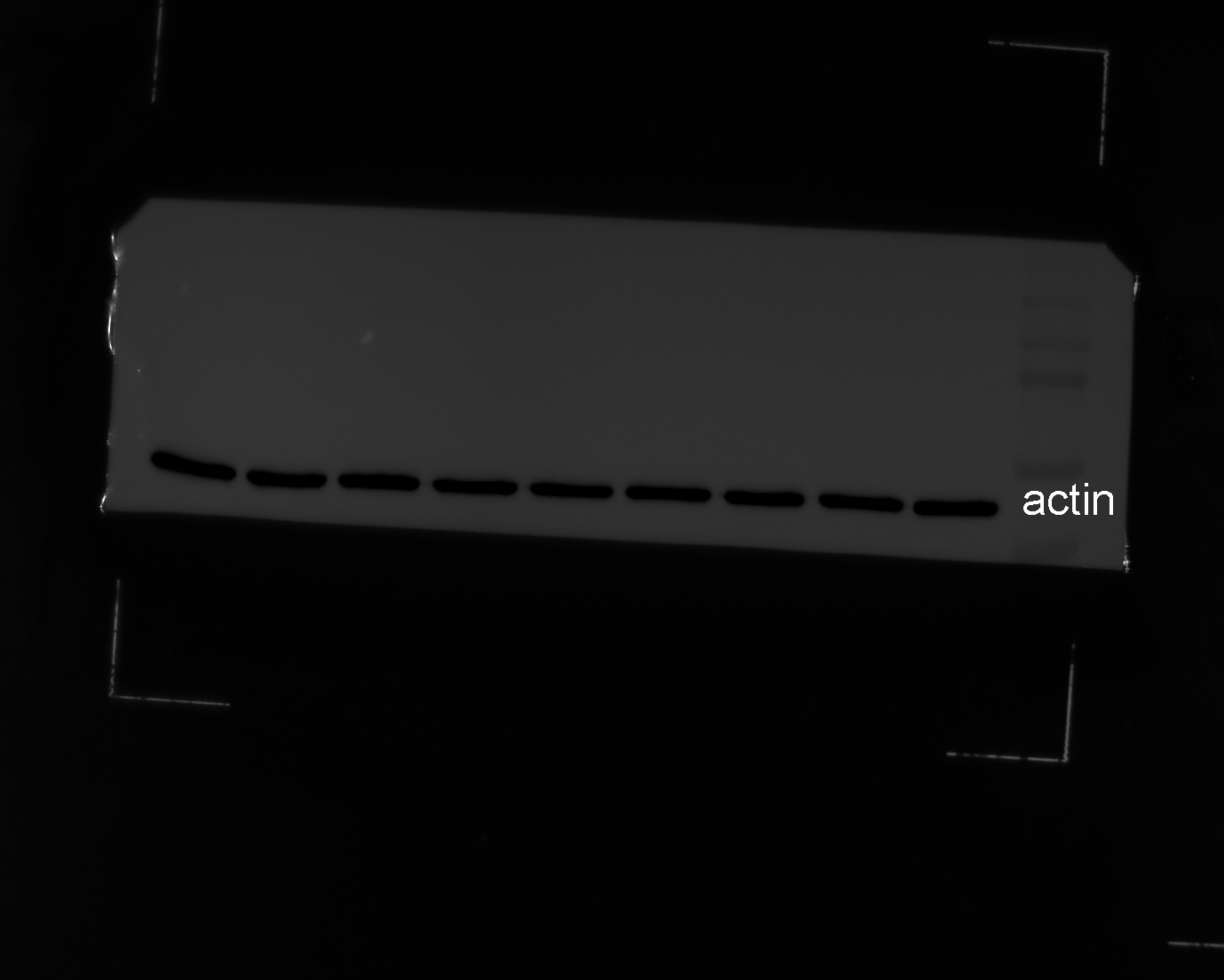

Supplement: Figure 2—figure supplement 1—source data 2. [file elife-69047-fig2-figsupp1-data2.zip › Figure 2-figure supplement 1-source data 2. Original western blot files for Figure 2-figure supplement 1/Figure 2-figure supplement 1-source data A1.jpg]

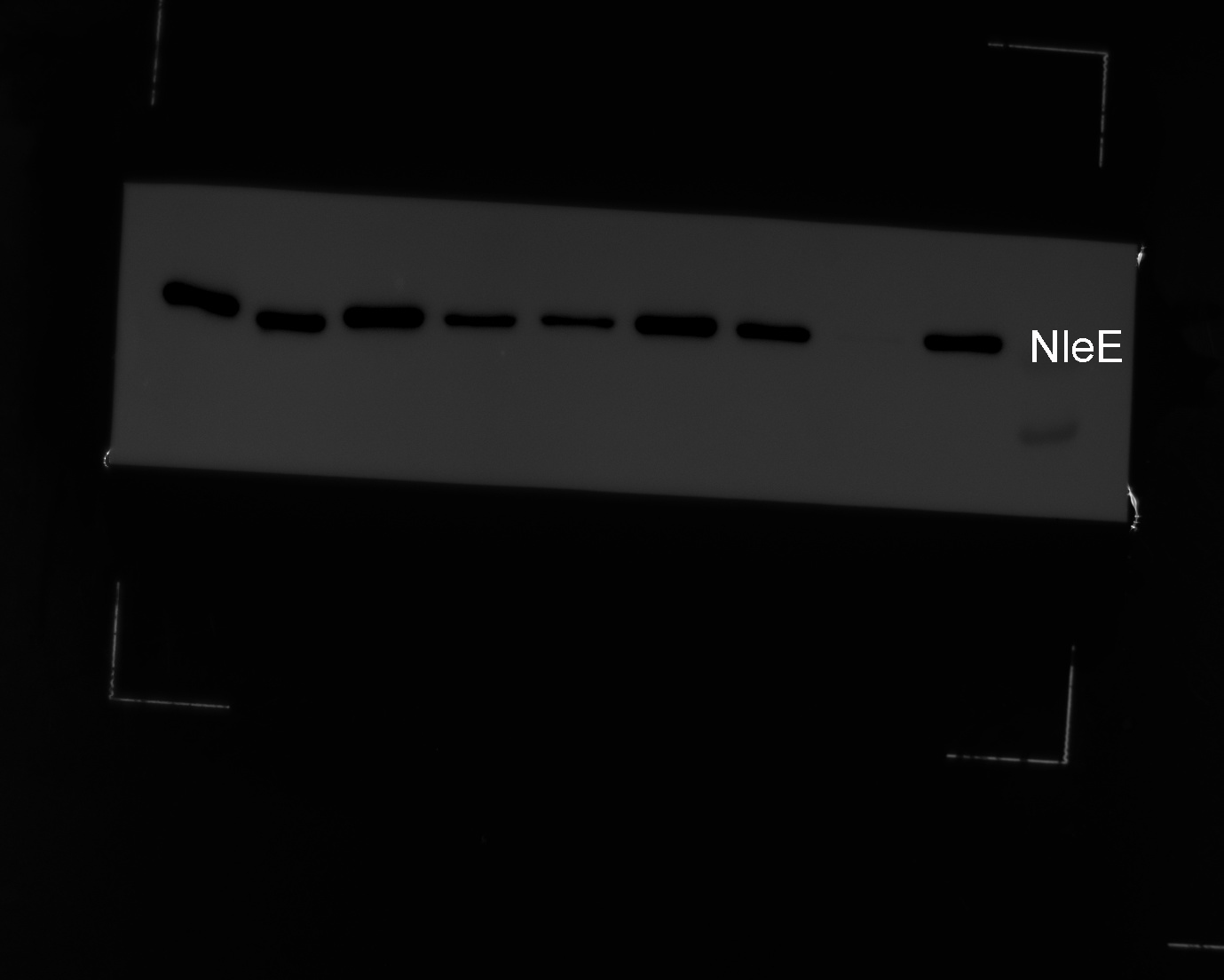

Supplement: Figure 2—figure supplement 1—source data 2. [file elife-69047-fig2-figsupp1-data2.zip › Figure 2-figure supplement 1-source data 2. Original western blot files for Figure 2-figure supplement 1/Figure 2-figure supplement 1-source data A2.jpg]

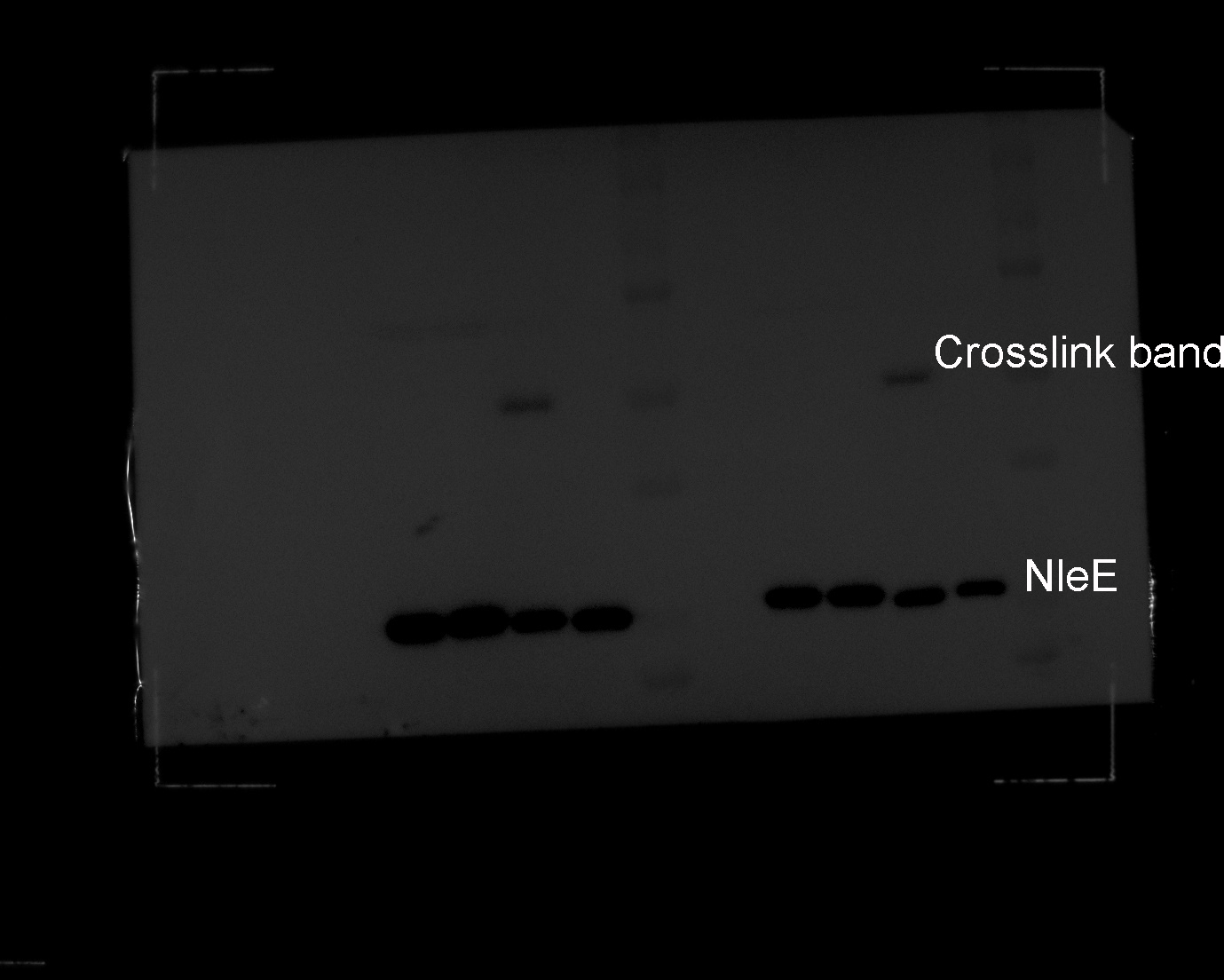

Supplement: Figure 2—figure supplement 1—source data 2. [file elife-69047-fig2-figsupp1-data2.zip › Figure 2-figure supplement 1-source data 2. Original western blot files for Figure 2-figure supplement 1/Figure 2-figure supplement 1-source data C.jpg]

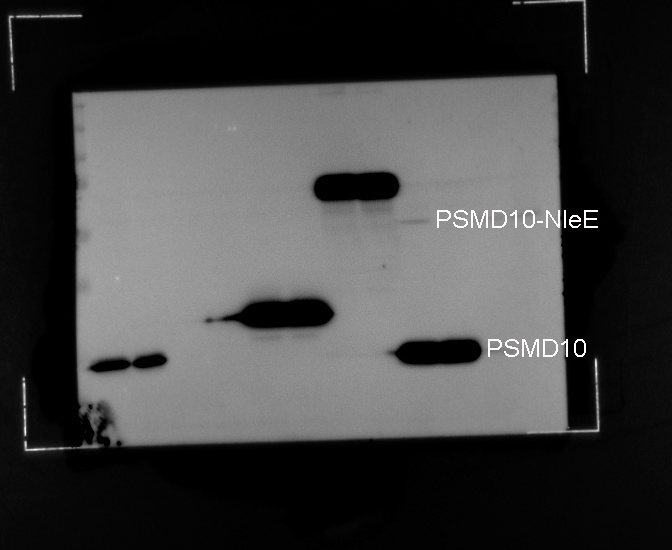

Supplement: Figure 2—figure supplement 2—source data 2. [file elife-69047-fig2-figsupp2-data2.zip › Figure 2-figure supplement 2-source data 2. Original western blot files for Figure 2-figure supplement 2/Figure 2-figure supplement 2-source data C1.jpg]

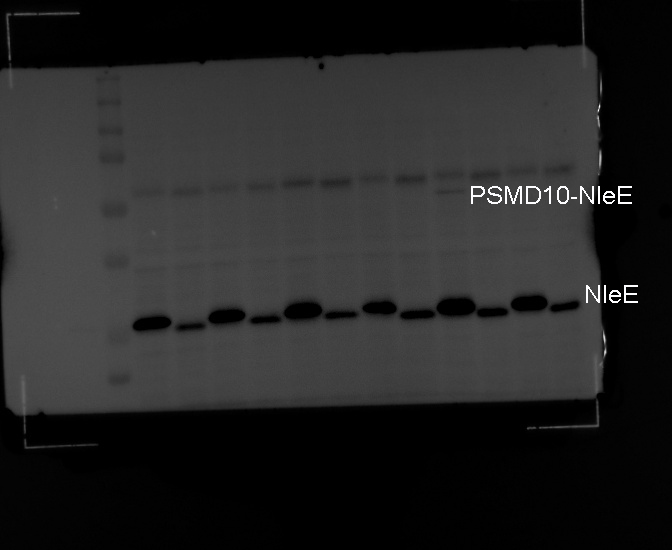

Supplement: Figure 2—figure supplement 2—source data 2. [file elife-69047-fig2-figsupp2-data2.zip › Figure 2-figure supplement 2-source data 2. Original western blot files for Figure 2-figure supplement 2/Figure 2-figure supplement 2-source data C2.jpg]

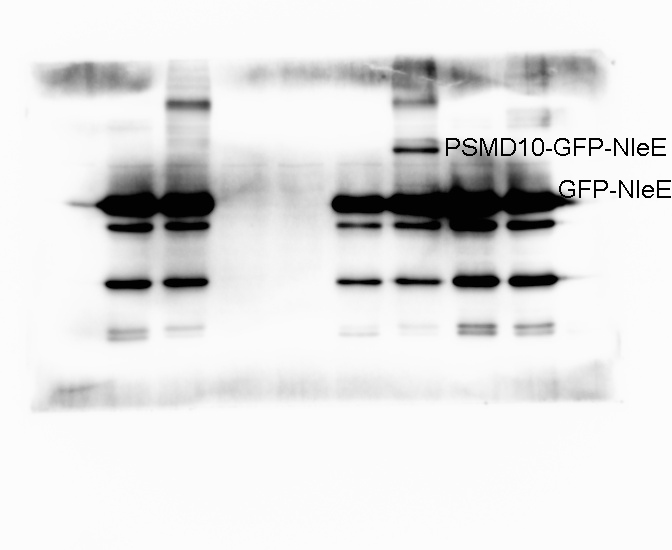

Supplement: Figure 2—figure supplement 2—source data 2. [file elife-69047-fig2-figsupp2-data2.zip › Figure 2-figure supplement 2-source data 2. Original western blot files for Figure 2-figure supplement 2/Figure 2-figure supplement 2-source data D1.jpg]

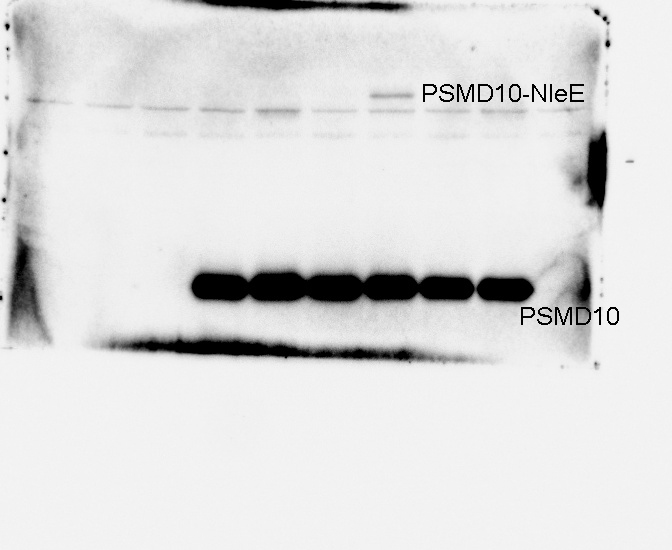

Supplement: Figure 2—figure supplement 2—source data 2. [file elife-69047-fig2-figsupp2-data2.zip › Figure 2-figure supplement 2-source data 2. Original western blot files for Figure 2-figure supplement 2/Figure 2-figure supplement 2-source data D2.jpg]

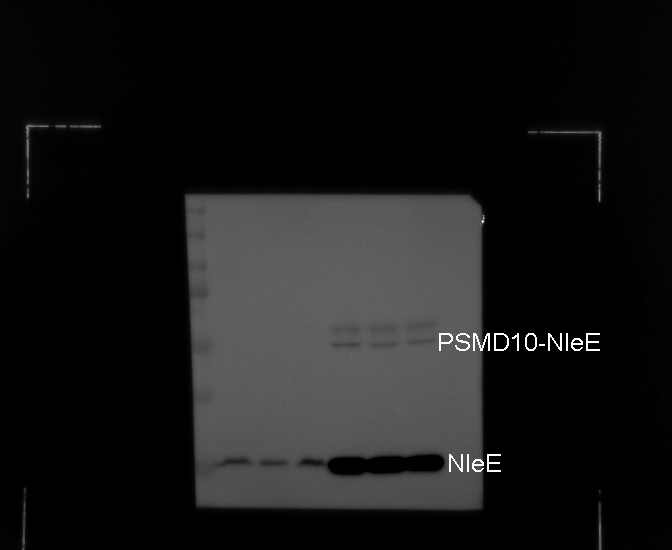

Supplement: Figure 2—figure supplement 2—source data 2. [file elife-69047-fig2-figsupp2-data2.zip › Figure 2-figure supplement 2-source data 2. Original western blot files for Figure 2-figure supplement 2/Figure 2-figure supplement 2-source data E.jpg]

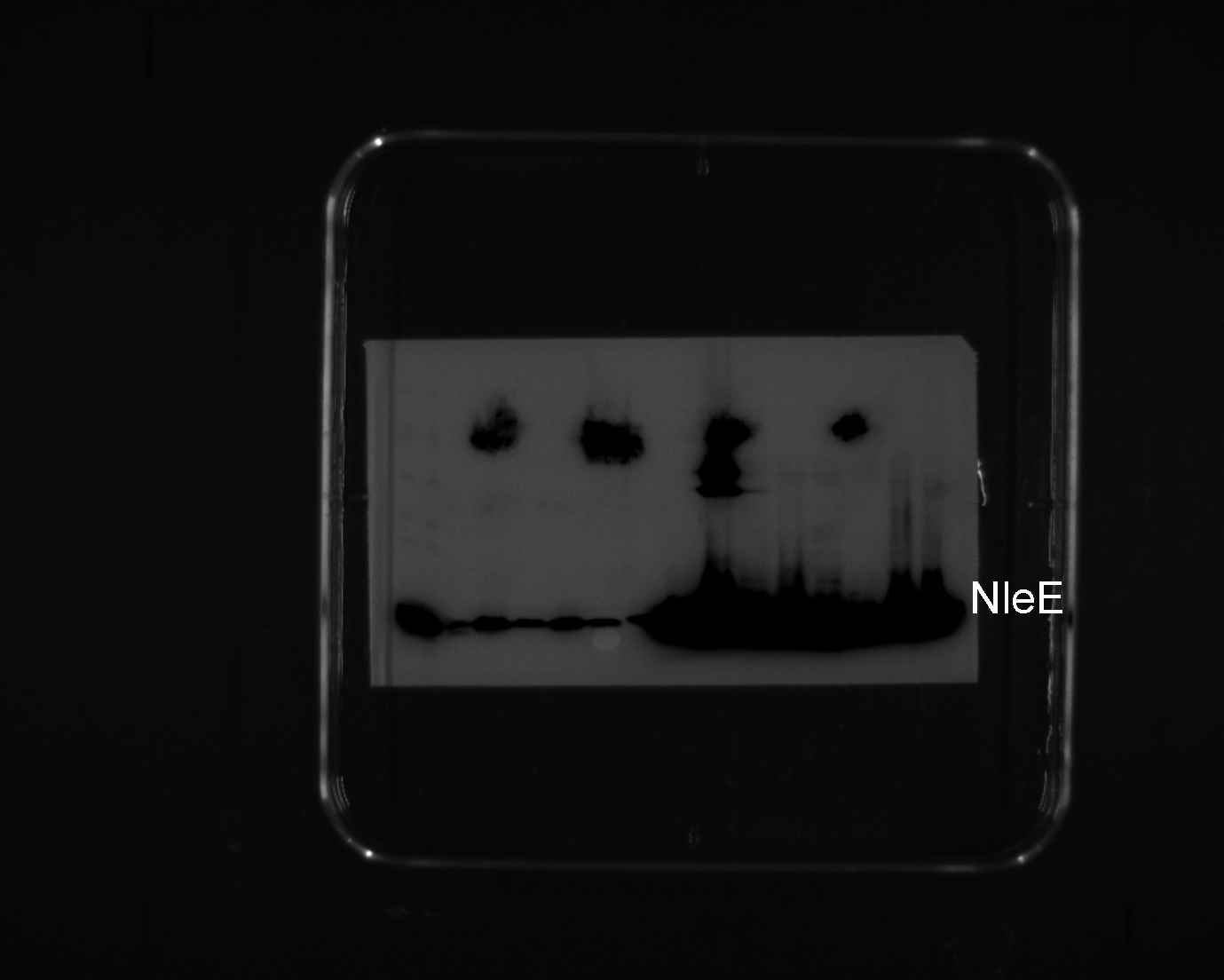

Supplement: Figure 2—figure supplement 2—source data 2. [file elife-69047-fig2-figsupp2-data2.zip › Figure 2-figure supplement 2-source data 2. Original western blot files for Figure 2-figure supplement 2/Figure 2-figure supplement 2-source data F1.jpg]

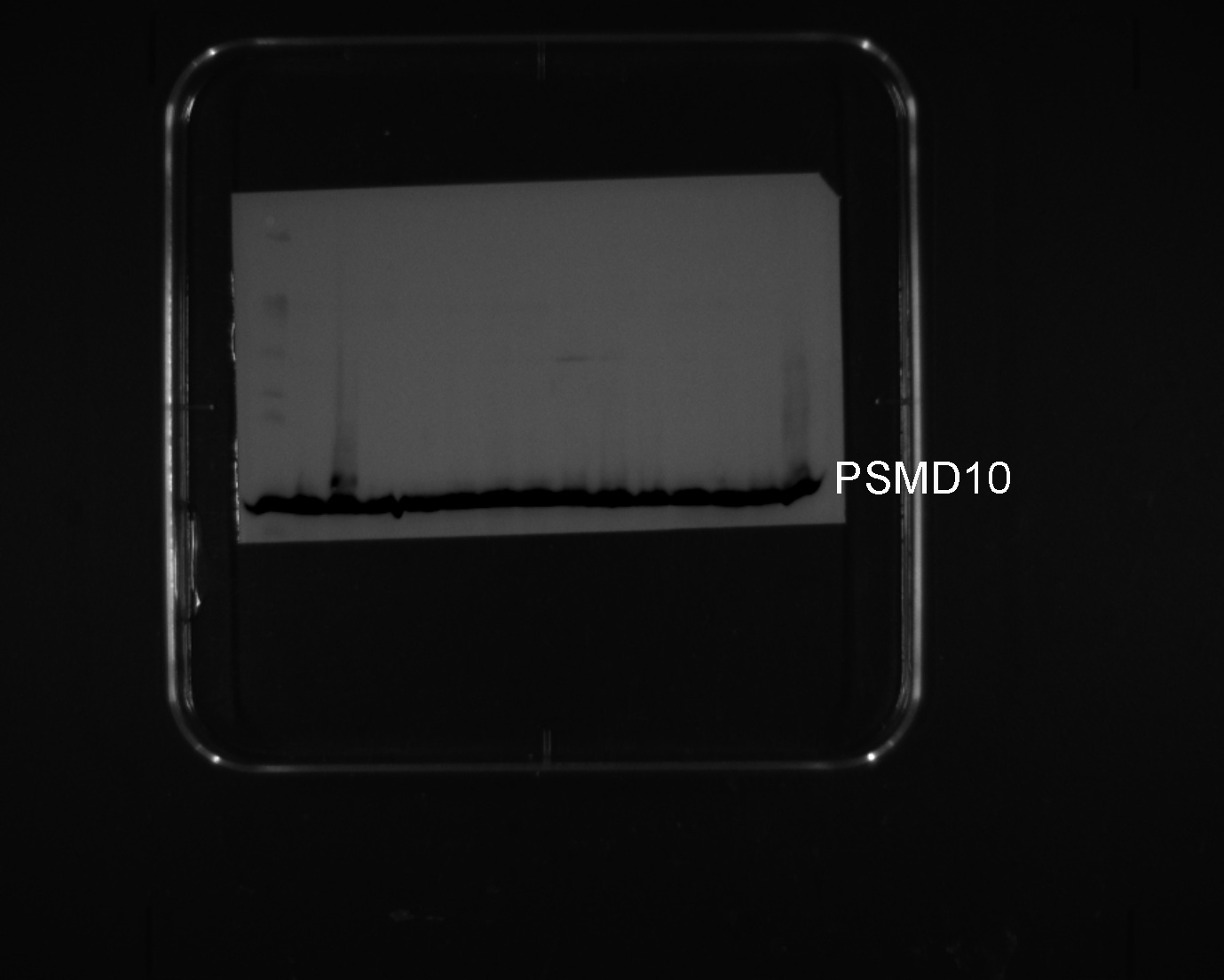

Supplement: Figure 2—figure supplement 2—source data 2. [file elife-69047-fig2-figsupp2-data2.zip › Figure 2-figure supplement 2-source data 2. Original western blot files for Figure 2-figure supplement 2/Figure 2-figure supplement 2-source data F2.jpg]

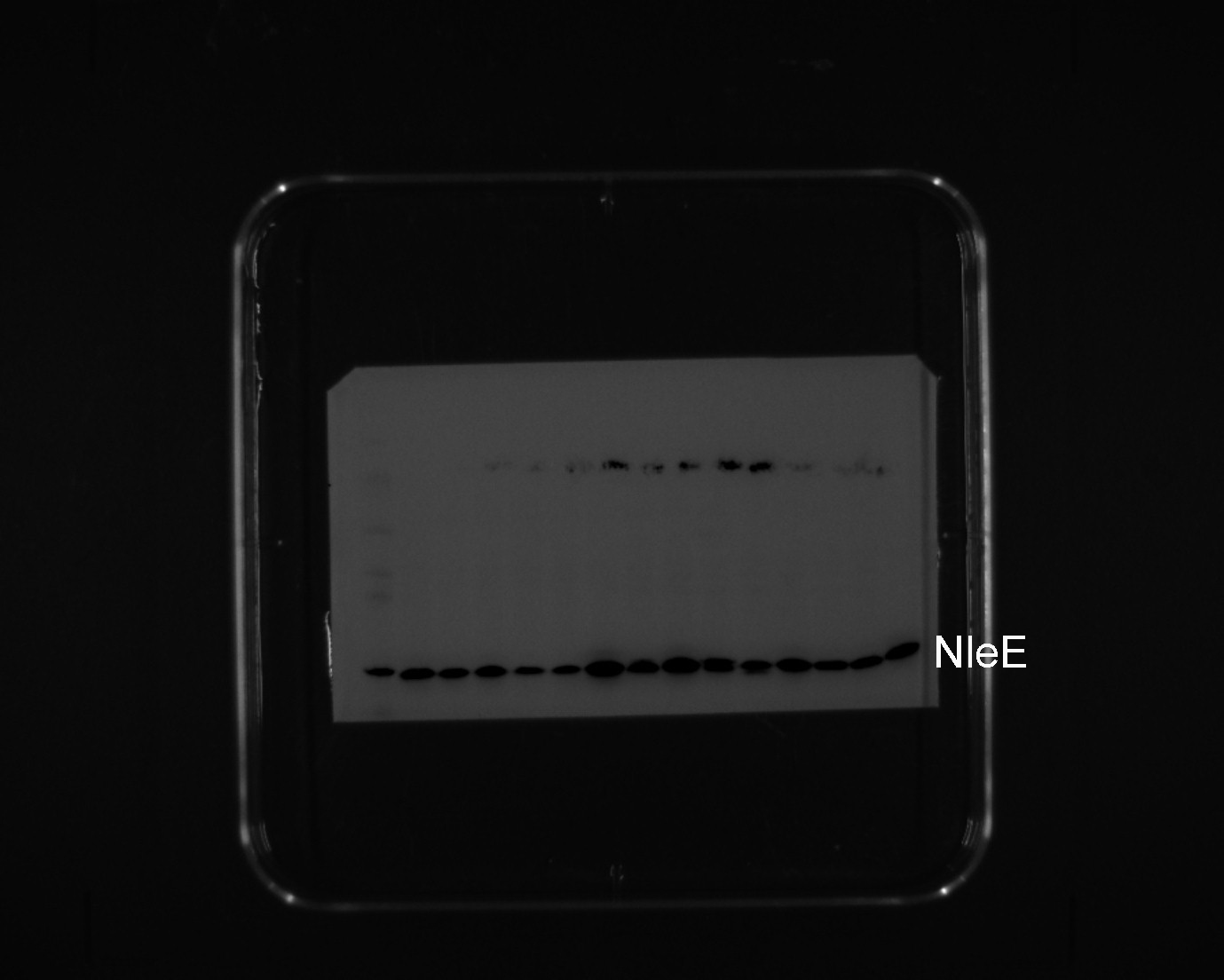

Supplement: Figure 2—figure supplement 2—source data 2. [file elife-69047-fig2-figsupp2-data2.zip › Figure 2-figure supplement 2-source data 2. Original western blot files for Figure 2-figure supplement 2/Figure 2-figure supplement 2-source data G1.jpg]

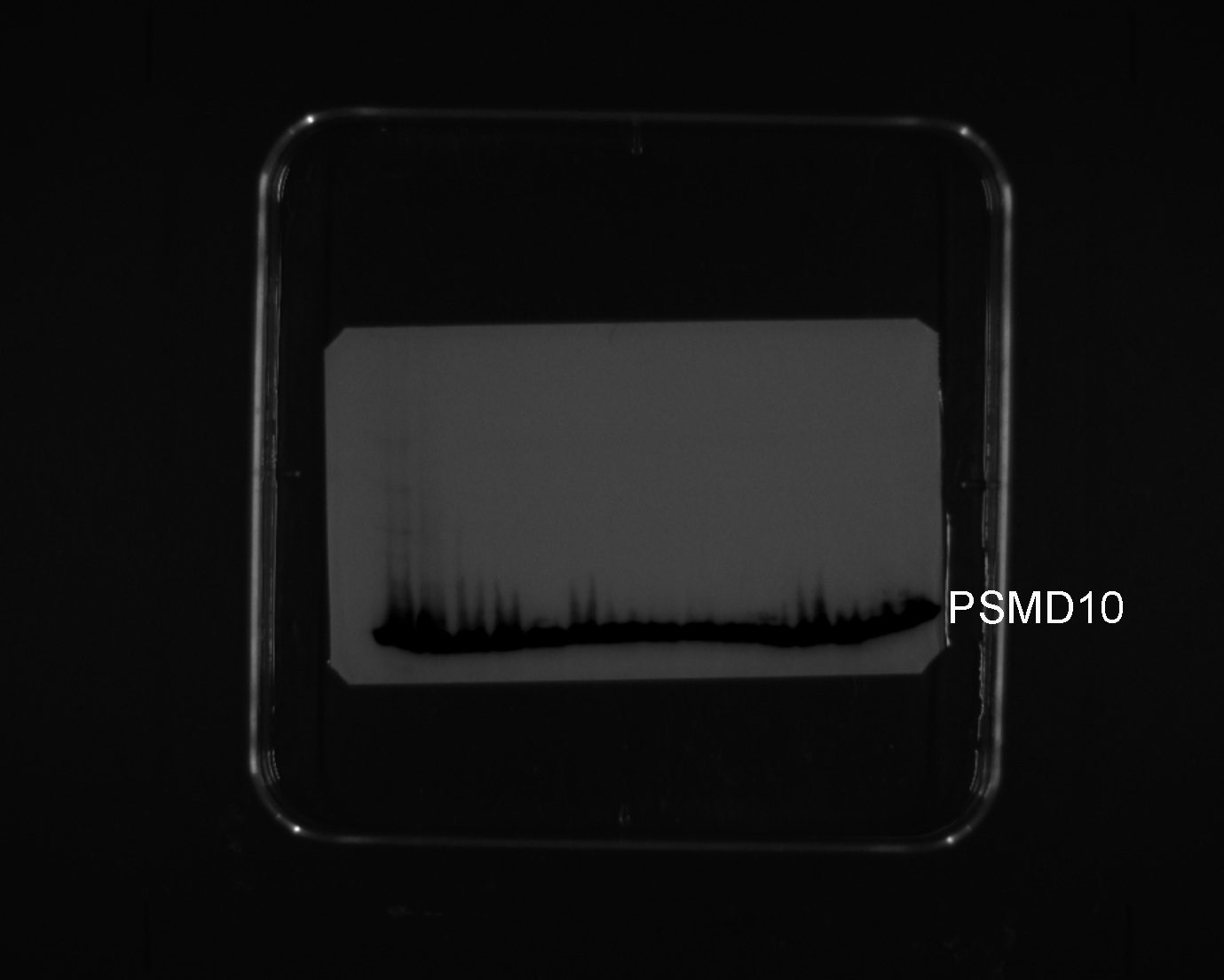

Supplement: Figure 2—figure supplement 2—source data 2. [file elife-69047-fig2-figsupp2-data2.zip › Figure 2-figure supplement 2-source data 2. Original western blot files for Figure 2-figure supplement 2/Figure 2-figure supplement 2-source data G2.jpg]

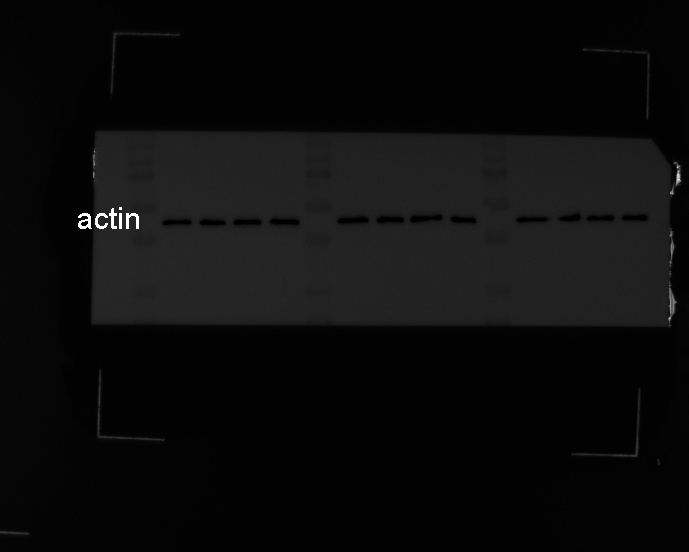

Supplement: Figure 3—source data 6. [file elife-69047-fig3-data6.zip › Figure 3-source data 6. Original western blot files for Figure 3/Figure 3-source data E1.jpg]

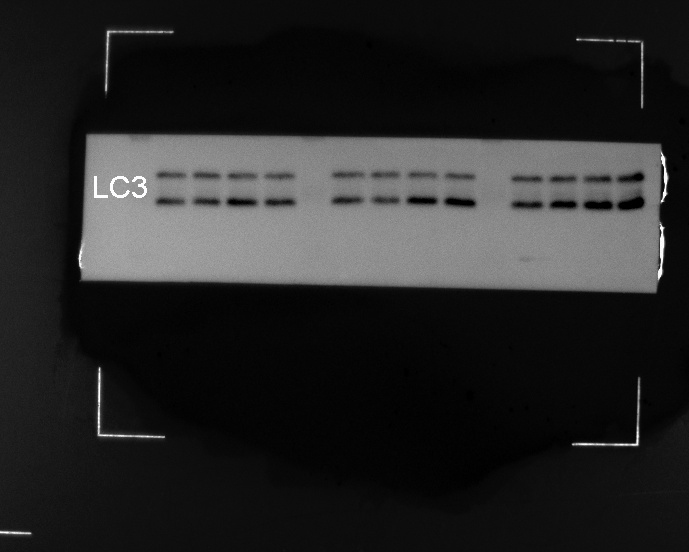

Supplement: Figure 3—source data 6. [file elife-69047-fig3-data6.zip › Figure 3-source data 6. Original western blot files for Figure 3/Figure 3-source data E2.jpg]

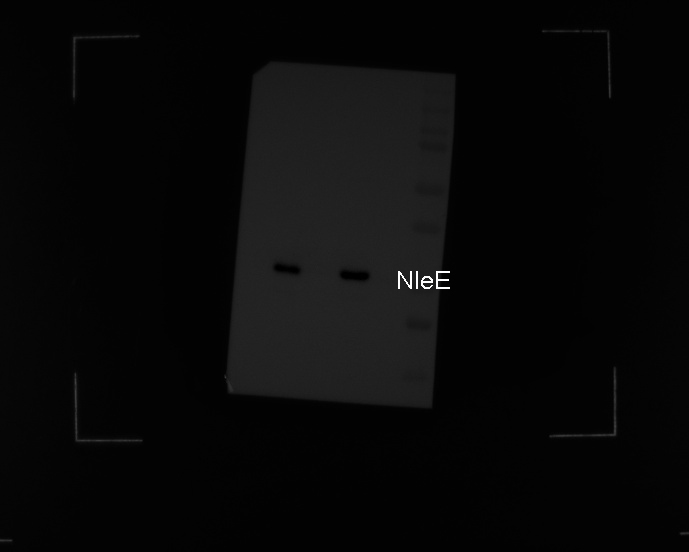

Supplement: Figure 3—source data 6. [file elife-69047-fig3-data6.zip › Figure 3-source data 6. Original western blot files for Figure 3/Figure 3-source data E3.jpg]

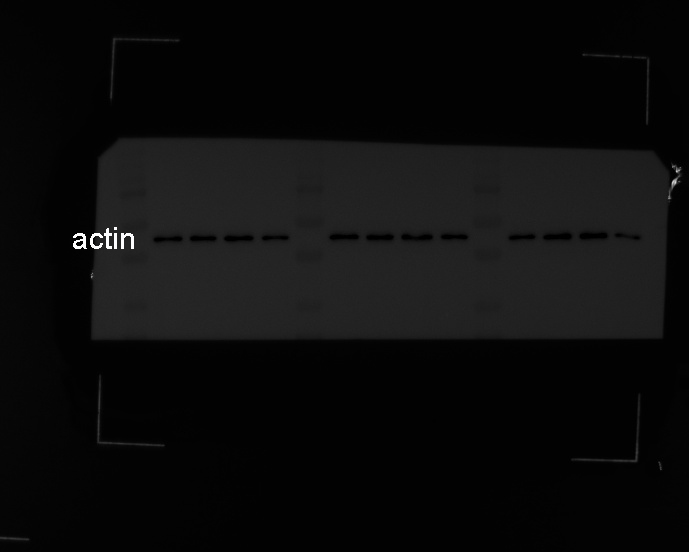

Supplement: Figure 3—source data 6. [file elife-69047-fig3-data6.zip › Figure 3-source data 6. Original western blot files for Figure 3/Figure 3-source data F1.jpg]

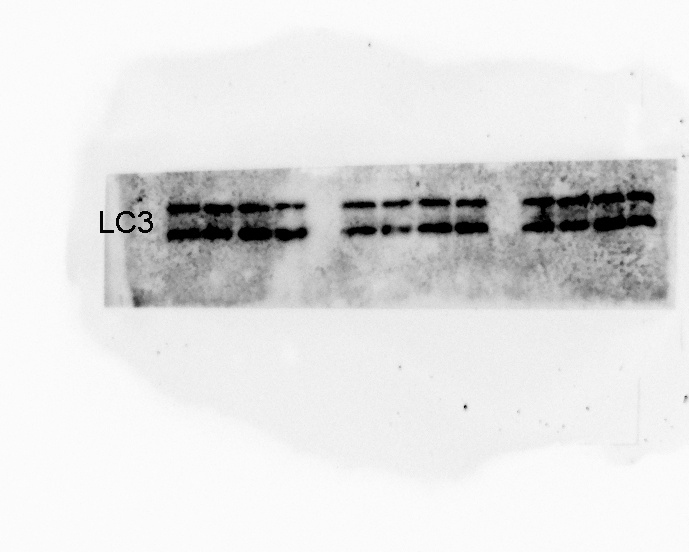

Supplement: Figure 3—source data 6. [file elife-69047-fig3-data6.zip › Figure 3-source data 6. Original western blot files for Figure 3/Figure 3-source data F2.jpg]

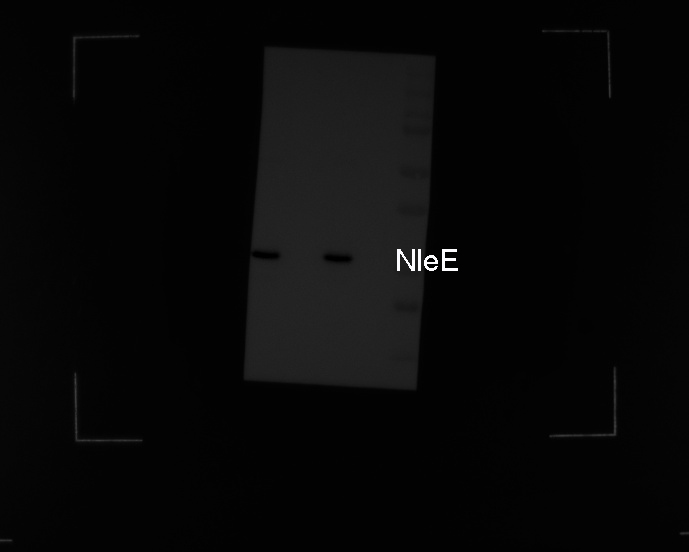

Supplement: Figure 3—source data 6. [file elife-69047-fig3-data6.zip › Figure 3-source data 6. Original western blot files for Figure 3/Figure 3-source data F3.jpg]

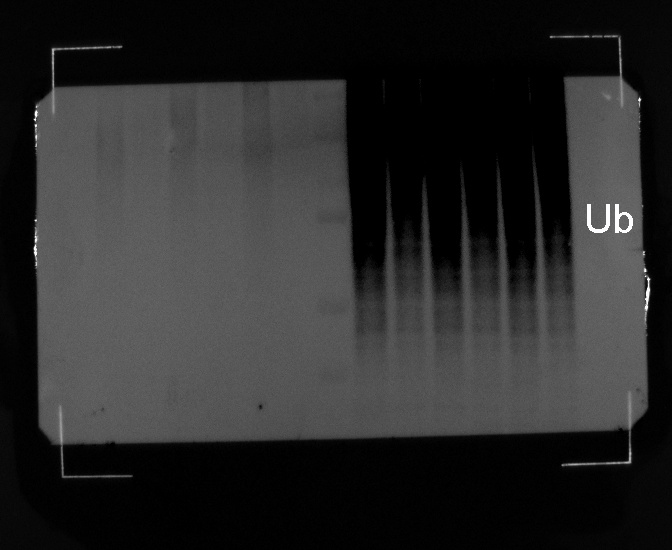

Supplement: Figure 3—figure supplement 1—source data 2. [file elife-69047-fig3-figsupp1-data2.zip › Figure 3-figure supplement 1-source data 2. Original western blot files for Figure 3-figure supplement 1/Figure 3-figure supplement 1-source data A1.jpg]

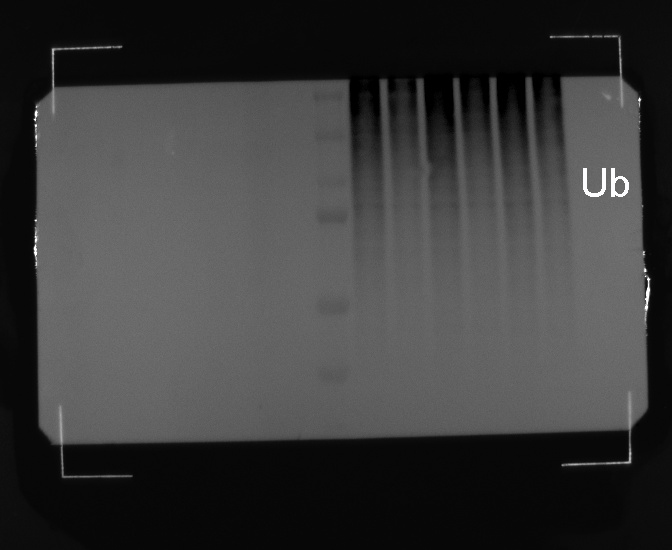

Supplement: Figure 3—figure supplement 1—source data 2. [file elife-69047-fig3-figsupp1-data2.zip › Figure 3-figure supplement 1-source data 2. Original western blot files for Figure 3-figure supplement 1/Figure 3-figure supplement 1-source data A2.jpg]

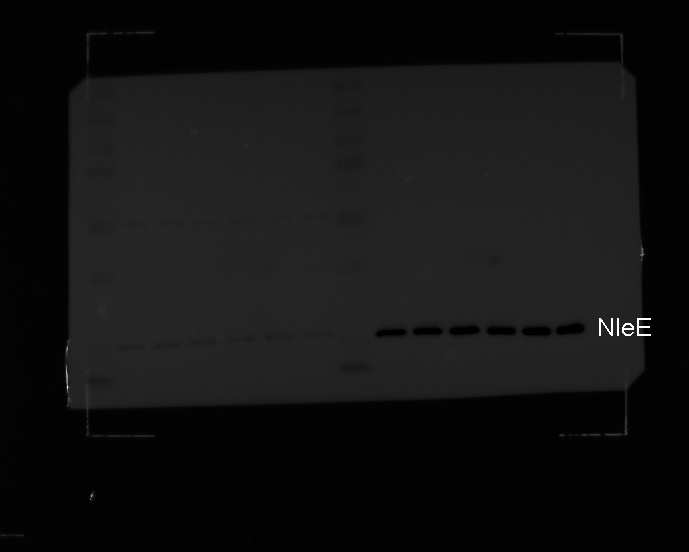

Supplement: Figure 3—figure supplement 1—source data 2. [file elife-69047-fig3-figsupp1-data2.zip › Figure 3-figure supplement 1-source data 2. Original western blot files for Figure 3-figure supplement 1/Figure 3-figure supplement 1-source data A3.jpg]

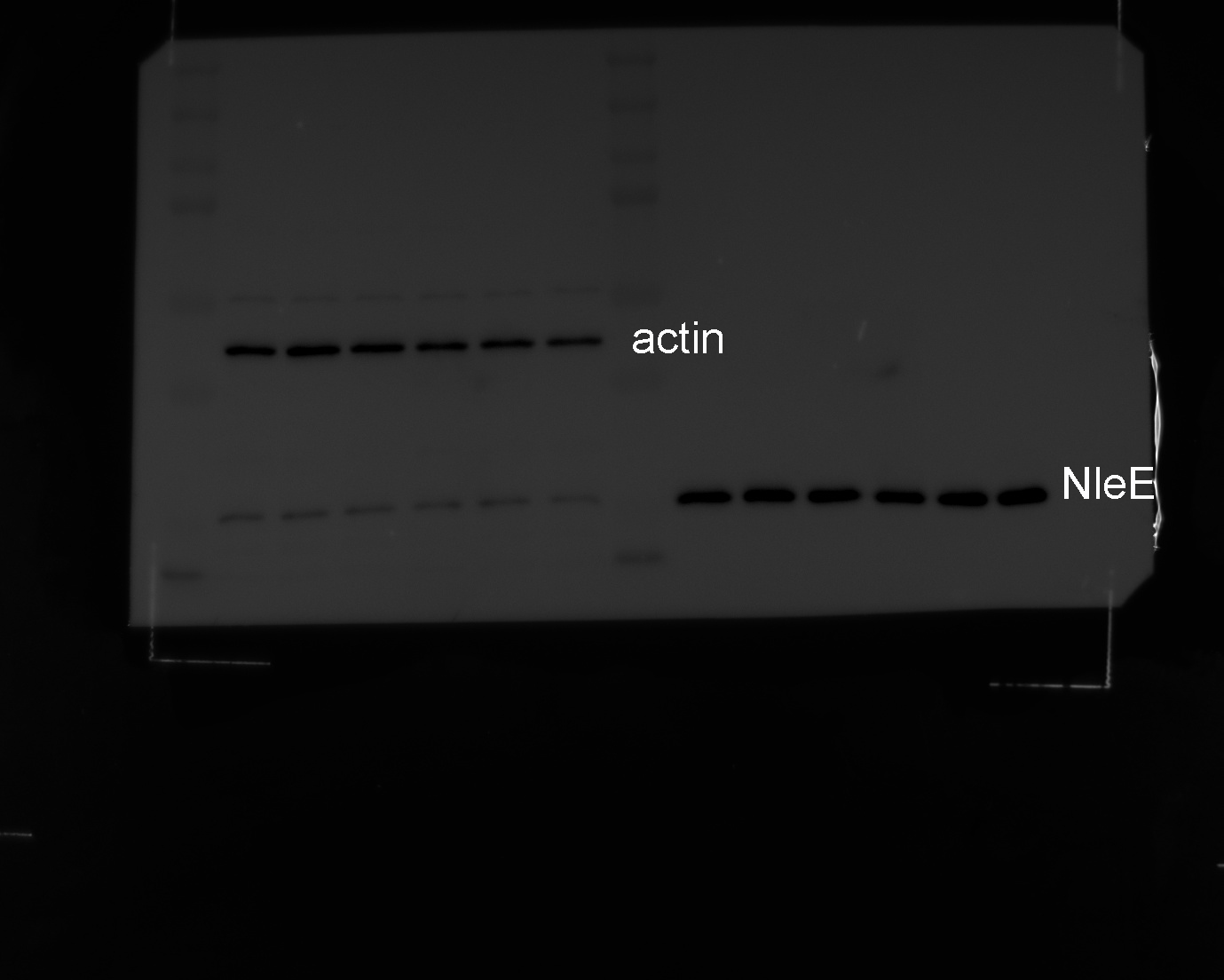

Supplement: Figure 3—figure supplement 1—source data 2. [file elife-69047-fig3-figsupp1-data2.zip › Figure 3-figure supplement 1-source data 2. Original western blot files for Figure 3-figure supplement 1/Figure 3-figure supplement 1-source data A4.jpg]

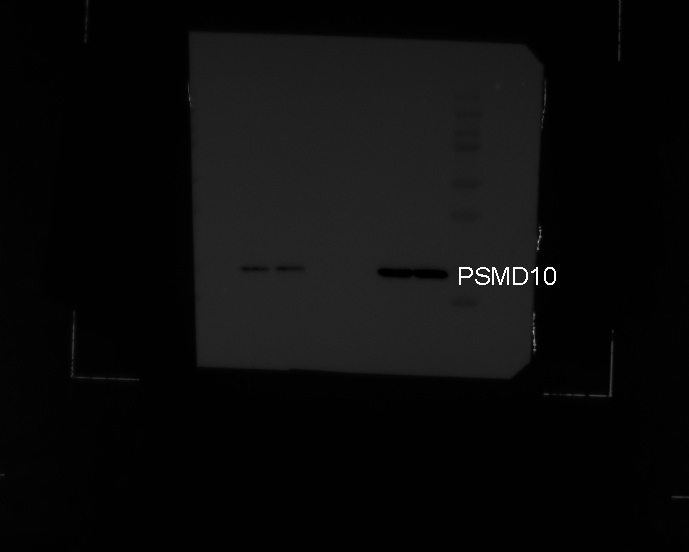

Supplement: Figure 3—figure supplement 1—source data 2. [file elife-69047-fig3-figsupp1-data2.zip › Figure 3-figure supplement 1-source data 2. Original western blot files for Figure 3-figure supplement 1/Figure 3-figure supplement 1-source data A5.jpg]

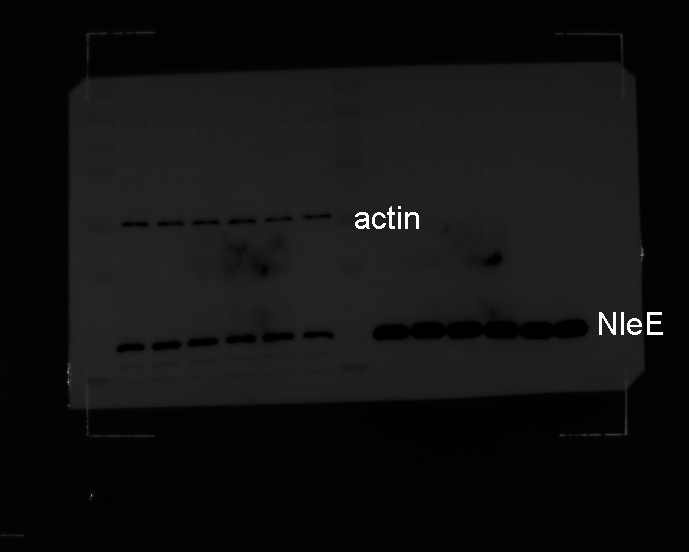

Supplement: Figure 3—figure supplement 1—source data 2. [file elife-69047-fig3-figsupp1-data2.zip › Figure 3-figure supplement 1-source data 2. Original western blot files for Figure 3-figure supplement 1/Figure 3-figure supplement 1-source data A6.jpg]

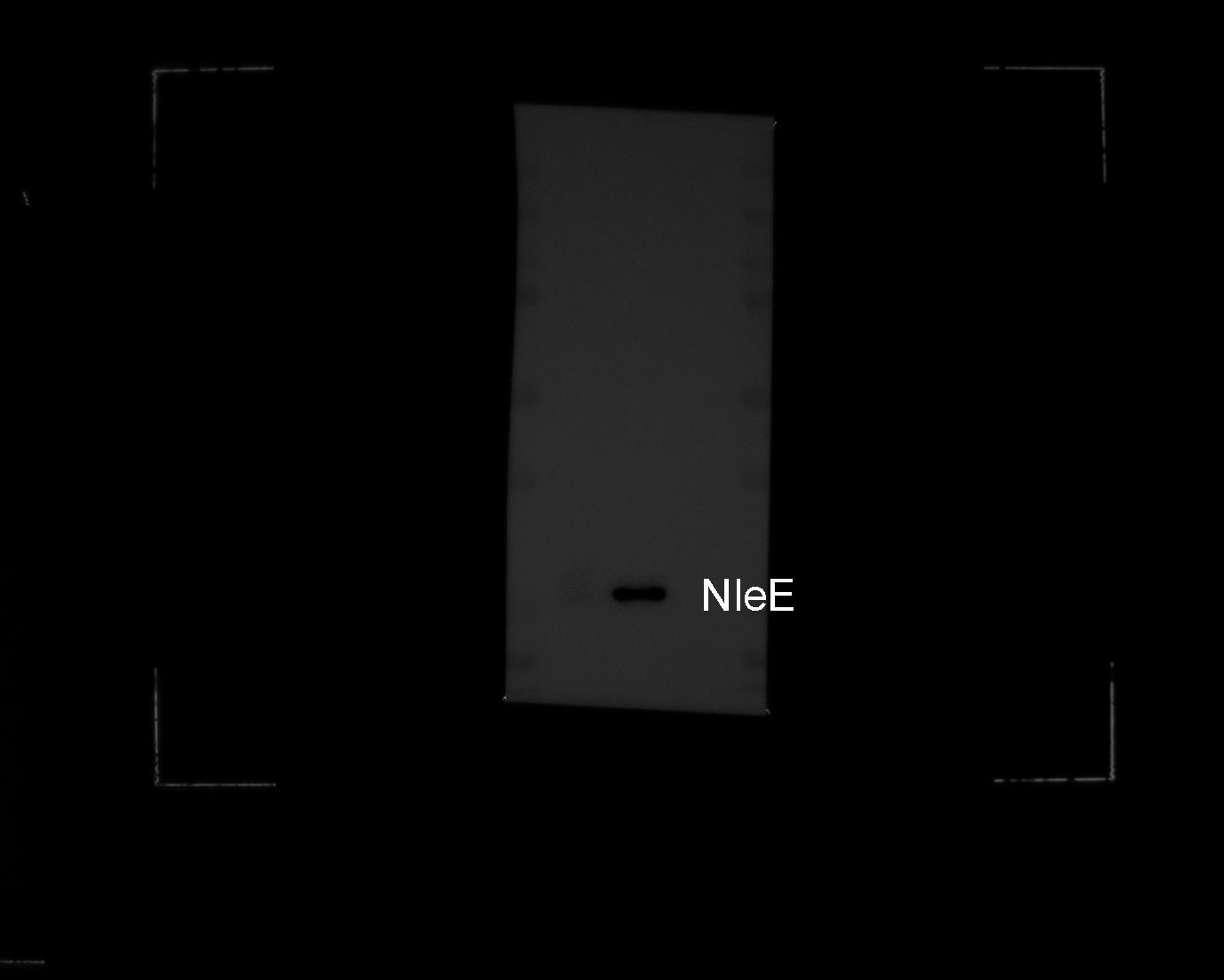

Supplement: Figure 3—figure supplement 1—source data 2. [file elife-69047-fig3-figsupp1-data2.zip › Figure 3-figure supplement 1-source data 2. Original western blot files for Figure 3-figure supplement 1/Figure 3-figure supplement 1-source data B1.jpg]

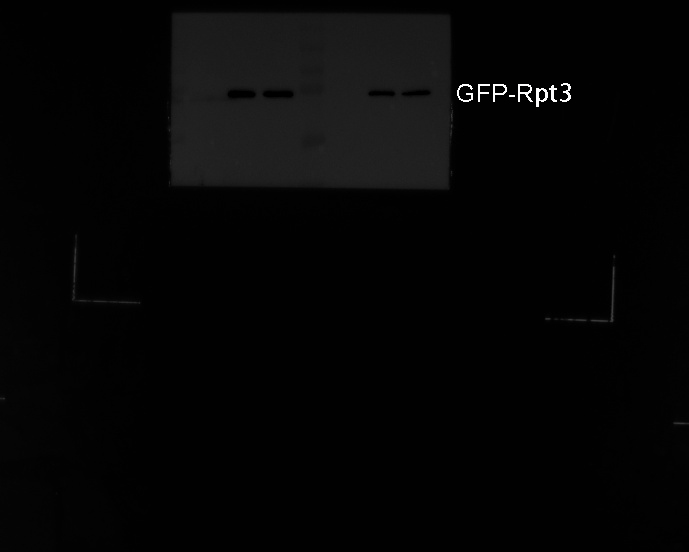

Supplement: Figure 3—figure supplement 1—source data 2. [file elife-69047-fig3-figsupp1-data2.zip › Figure 3-figure supplement 1-source data 2. Original western blot files for Figure 3-figure supplement 1/Figure 3-figure supplement 1-source data B2.jpg]

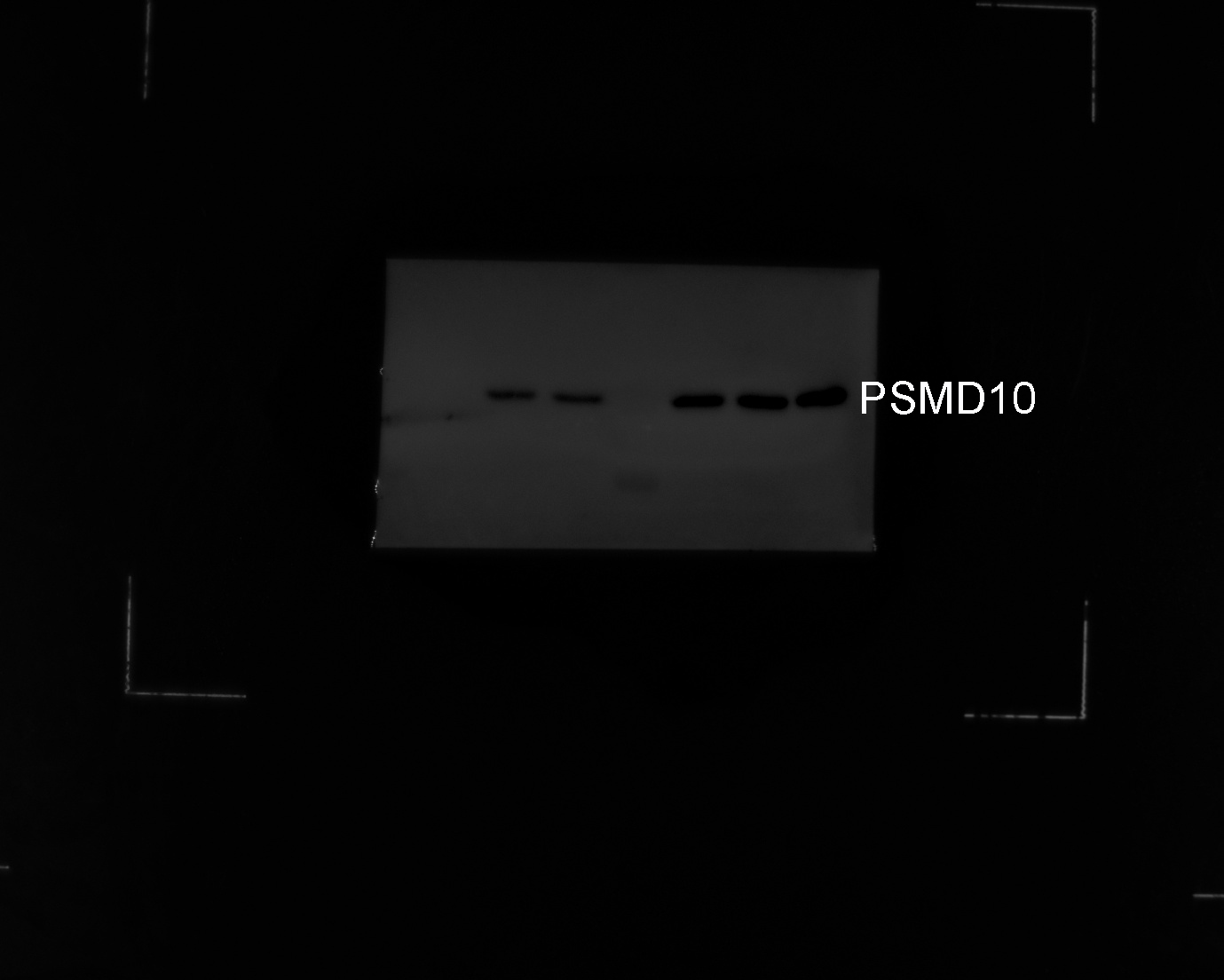

Supplement: Figure 3—figure supplement 1—source data 2. [file elife-69047-fig3-figsupp1-data2.zip › Figure 3-figure supplement 1-source data 2. Original western blot files for Figure 3-figure supplement 1/Figure 3-figure supplement 1-source data B3.jpg]

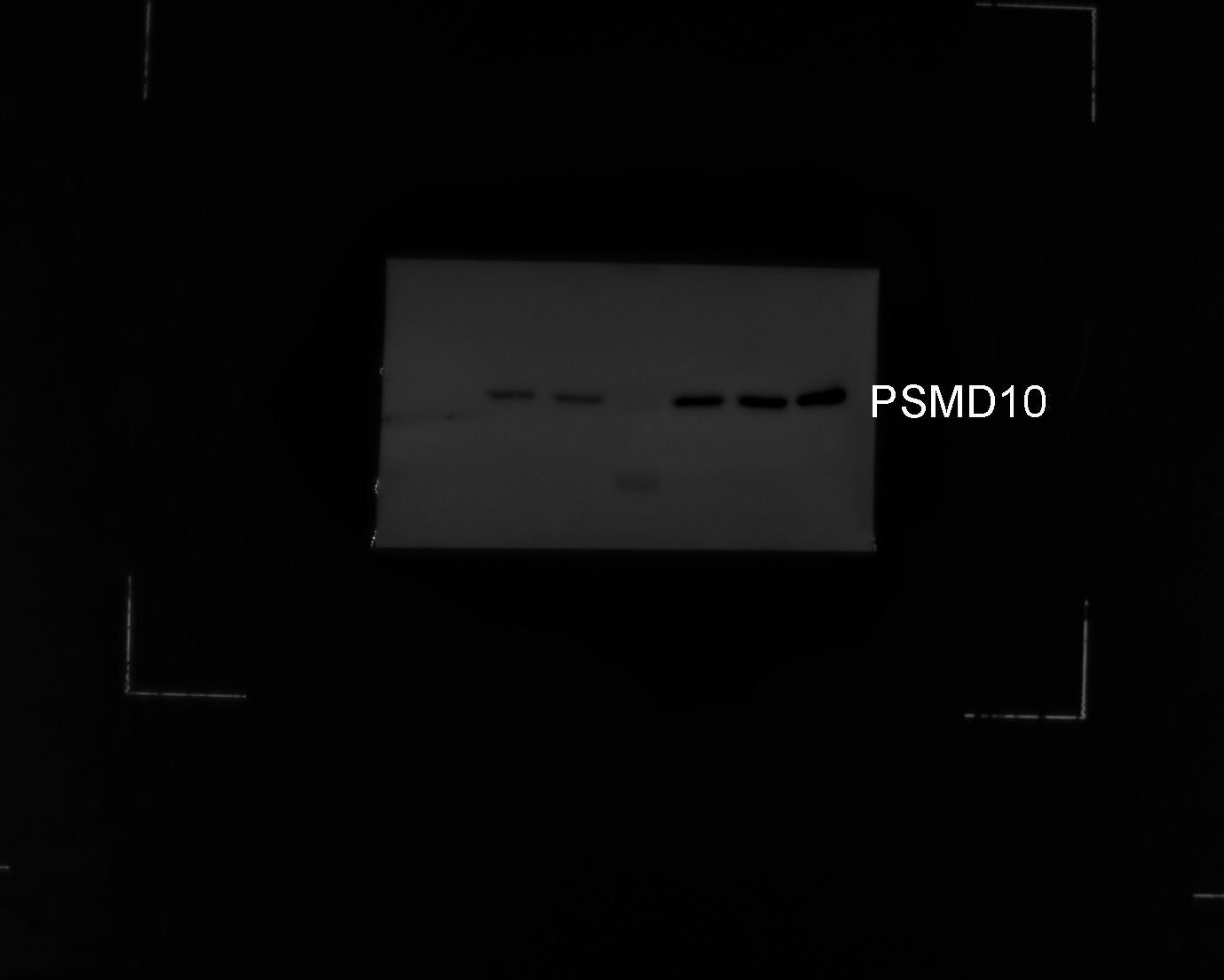

Supplement: Figure 3—figure supplement 1—source data 2. [file elife-69047-fig3-figsupp1-data2.zip › Figure 3-figure supplement 1-source data 2. Original western blot files for Figure 3-figure supplement 1/Figure 3-figure supplement 1-source data B4.jpg]

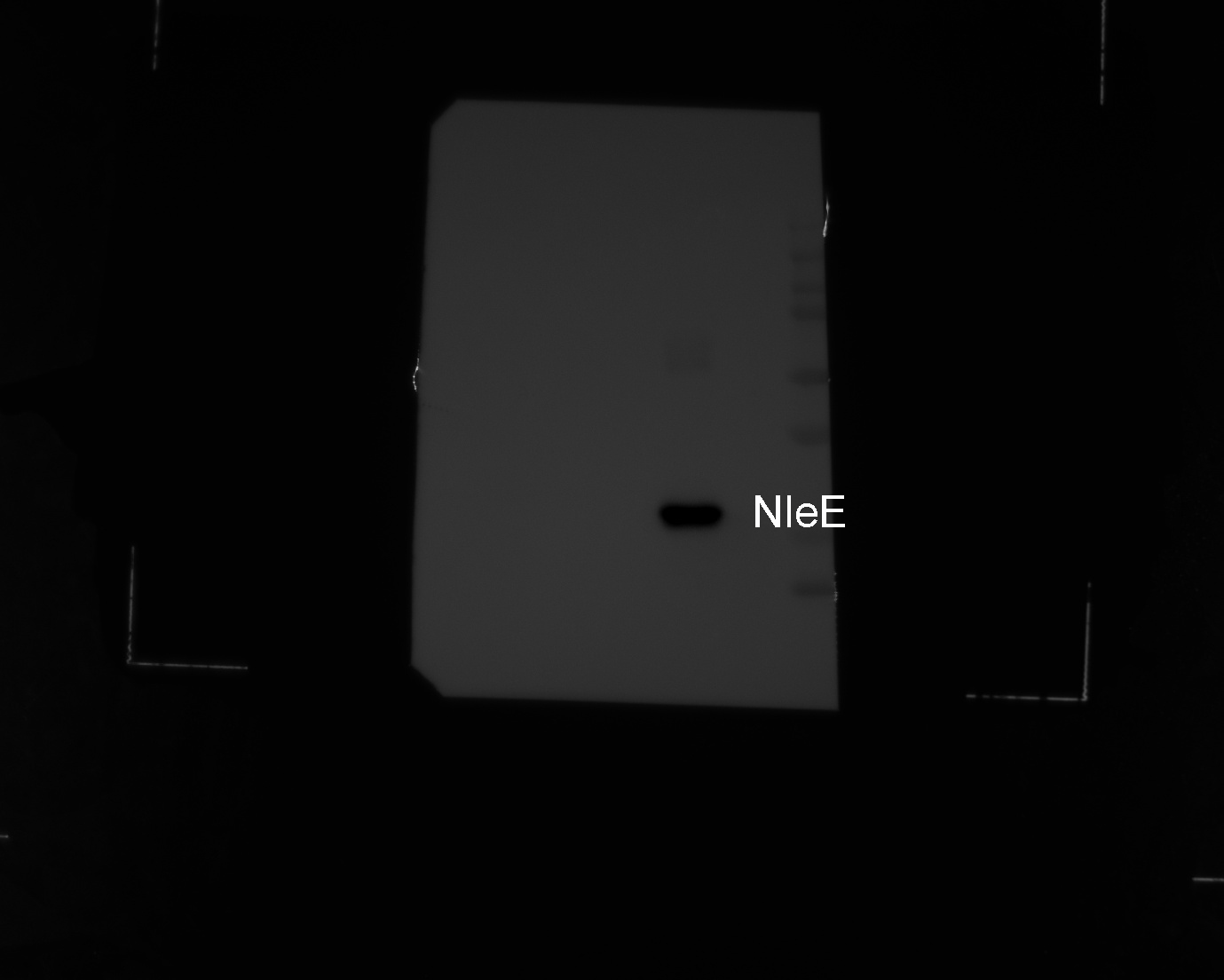

Supplement: Figure 3—figure supplement 1—source data 2. [file elife-69047-fig3-figsupp1-data2.zip › Figure 3-figure supplement 1-source data 2. Original western blot files for Figure 3-figure supplement 1/Figure 3-figure supplement 1-source data C1.jpg]

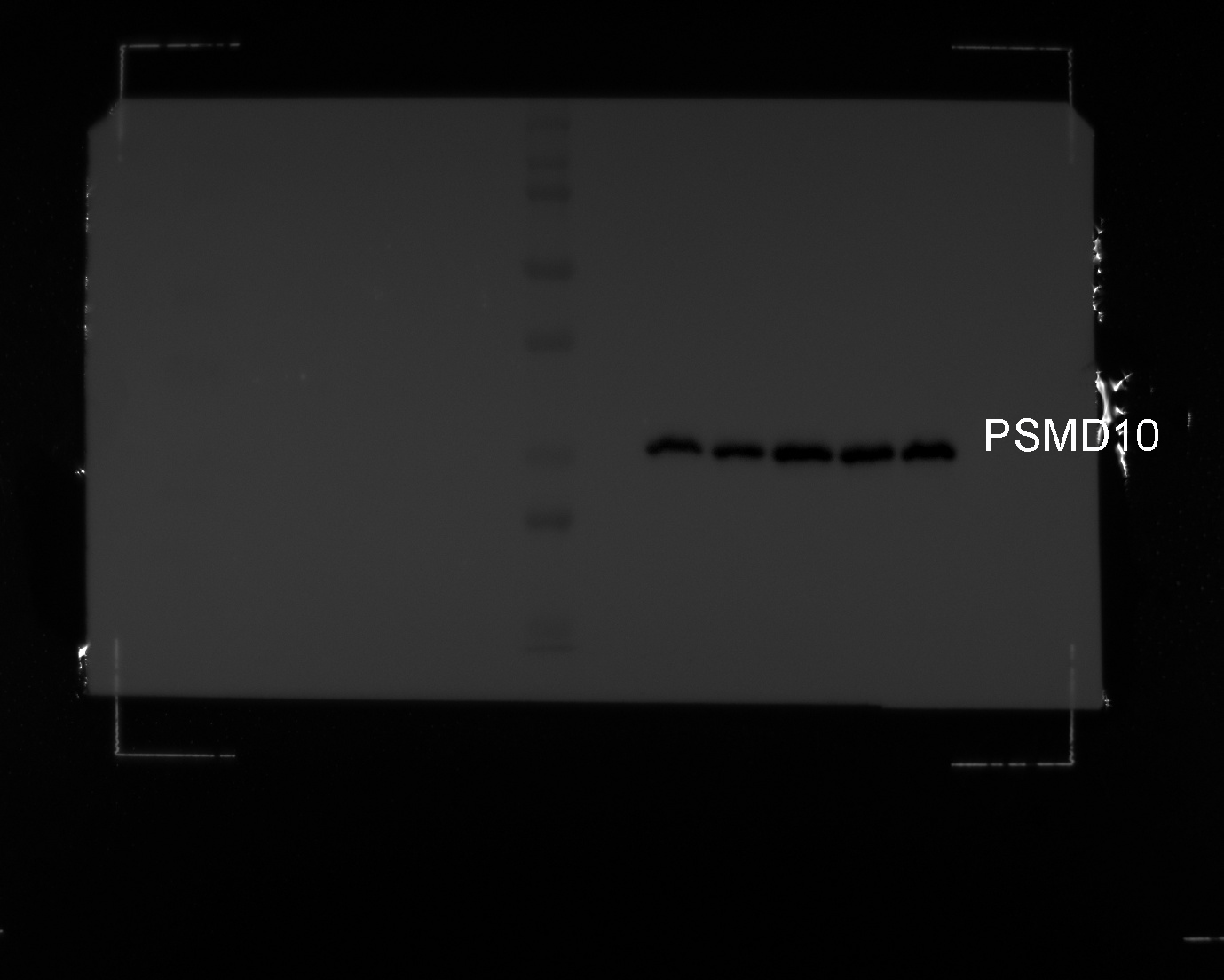

Supplement: Figure 3—figure supplement 1—source data 2. [file elife-69047-fig3-figsupp1-data2.zip › Figure 3-figure supplement 1-source data 2. Original western blot files for Figure 3-figure supplement 1/Figure 3-figure supplement 1-source data C2.jpg]

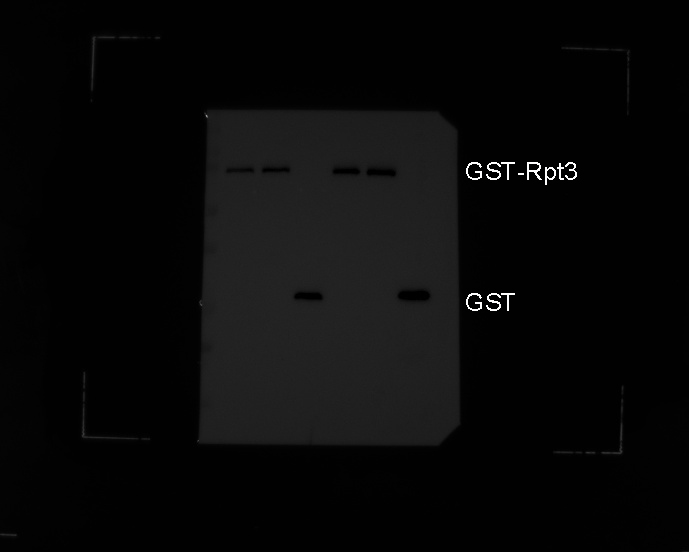

Supplement: Figure 3—figure supplement 1—source data 2. [file elife-69047-fig3-figsupp1-data2.zip › Figure 3-figure supplement 1-source data 2. Original western blot files for Figure 3-figure supplement 1/Figure 3-figure supplement 1-source data C3.jpg]

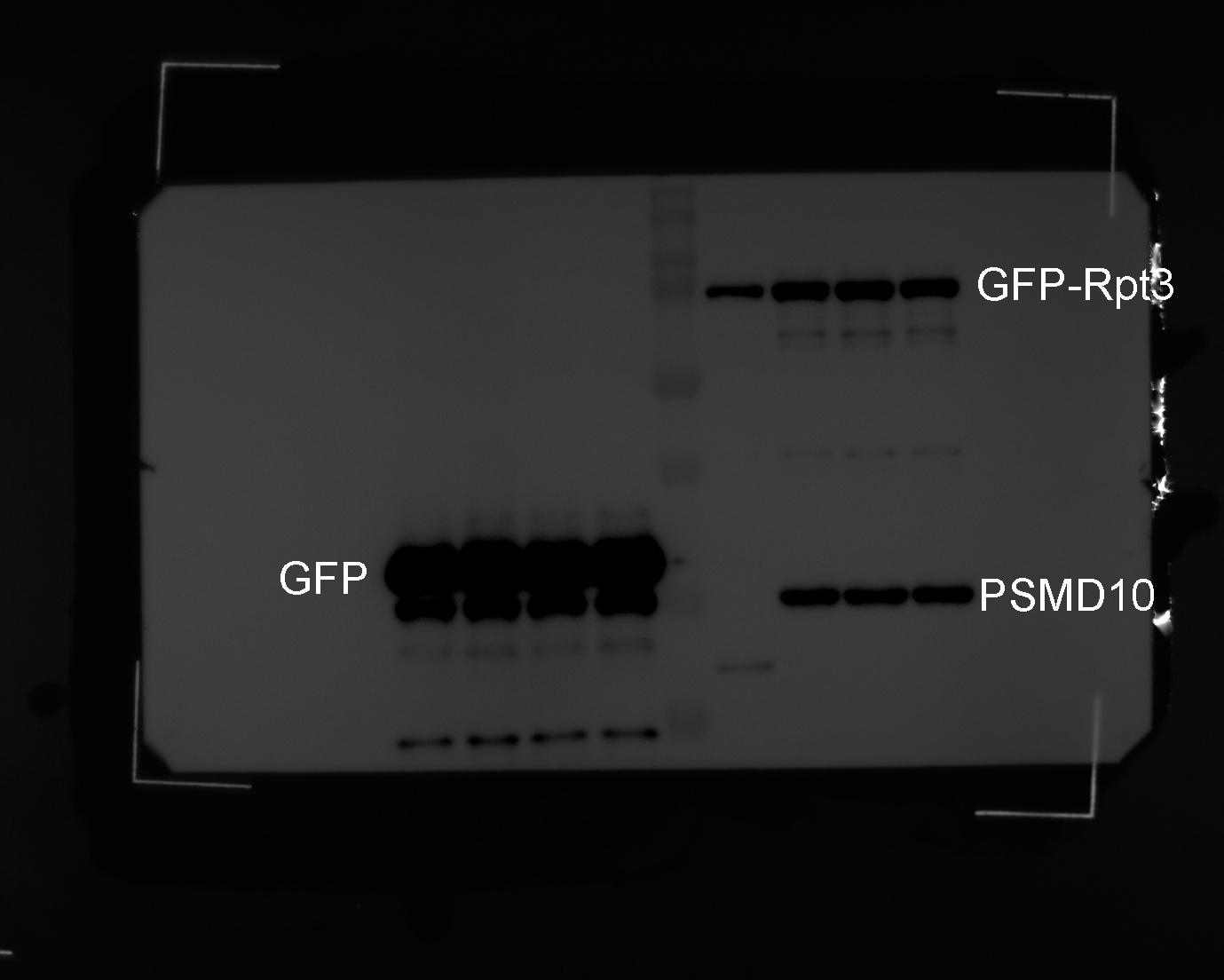

Supplement: Figure 3—figure supplement 1—source data 2. [file elife-69047-fig3-figsupp1-data2.zip › Figure 3-figure supplement 1-source data 2. Original western blot files for Figure 3-figure supplement 1/Figure 3-figure supplement 1-source data D1.jpg]

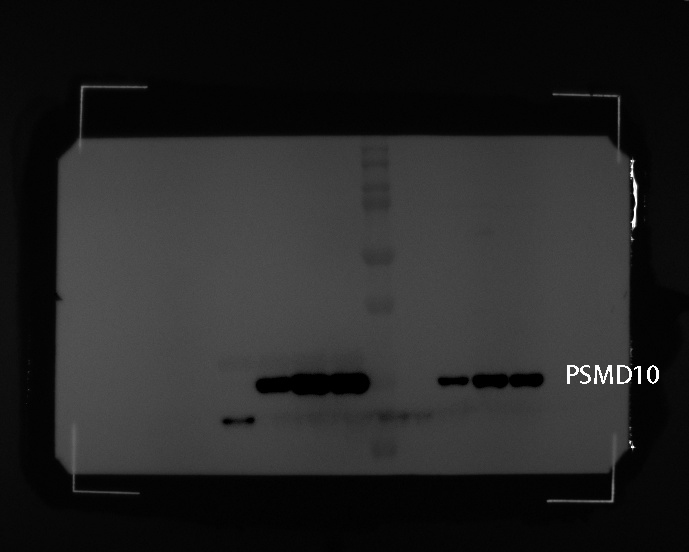

Supplement: Figure 3—figure supplement 1—source data 2. [file elife-69047-fig3-figsupp1-data2.zip › Figure 3-figure supplement 1-source data 2. Original western blot files for Figure 3-figure supplement 1/Figure 3-figure supplement 1-source data D2.jpg]

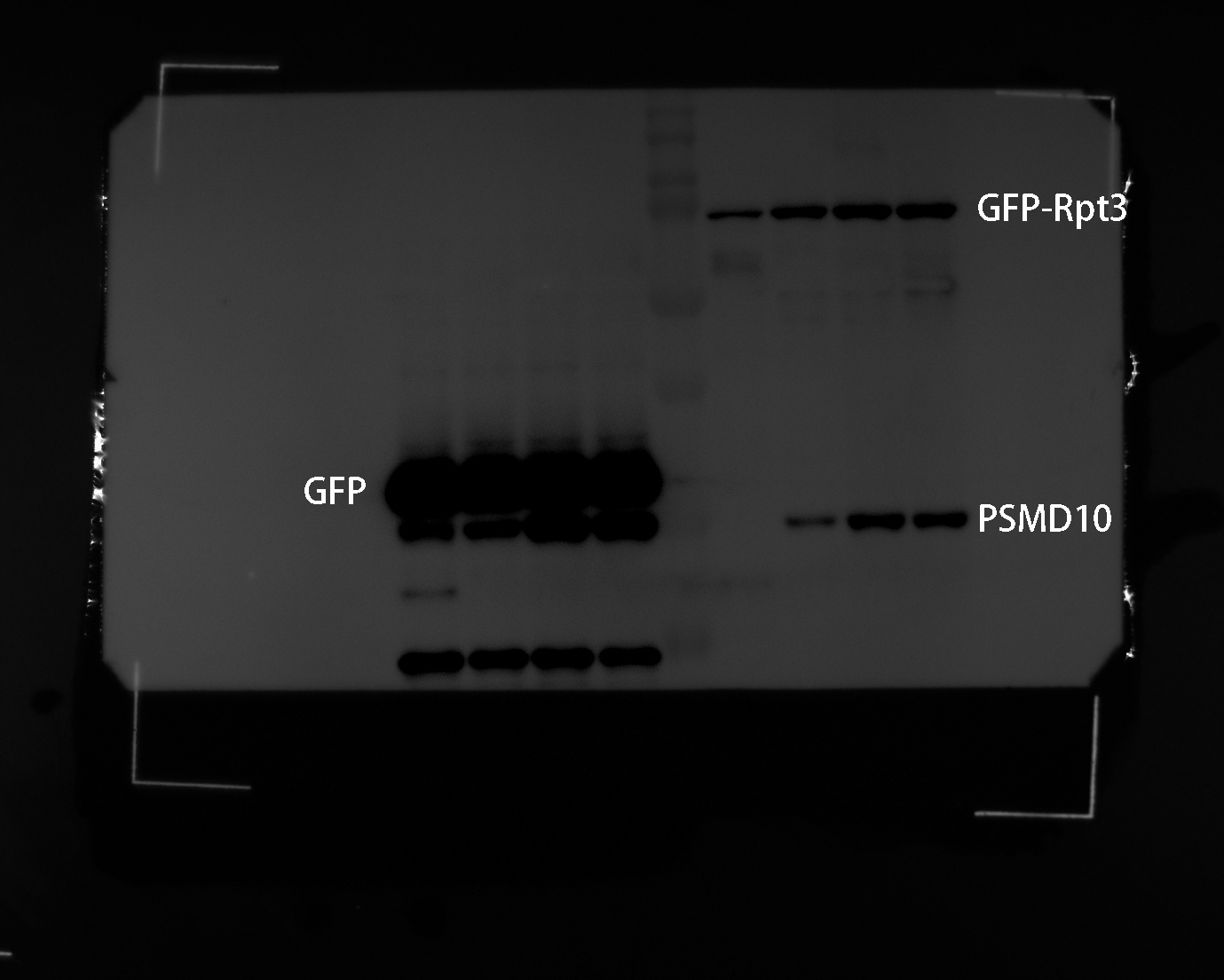

Supplement: Figure 3—figure supplement 1—source data 2. [file elife-69047-fig3-figsupp1-data2.zip › Figure 3-figure supplement 1-source data 2. Original western blot files for Figure 3-figure supplement 1/Figure 3-figure supplement 1-source data D3.jpg]

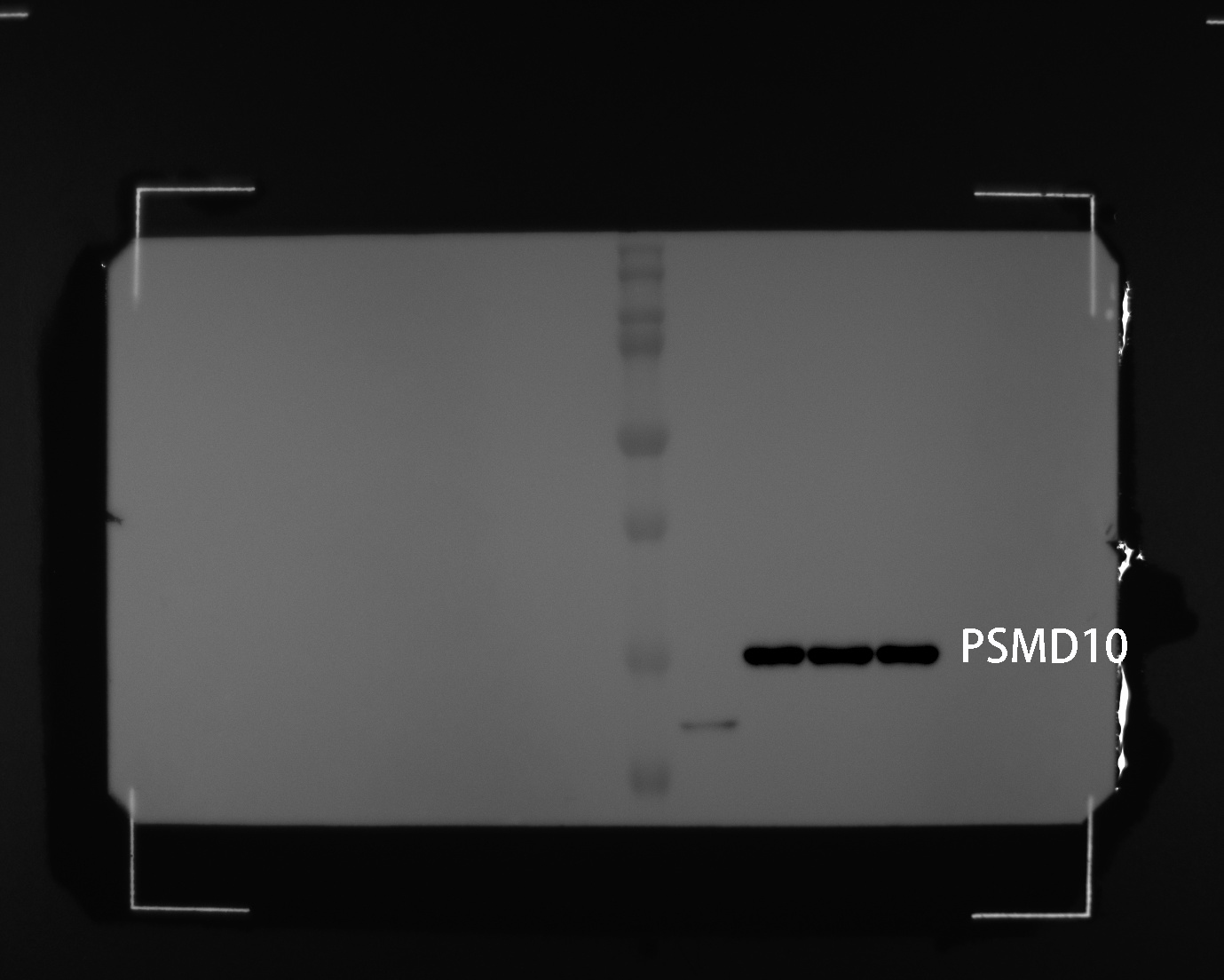

Supplement: Figure 3—figure supplement 1—source data 2. [file elife-69047-fig3-figsupp1-data2.zip › Figure 3-figure supplement 1-source data 2. Original western blot files for Figure 3-figure supplement 1/Figure 3-figure supplement 1-source data D4.jpg]

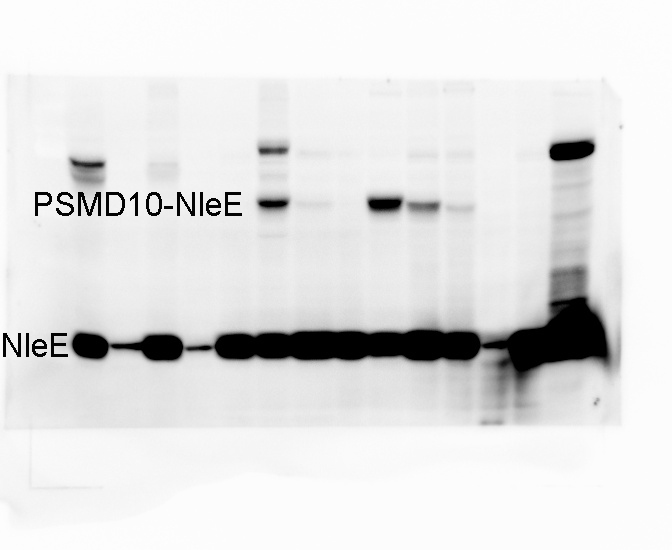

Supplement: Figure 4—source data 1. [file elife-69047-fig4-data1.zip › Figure 4-source data 1. Original western blot files for Figure 4/Figure 4-source data B1.jpg]

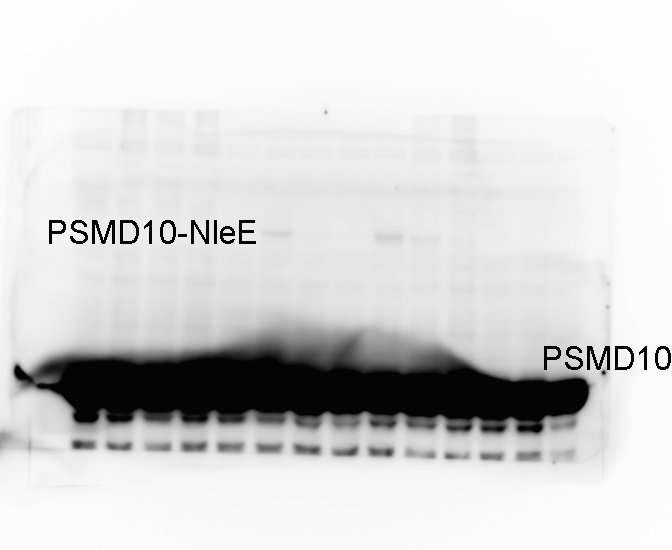

Supplement: Figure 4—source data 1. [file elife-69047-fig4-data1.zip › Figure 4-source data 1. Original western blot files for Figure 4/Figure 4-source data B2.jpg]

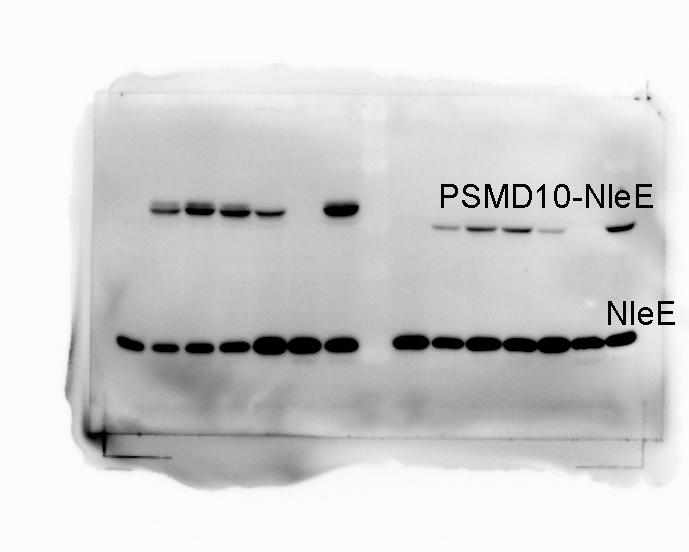

Supplement: Figure 4—source data 1. [file elife-69047-fig4-data1.zip › Figure 4-source data 1. Original western blot files for Figure 4/Figure 4-source data D1.jpg]

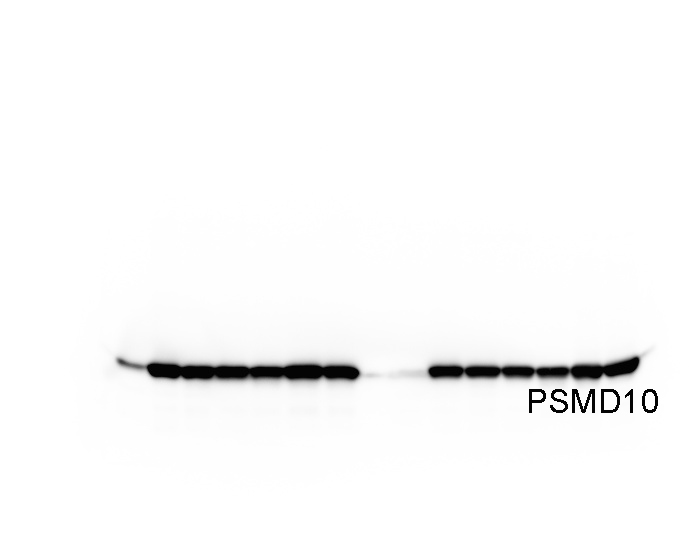

Supplement: Figure 4—source data 1. [file elife-69047-fig4-data1.zip › Figure 4-source data 1. Original western blot files for Figure 4/Figure 4-source data D2.jpg]

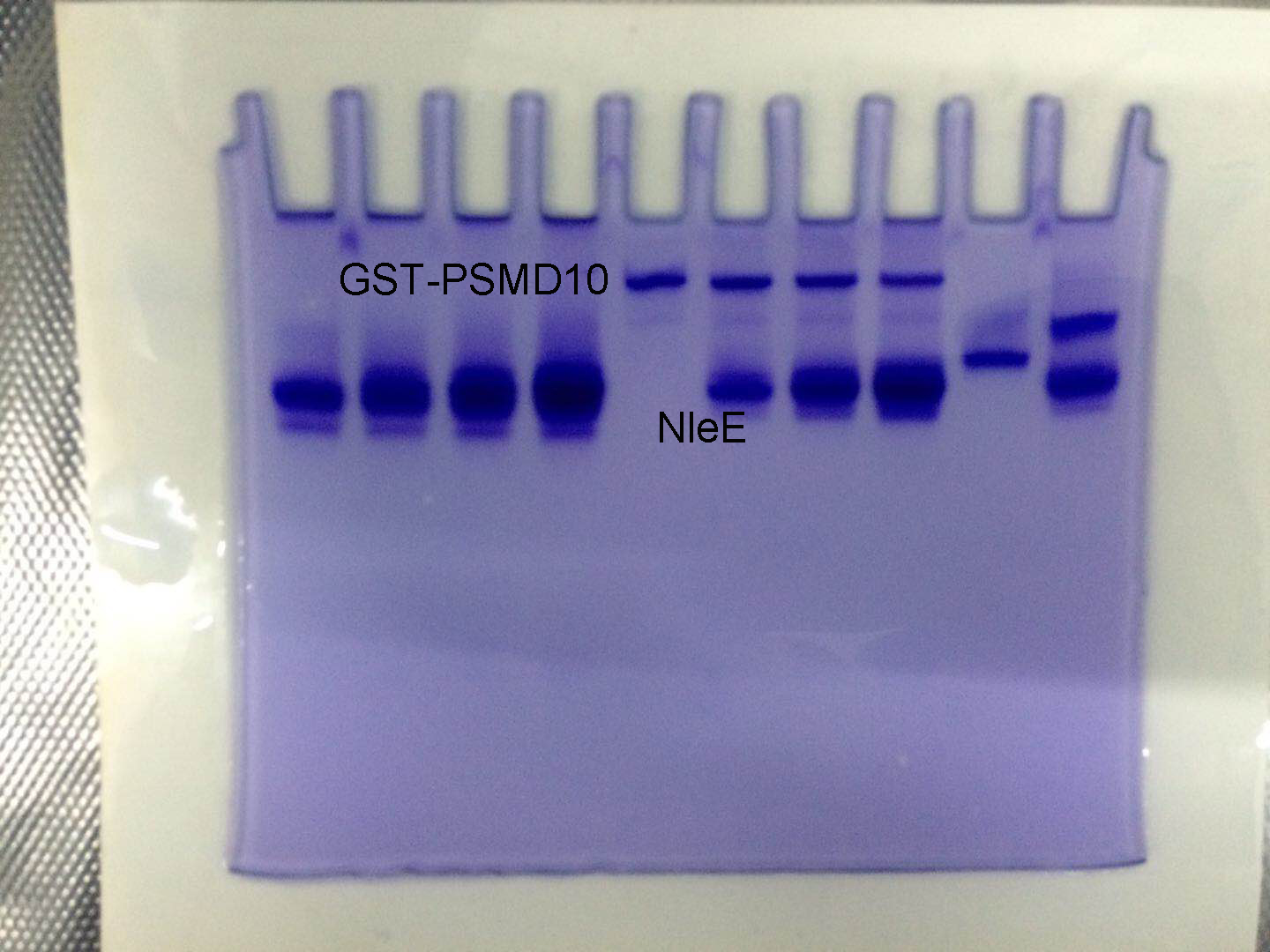

Supplement: Figure 4—figure supplement 1—source data 3. [file elife-69047-fig4-figsupp1-data3.zip › Figure 4-figure supplement 1-source data 3. Original western blot files for Figure 4-figure supplement 1/Figure 4-figure supplement 1-source data A.jpg]

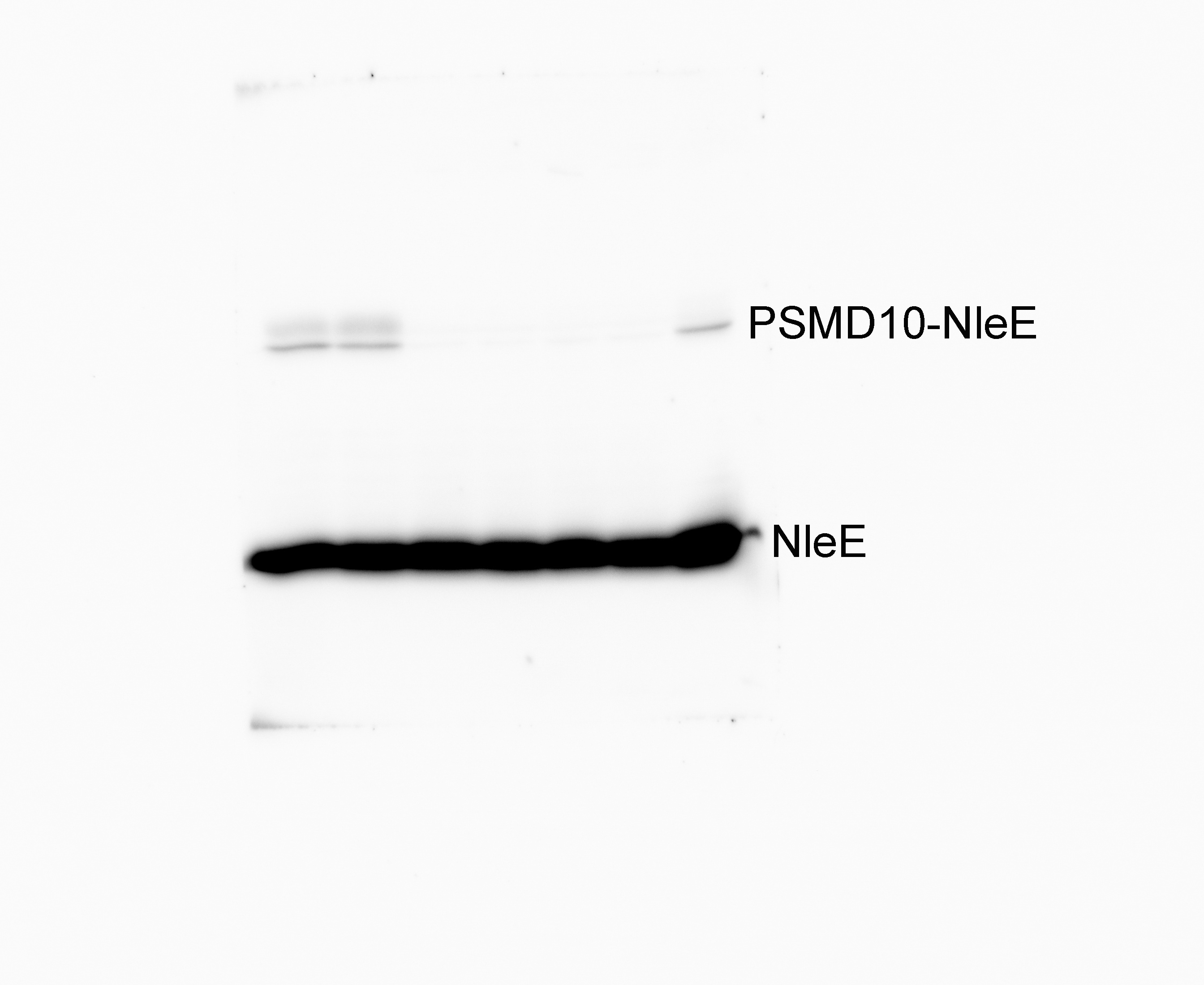

Supplement: Figure 4—figure supplement 1—source data 3. [file elife-69047-fig4-figsupp1-data3.zip › Figure 4-figure supplement 1-source data 3. Original western blot files for Figure 4-figure supplement 1/Figure 4-figure supplement 1-source data D.jpg]

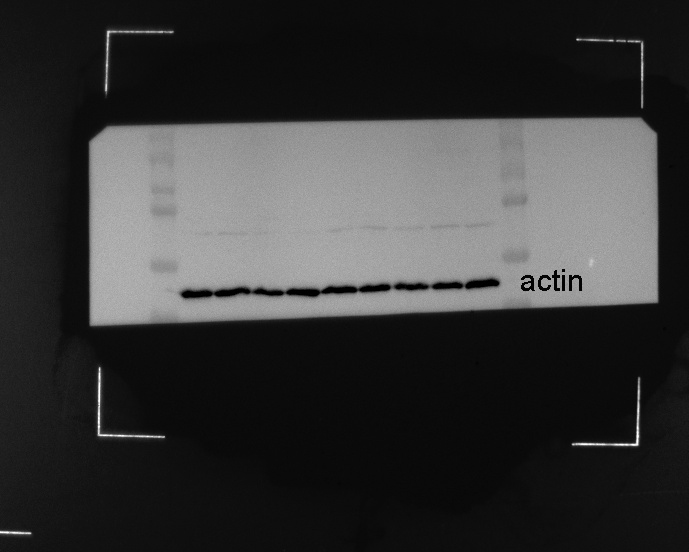

Supplement: Figure 4—figure supplement 2—source data 2. [file elife-69047-fig4-figsupp2-data2.zip › Figure 4-figure supplement 2-source data 2. Original western blot files for Figure 4-figure supplement 2/Figure 4-figure supplement 2-source data A1.jpg]

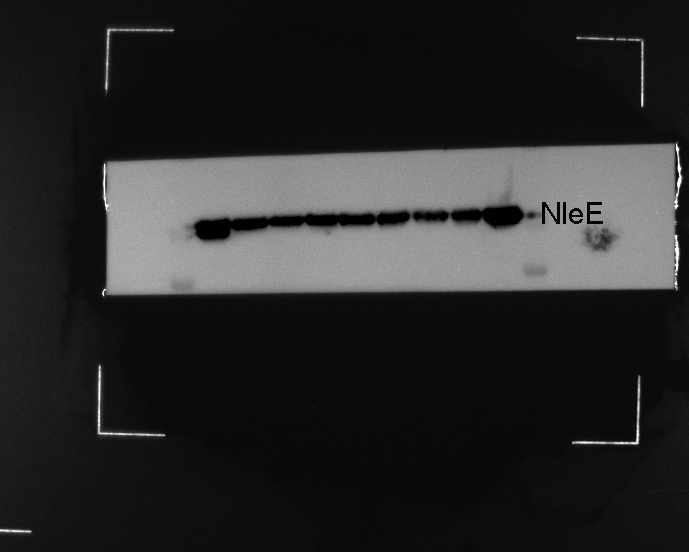

Supplement: Figure 4—figure supplement 2—source data 2. [file elife-69047-fig4-figsupp2-data2.zip › Figure 4-figure supplement 2-source data 2. Original western blot files for Figure 4-figure supplement 2/Figure 4-figure supplement 2-source data A2.jpg]

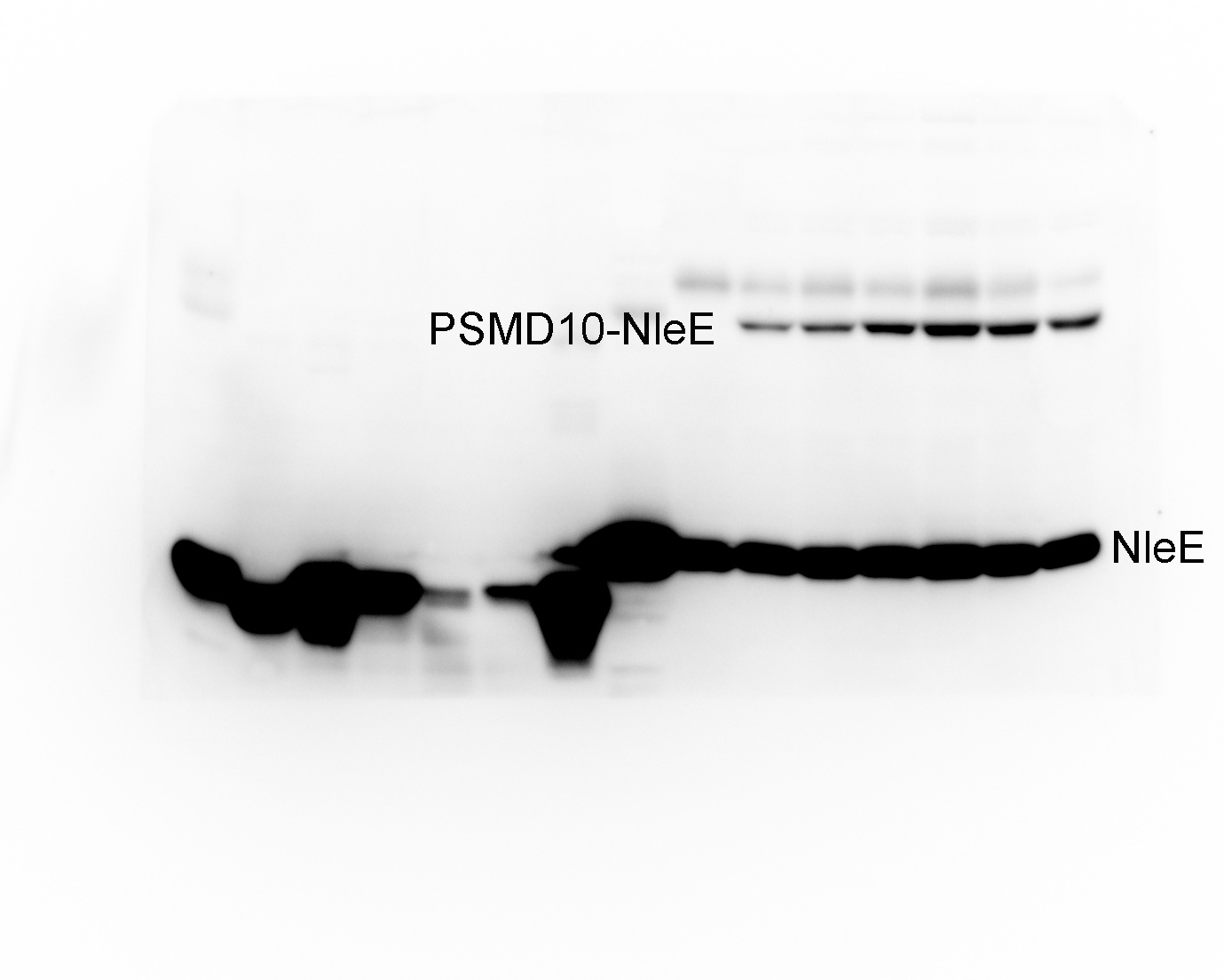

Supplement: Figure 4—figure supplement 2—source data 2. [file elife-69047-fig4-figsupp2-data2.zip › Figure 4-figure supplement 2-source data 2. Original western blot files for Figure 4-figure supplement 2/Figure 4-figure supplement 2-source data C1.jpg]

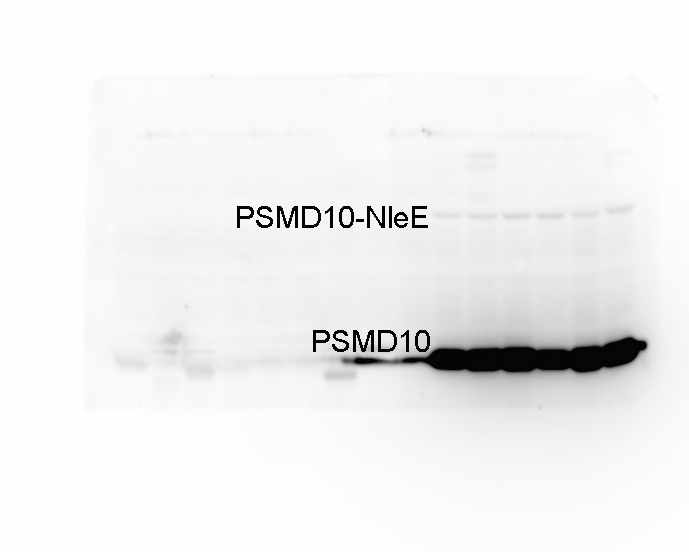

Supplement: Figure 4—figure supplement 2—source data 2. [file elife-69047-fig4-figsupp2-data2.zip › Figure 4-figure supplement 2-source data 2. Original western blot files for Figure 4-figure supplement 2/Figure 4-figure supplement 2-source data C2.jpg]

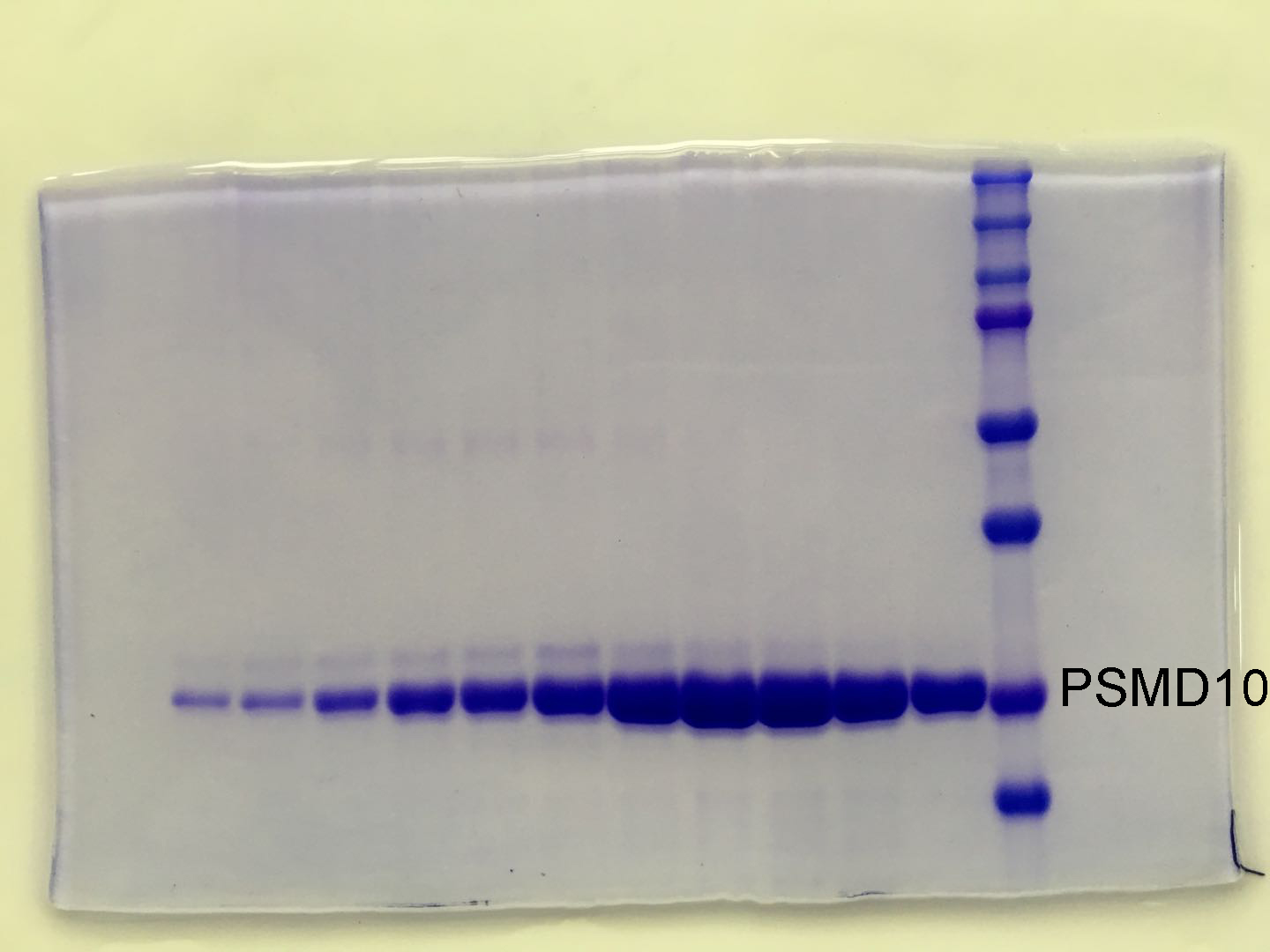

Supplement: Figure 5—source data 2. [file elife-69047-fig5-data2.zip › Figure 5-source data 2. Original western blot files for Figure 5/Figure 5-source data A.jpg]

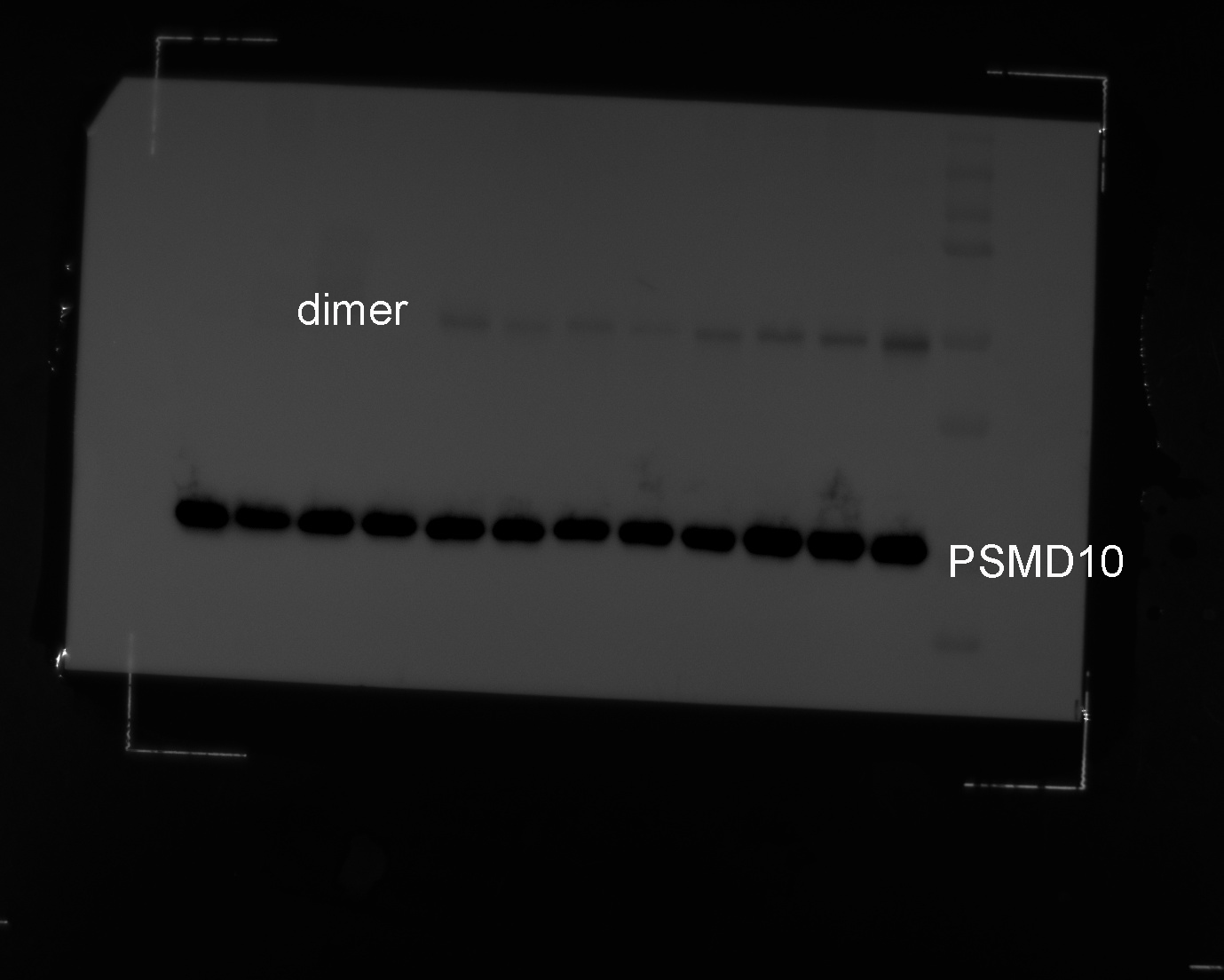

Supplement: Figure 5—source data 2. [file elife-69047-fig5-data2.zip › Figure 5-source data 2. Original western blot files for Figure 5/Figure 5-source data B.jpg]

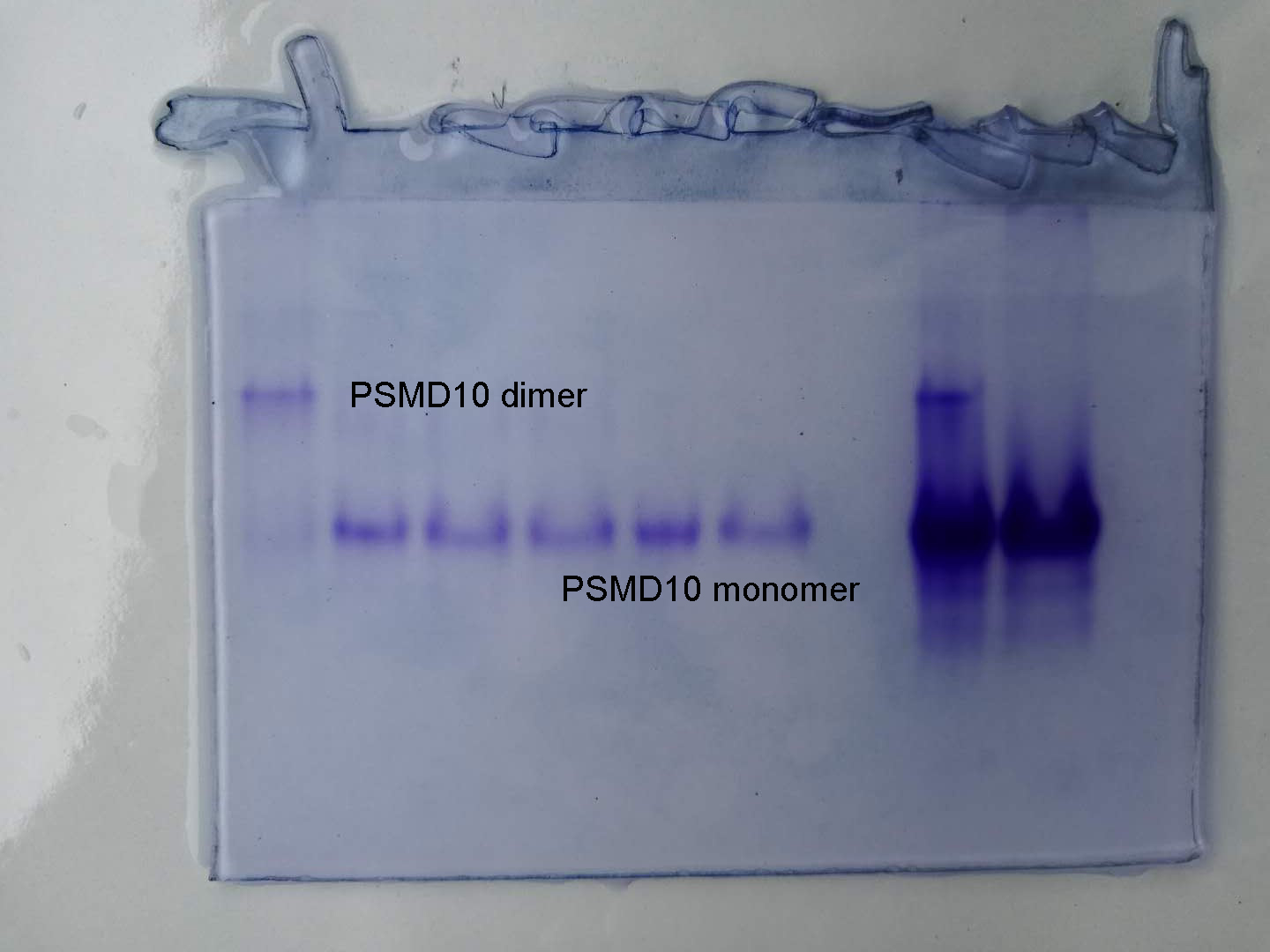

Supplement: Figure 5—source data 2. [file elife-69047-fig5-data2.zip › Figure 5-source data 2. Original western blot files for Figure 5/Figure 5-source data C.jpg]

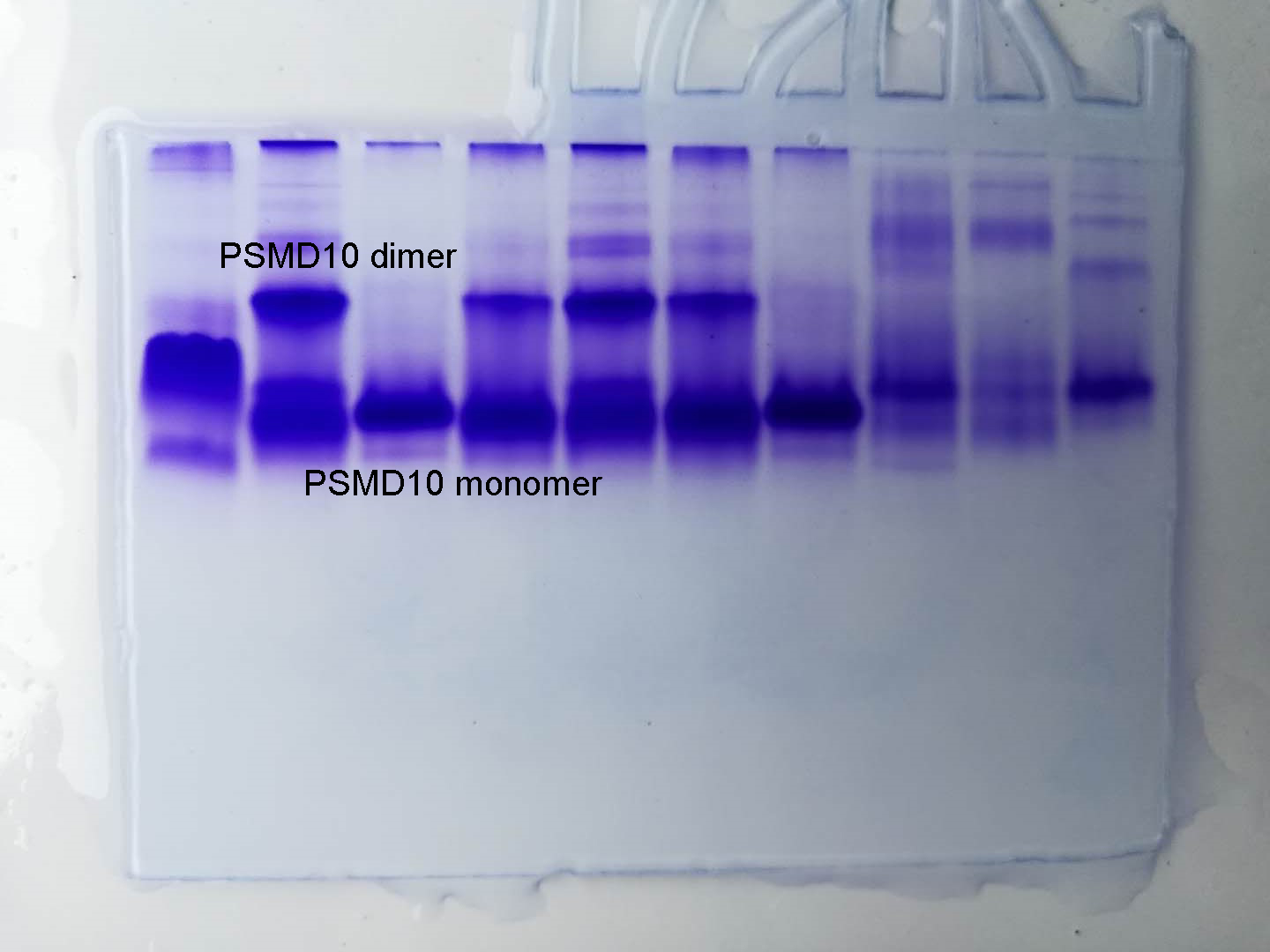

Supplement: Figure 5—source data 2. [file elife-69047-fig5-data2.zip › Figure 5-source data 2. Original western blot files for Figure 5/Figure 5-source data D.jpg]

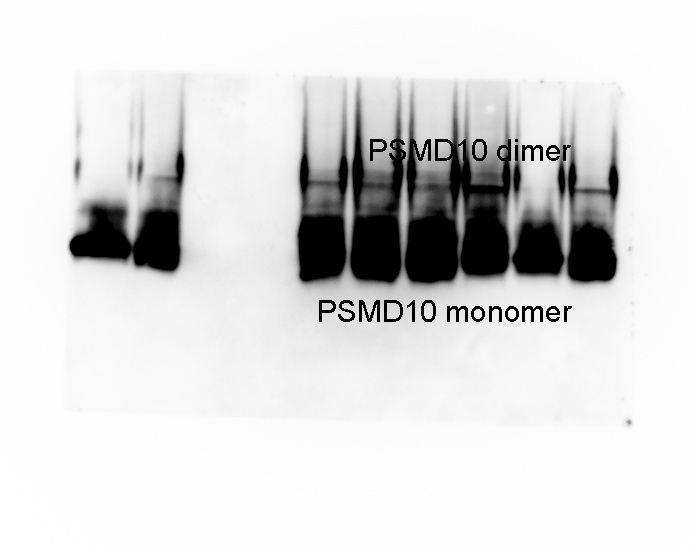

Supplement: Figure 5—source data 2. [file elife-69047-fig5-data2.zip › Figure 5-source data 2. Original western blot files for Figure 5/Figure 5-source data E.jpg]

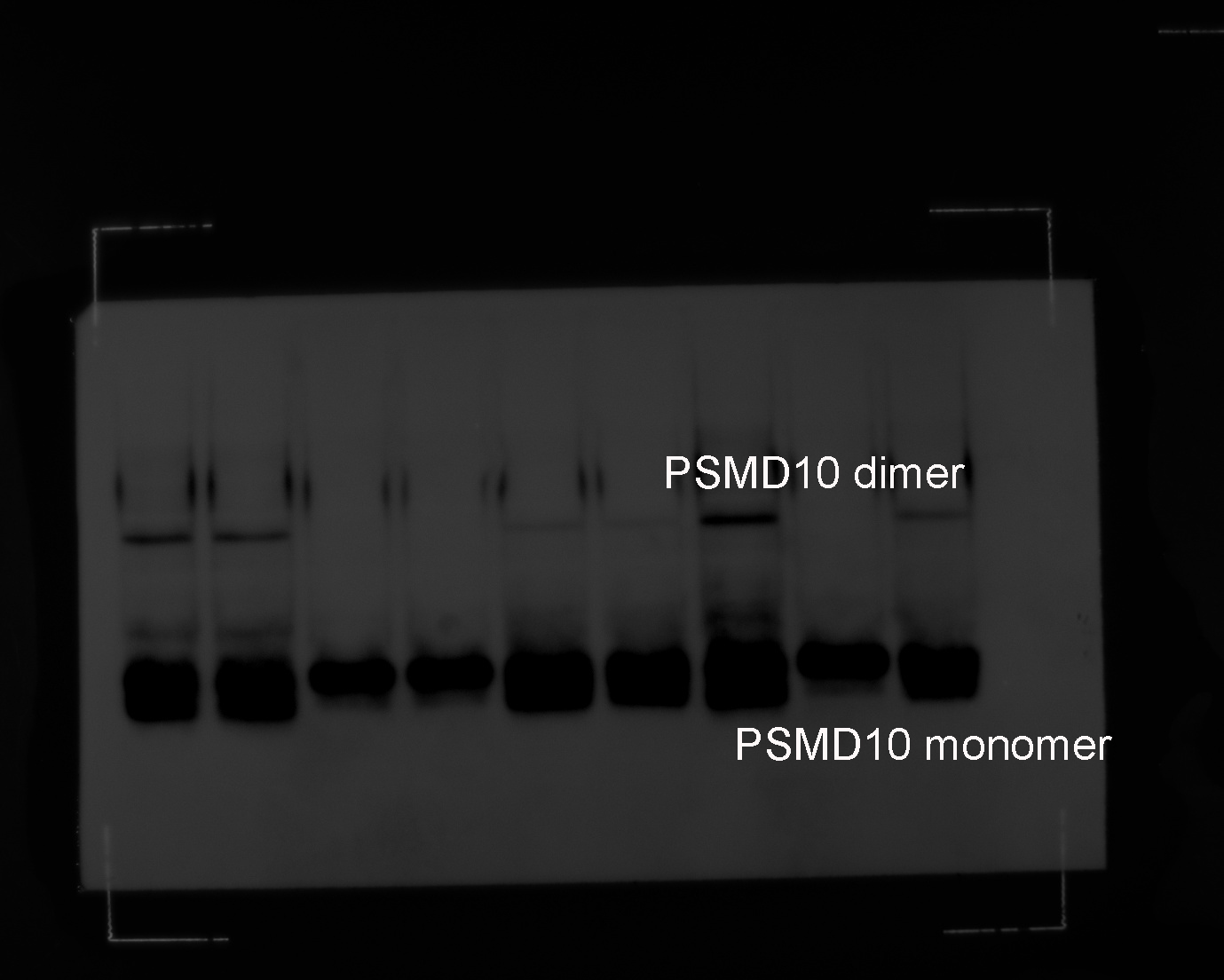

Supplement: Figure 5—source data 2. [file elife-69047-fig5-data2.zip › Figure 5-source data 2. Original western blot files for Figure 5/Figure 5-source data F1.jpg]

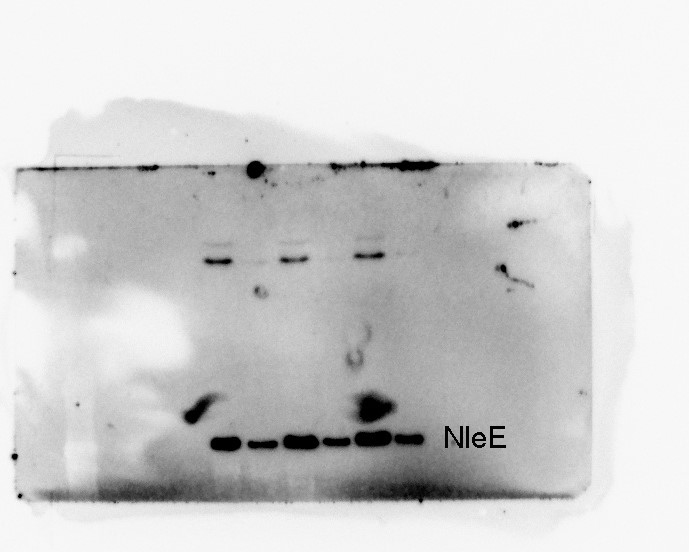

Supplement: Figure 5—source data 2. [file elife-69047-fig5-data2.zip › Figure 5-source data 2. Original western blot files for Figure 5/Figure 5-source data F2.jpg]

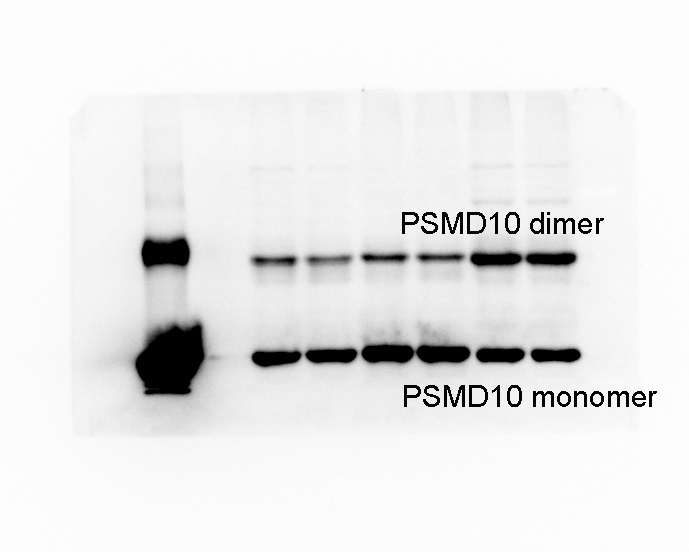

Supplement: Figure 5—source data 2. [file elife-69047-fig5-data2.zip › Figure 5-source data 2. Original western blot files for Figure 5/Figure 5-source data G1.jpg]

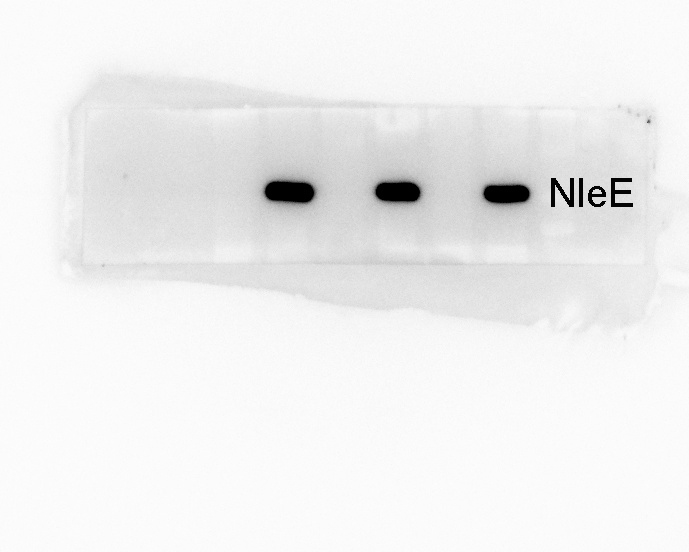

Supplement: Figure 5—source data 2. [file elife-69047-fig5-data2.zip › Figure 5-source data 2. Original western blot files for Figure 5/Figure 5-source data G2.jpg]

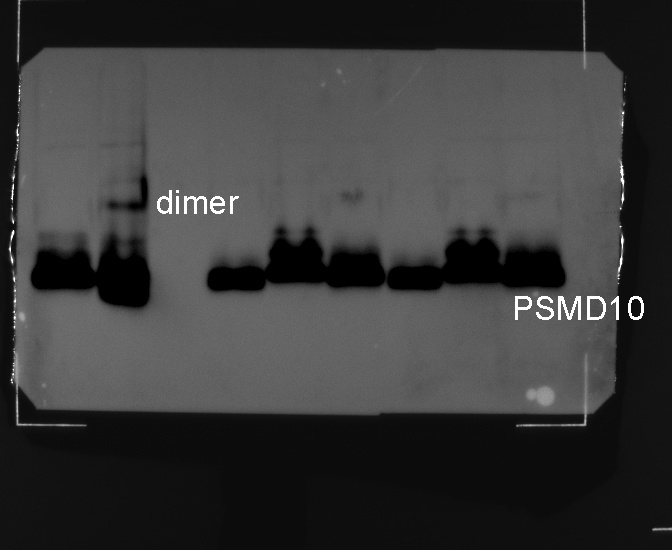

Supplement: Figure 5—figure supplement 1—source data 1. [file elife-69047-fig5-figsupp1-data1.zip › Figure 5-figure supplement 1-source data 1. Original western blot files for Figure 5-figure supplement 1/Figure 5-figure supplement 1-source data A.jpg]

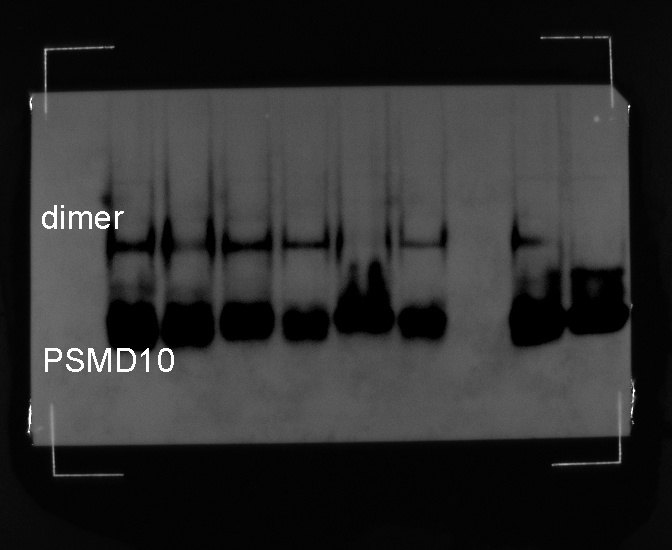

Supplement: Figure 5—figure supplement 1—source data 1. [file elife-69047-fig5-figsupp1-data1.zip › Figure 5-figure supplement 1-source data 1. Original western blot files for Figure 5-figure supplement 1/Figure 5-figure supplement 1-source data B.jpg]

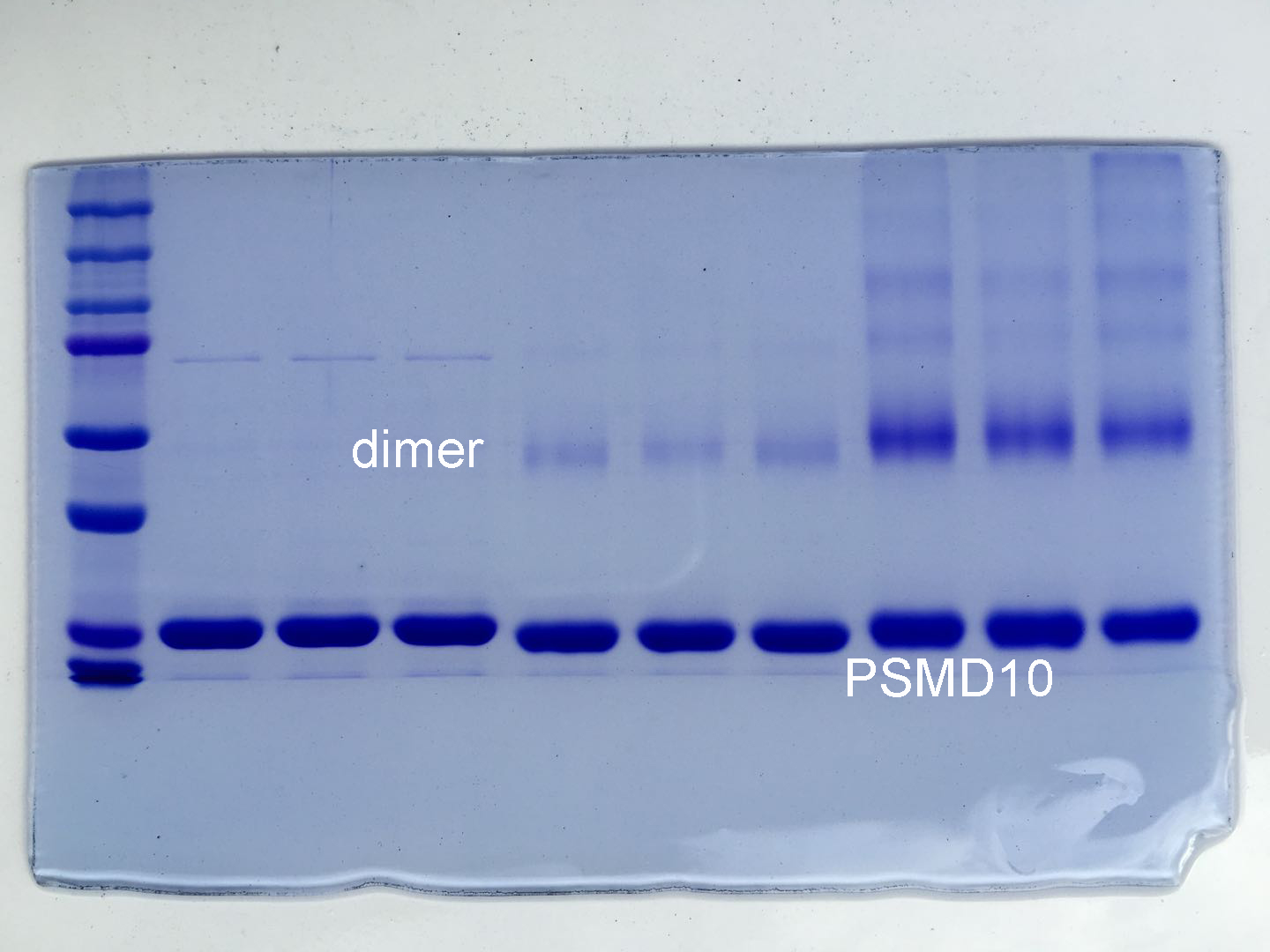

Supplement: Figure 5—figure supplement 1—source data 1. [file elife-69047-fig5-figsupp1-data1.zip › Figure 5-figure supplement 1-source data 1. Original western blot files for Figure 5-figure supplement 1/Figure 5-figure supplement 1-source data C.jpg]

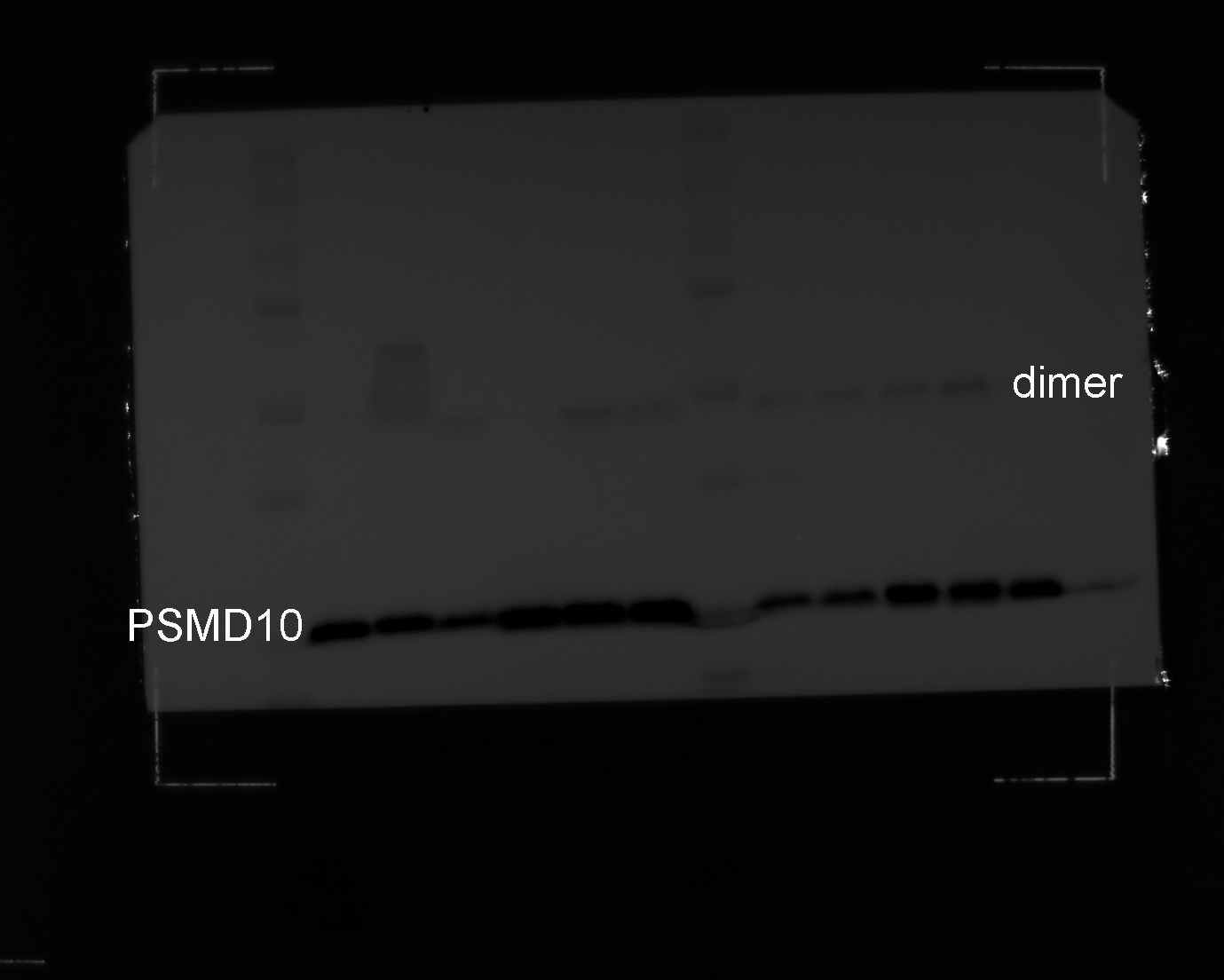

Supplement: Figure 5—figure supplement 1—source data 1. [file elife-69047-fig5-figsupp1-data1.zip › Figure 5-figure supplement 1-source data 1. Original western blot files for Figure 5-figure supplement 1/Figure 5-figure supplement 1-source data D.jpg]

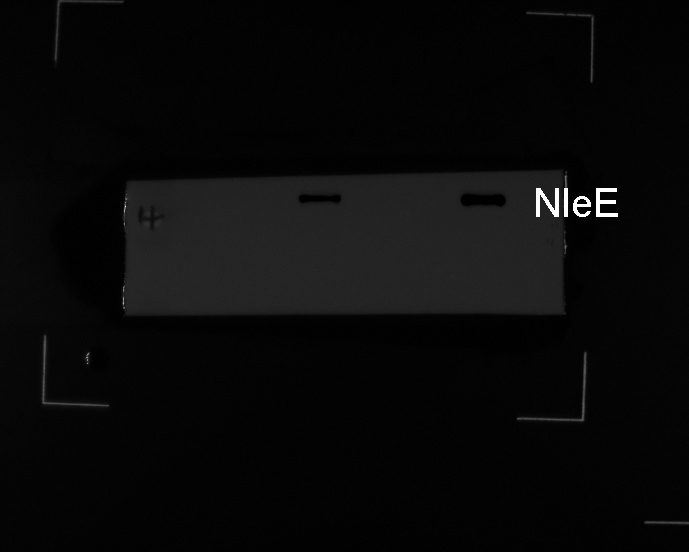

Supplement: Figure 6—source data 2. [file elife-69047-fig6-data2.zip › Figure 6-source data 2. Original western blot files for Figure 6/Figure 6-source data A1.jpg]

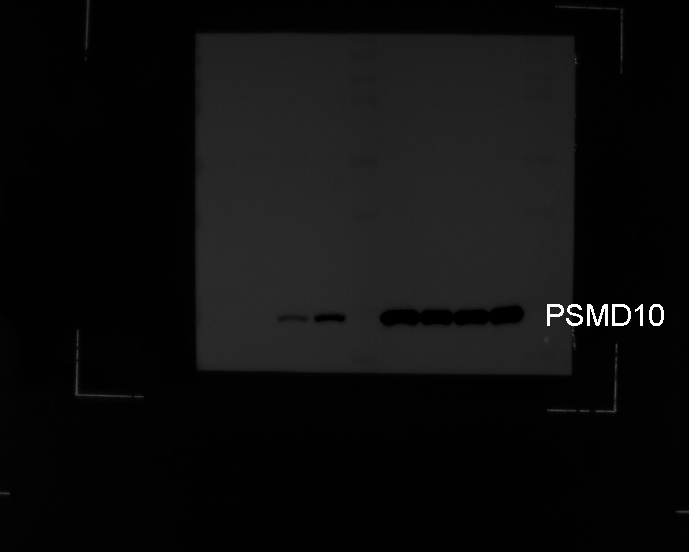

Supplement: Figure 6—source data 2. [file elife-69047-fig6-data2.zip › Figure 6-source data 2. Original western blot files for Figure 6/Figure 6-source data A2.jpg]

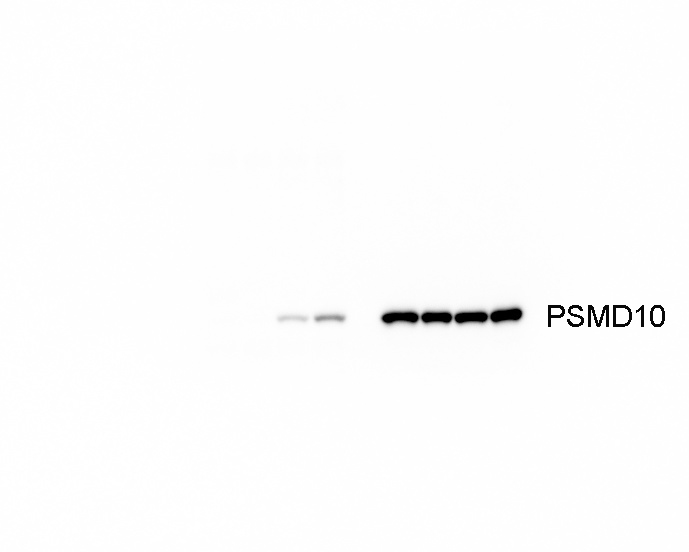

Supplement: Figure 6—source data 2. [file elife-69047-fig6-data2.zip › Figure 6-source data 2. Original western blot files for Figure 6/Figure 6-source data A3.jpg]

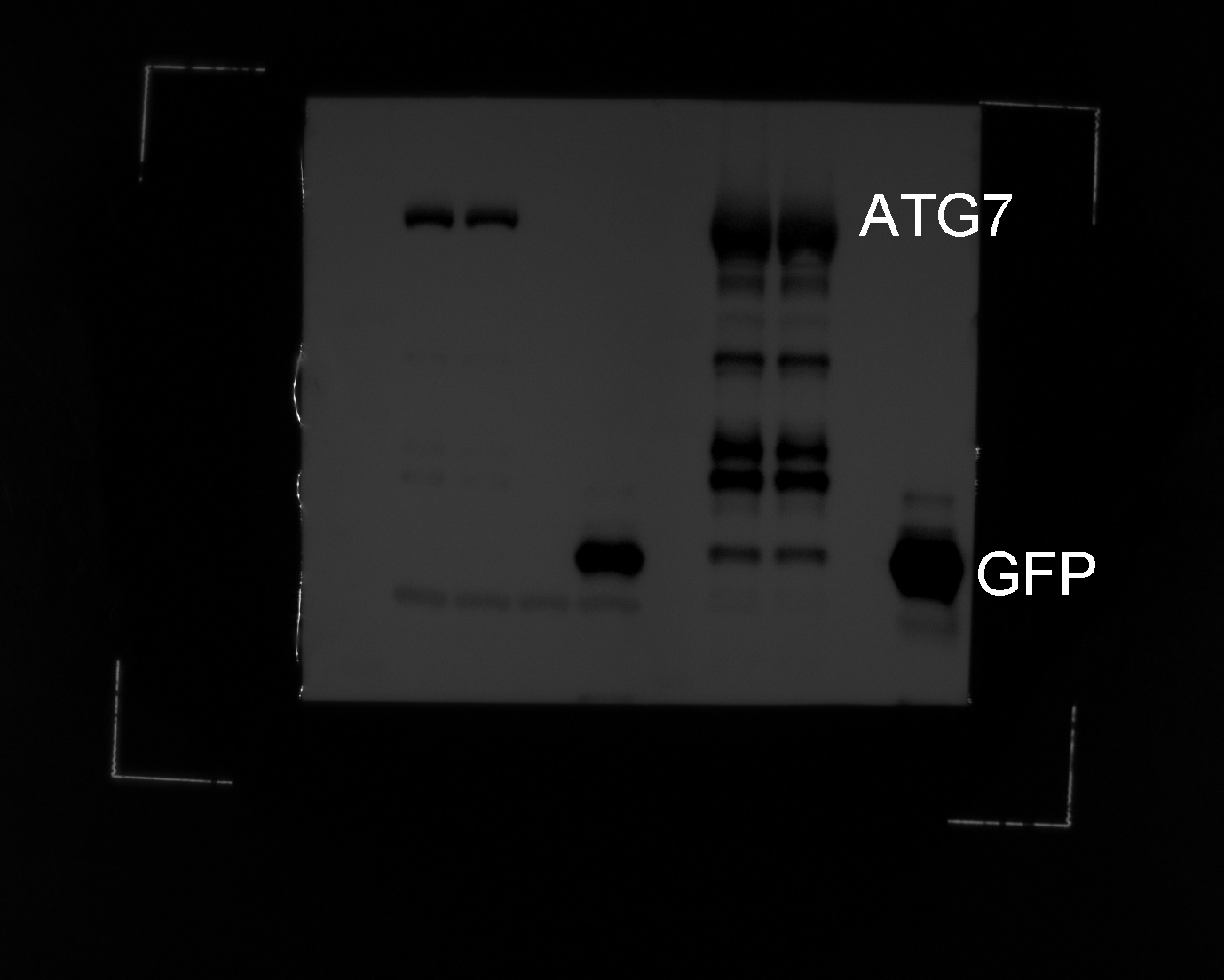

Supplement: Figure 6—source data 2. [file elife-69047-fig6-data2.zip › Figure 6-source data 2. Original western blot files for Figure 6/Figure 6-source data A4.jpg]

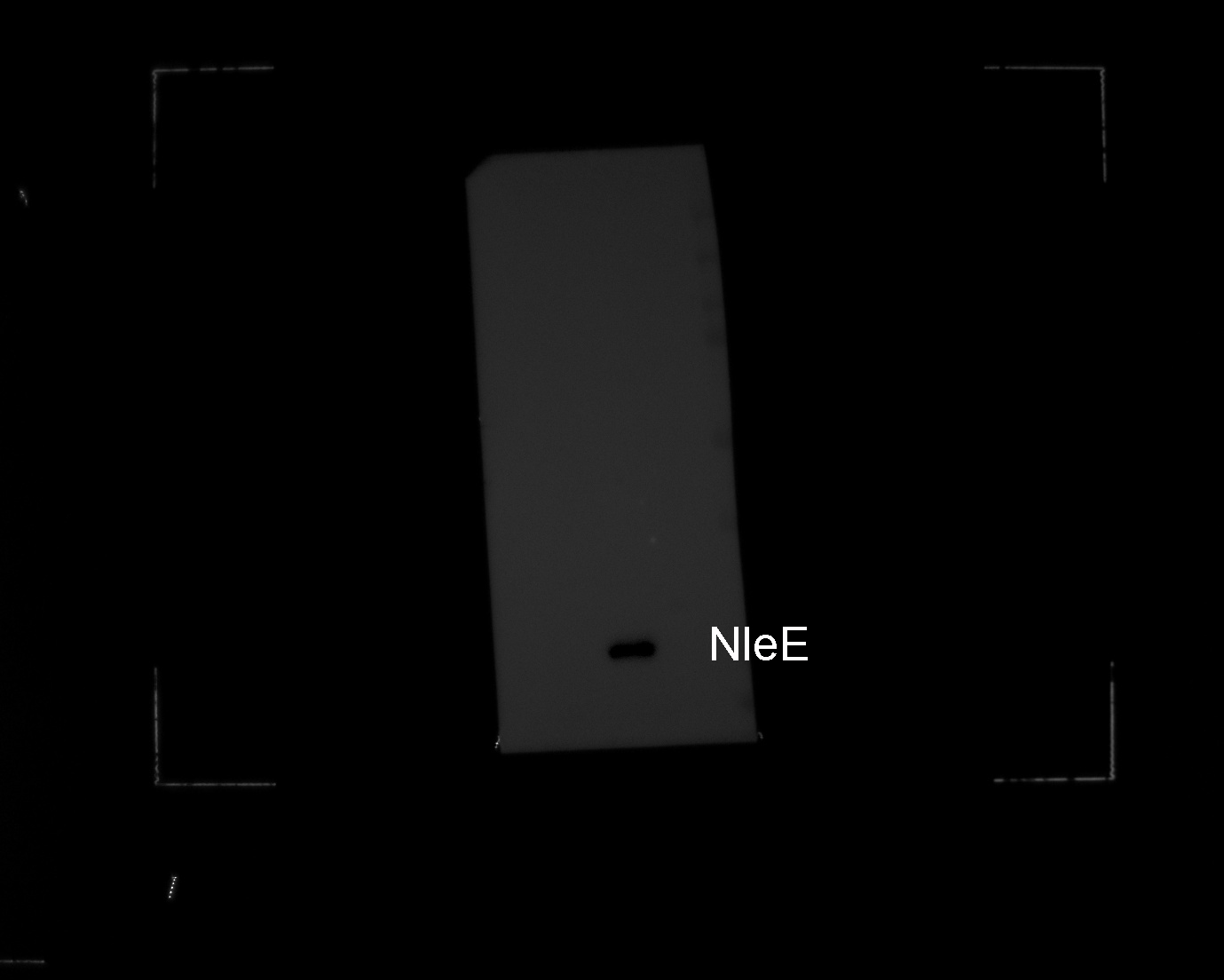

Supplement: Figure 6—source data 2. [file elife-69047-fig6-data2.zip › Figure 6-source data 2. Original western blot files for Figure 6/Figure 6-source data B1.jpg]

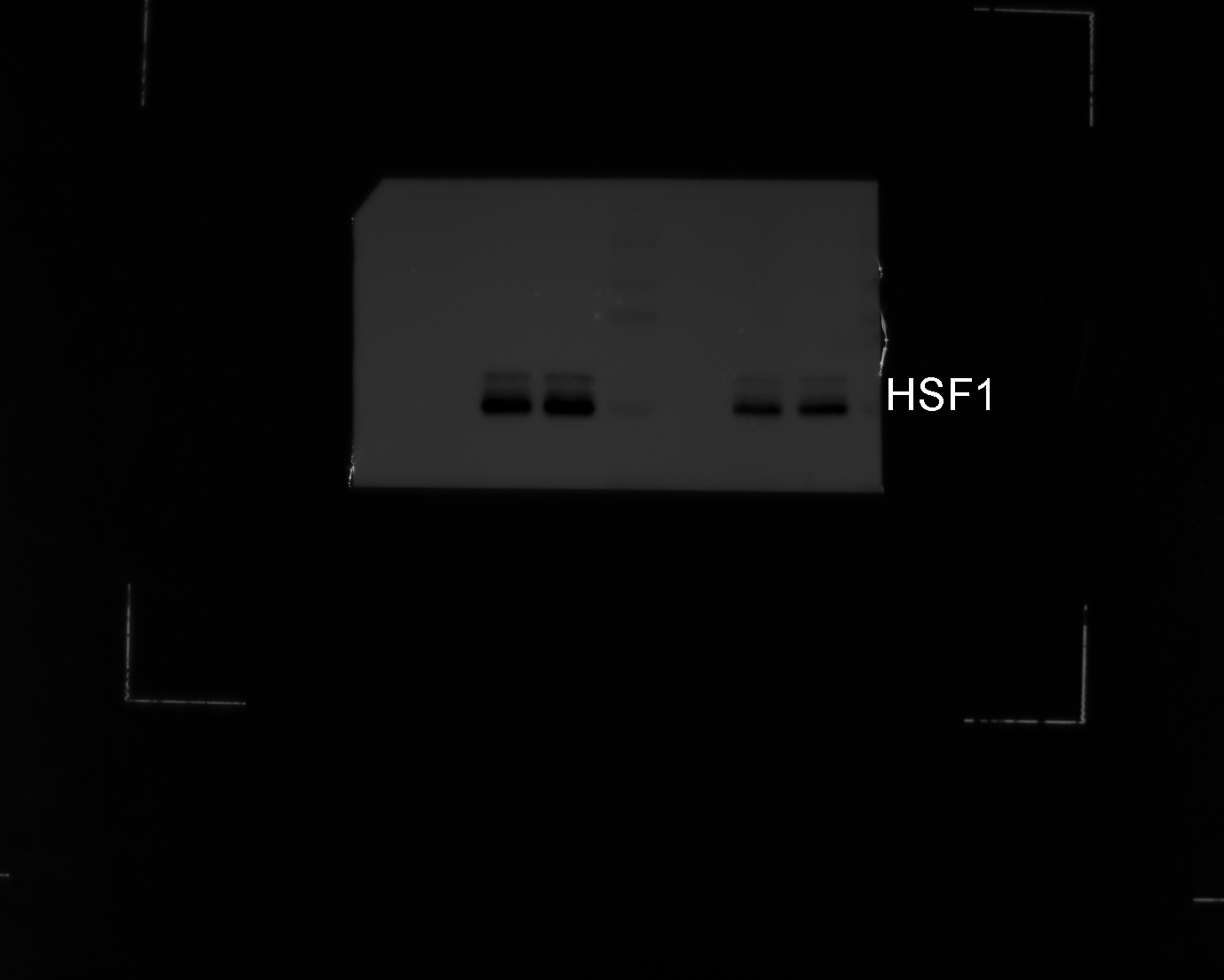

Supplement: Figure 6—source data 2. [file elife-69047-fig6-data2.zip › Figure 6-source data 2. Original western blot files for Figure 6/Figure 6-source data B2.jpg]

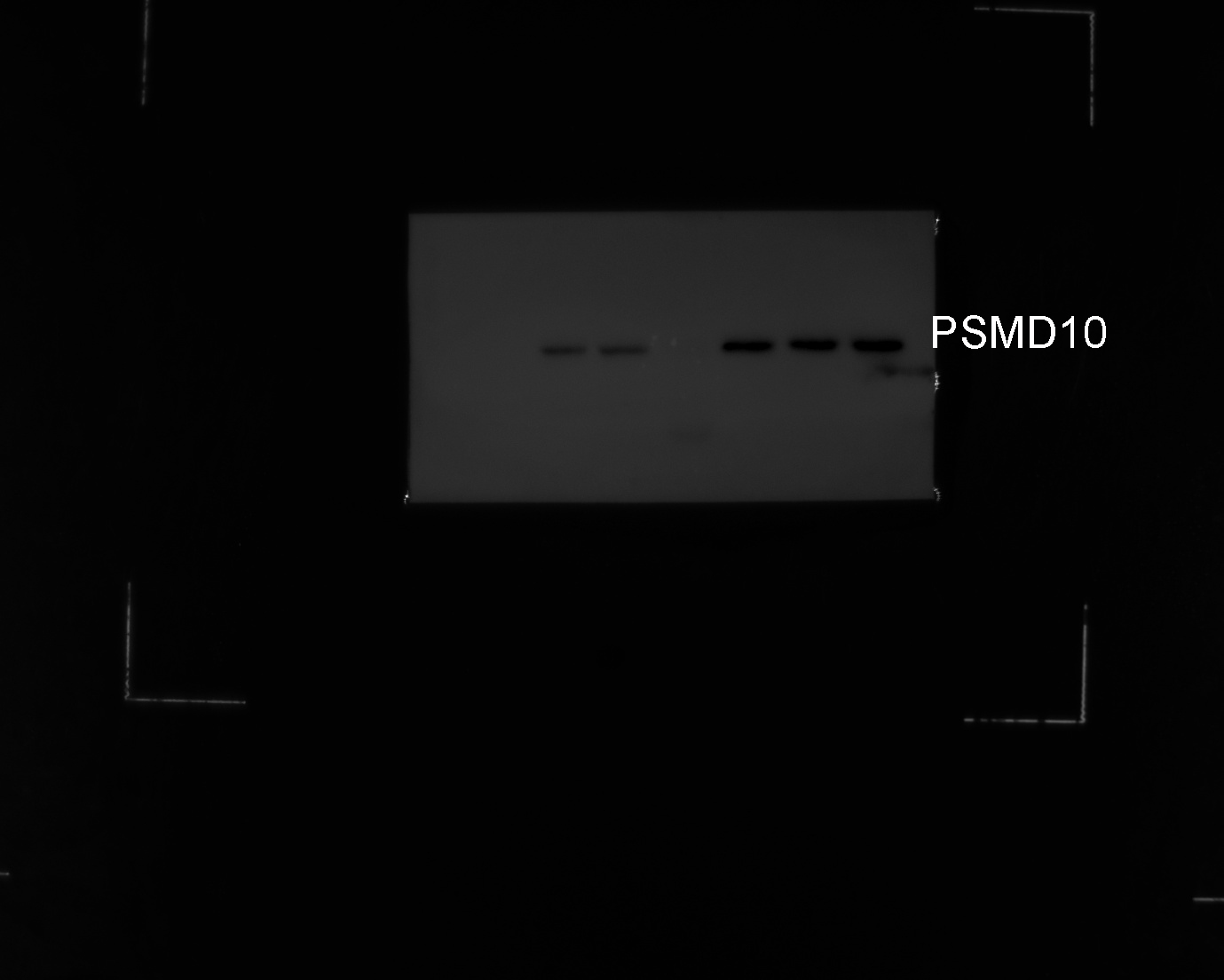

Supplement: Figure 6—source data 2. [file elife-69047-fig6-data2.zip › Figure 6-source data 2. Original western blot files for Figure 6/Figure 6-source data B3.jpg]

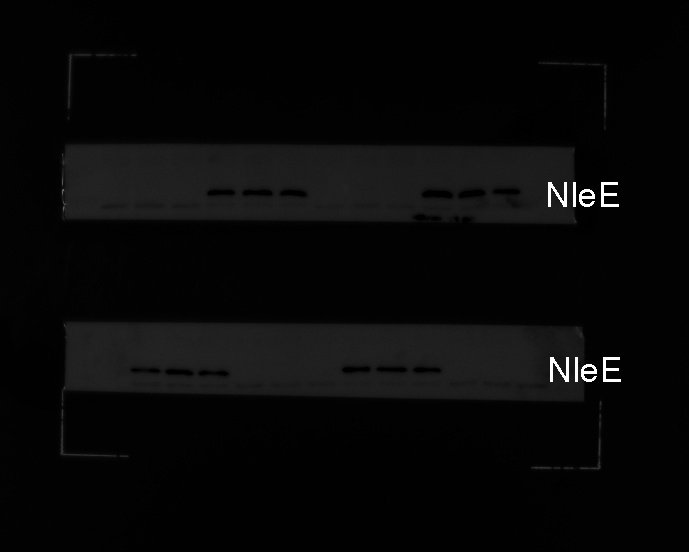

Supplement: Figure 6—source data 2. [file elife-69047-fig6-data2.zip › Figure 6-source data 2. Original western blot files for Figure 6/Figure 6-source data D1.jpg]

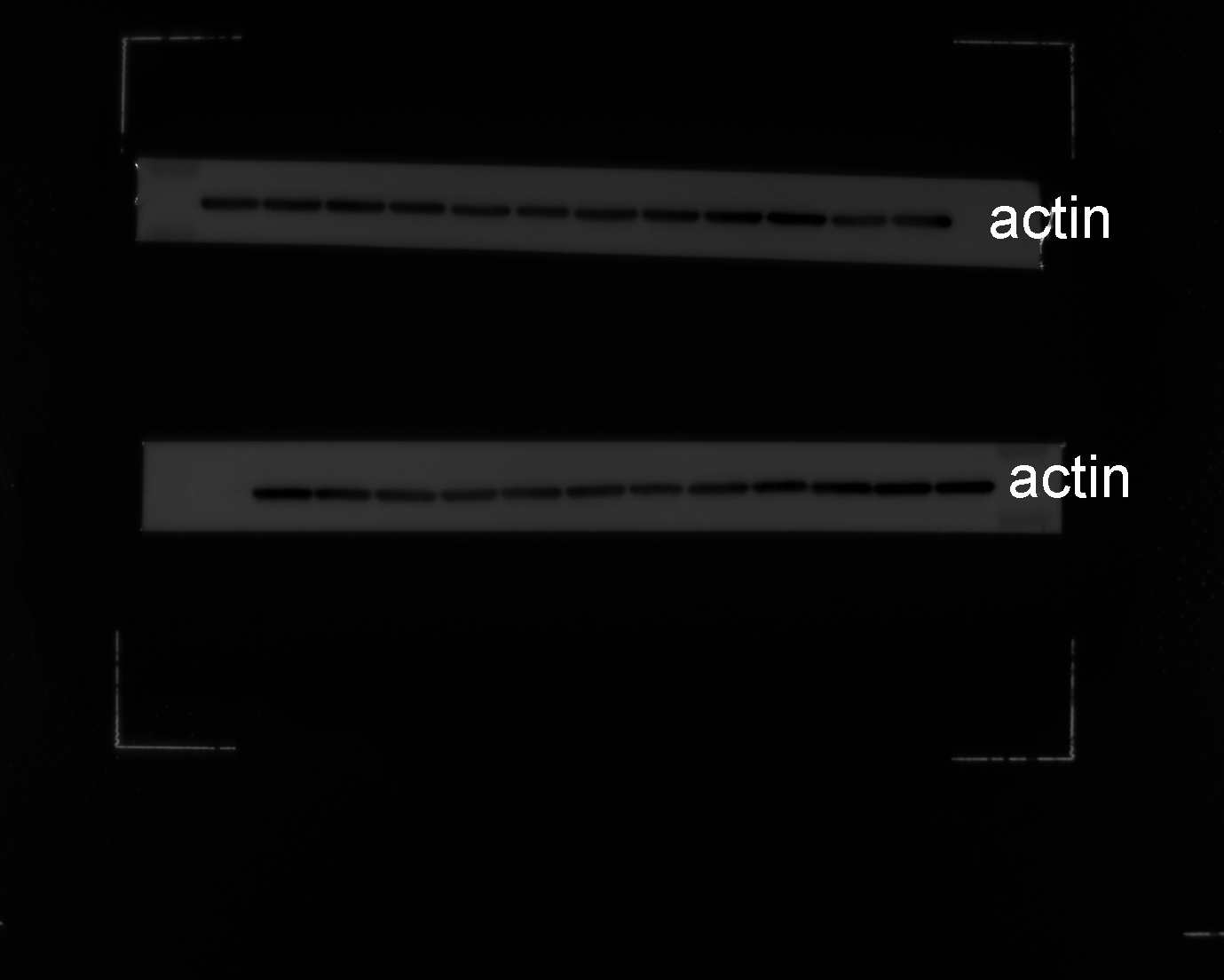

Supplement: Figure 6—source data 2. [file elife-69047-fig6-data2.zip › Figure 6-source data 2. Original western blot files for Figure 6/Figure 6-source data D2.jpg]

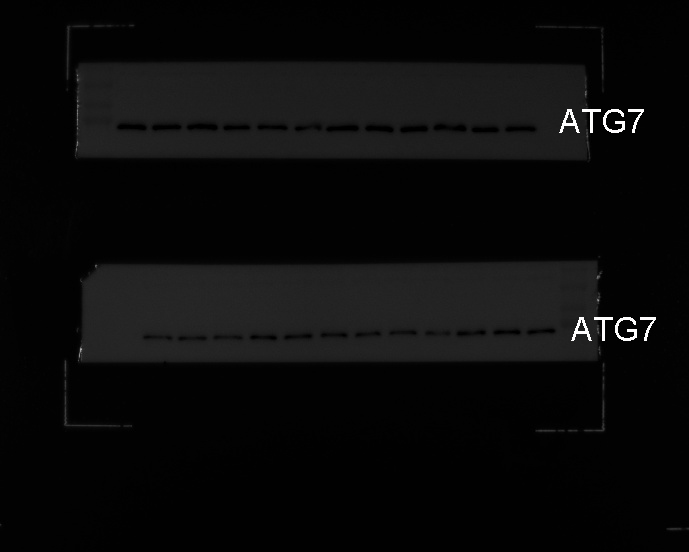

Supplement: Figure 6—source data 2. [file elife-69047-fig6-data2.zip › Figure 6-source data 2. Original western blot files for Figure 6/Figure 6-source data D3.jpg]

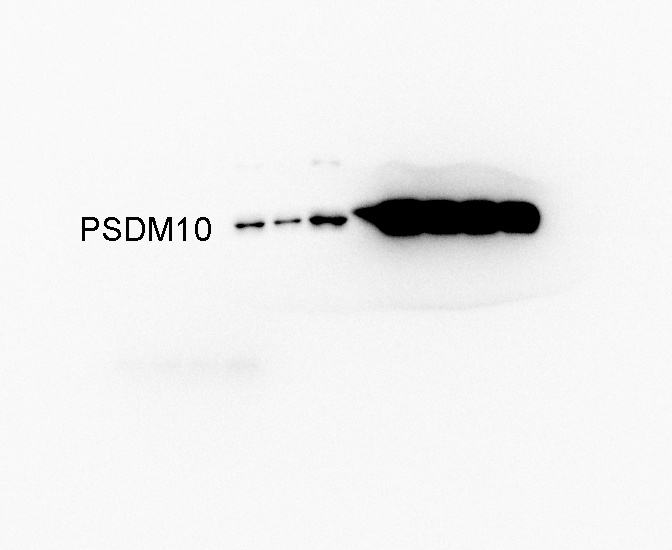

Supplement: Figure 6—source data 2. [file elife-69047-fig6-data2.zip › Figure 6-source data 2. Original western blot files for Figure 6/Figure 6-source data F1.jpg]

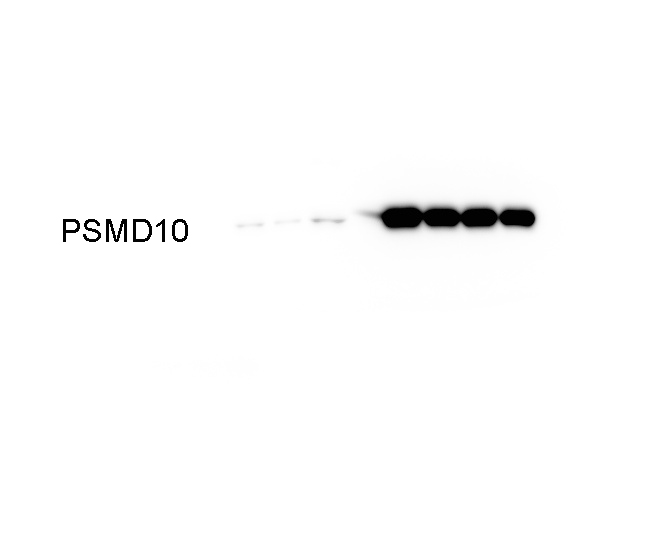

Supplement: Figure 6—source data 2. [file elife-69047-fig6-data2.zip › Figure 6-source data 2. Original western blot files for Figure 6/Figure 6-source data F2.jpg]

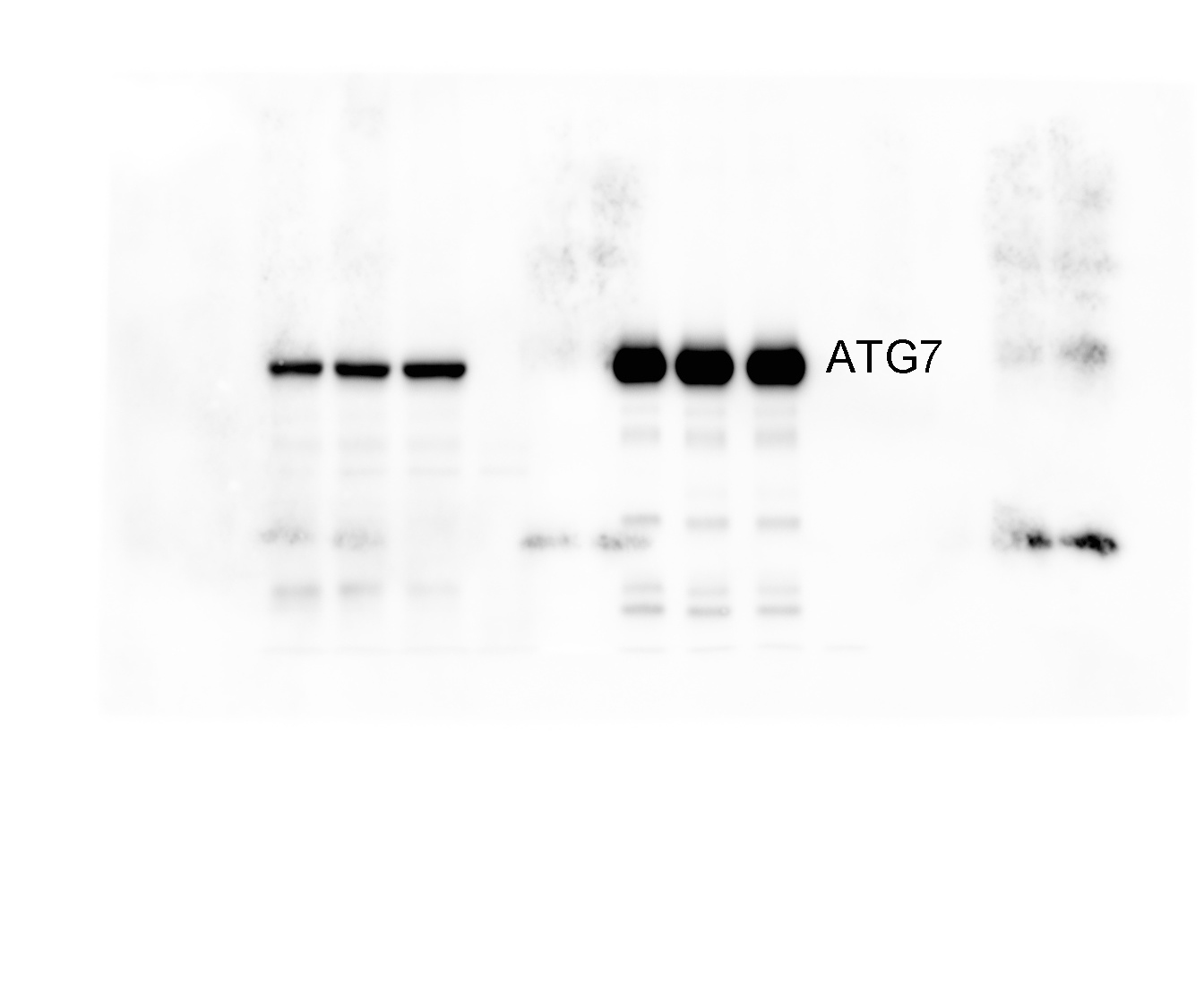

Supplement: Figure 6—source data 2. [file elife-69047-fig6-data2.zip › Figure 6-source data 2. Original western blot files for Figure 6/Figure 6-source data F3.jpg]

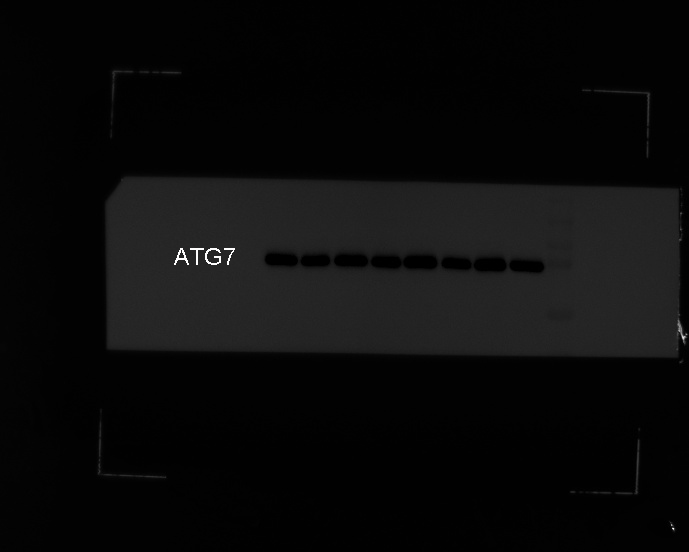

Supplement: Figure 7—source data 1. [file elife-69047-fig7-data1.zip › Figure 7-source data 1. Original western blot files for Figure 7/Figure 7-source data A1.jpg]

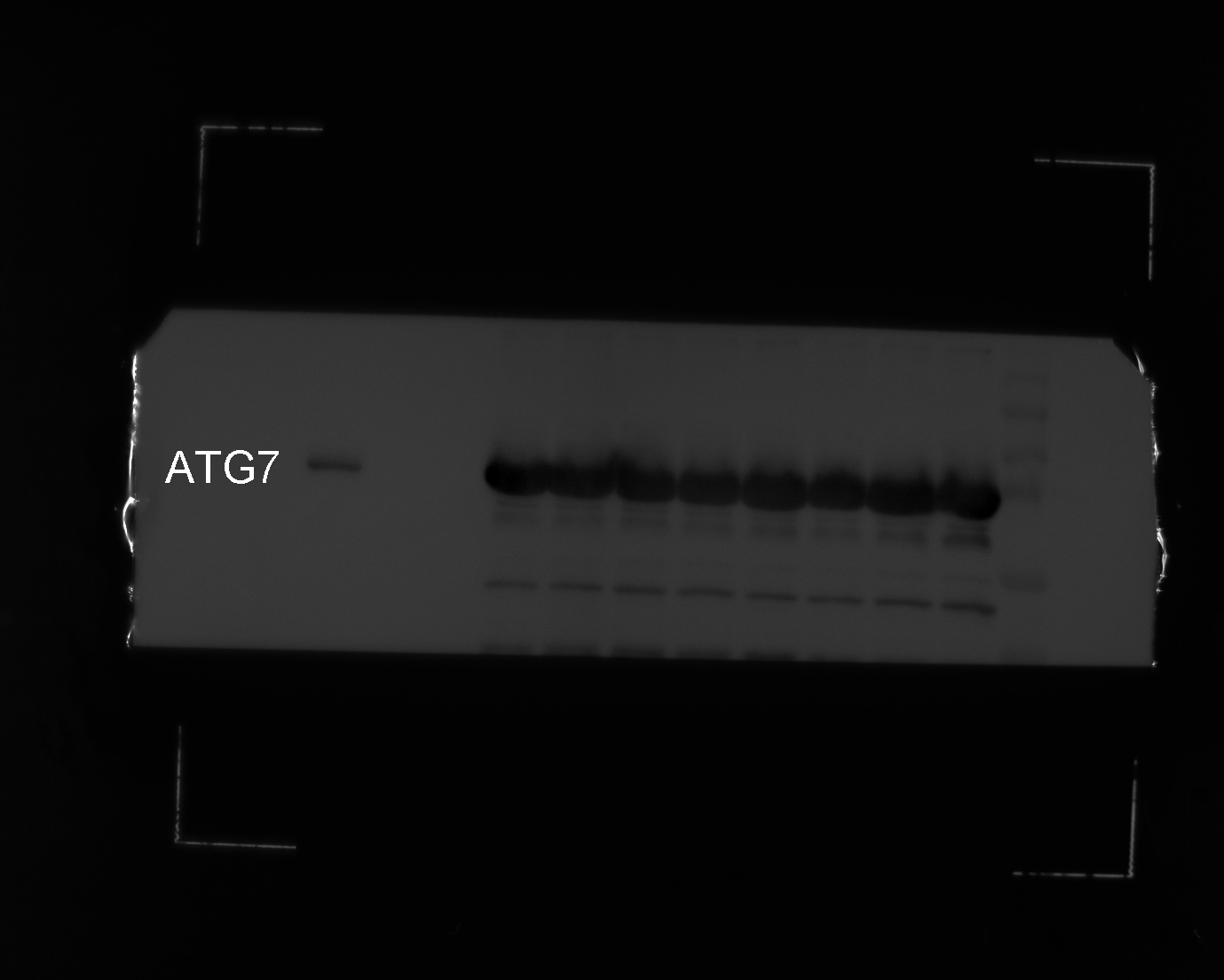

Supplement: Figure 7—source data 1. [file elife-69047-fig7-data1.zip › Figure 7-source data 1. Original western blot files for Figure 7/Figure 7-source data A2.jpg]

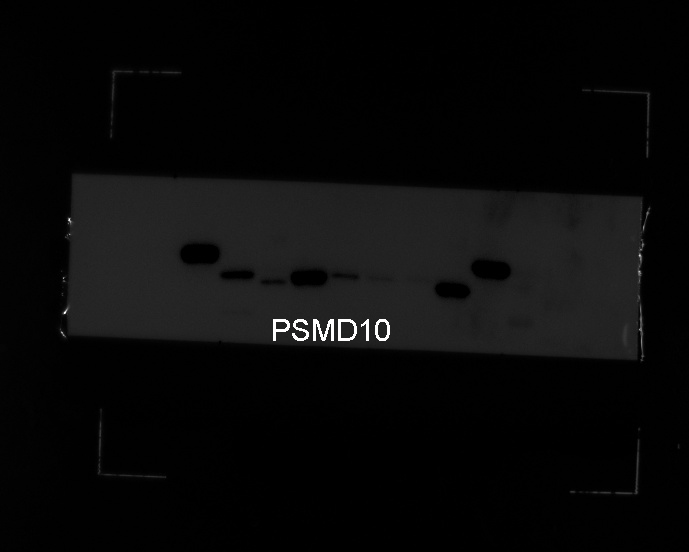

Supplement: Figure 7—source data 1. [file elife-69047-fig7-data1.zip › Figure 7-source data 1. Original western blot files for Figure 7/Figure 7-source data A3.jpg]

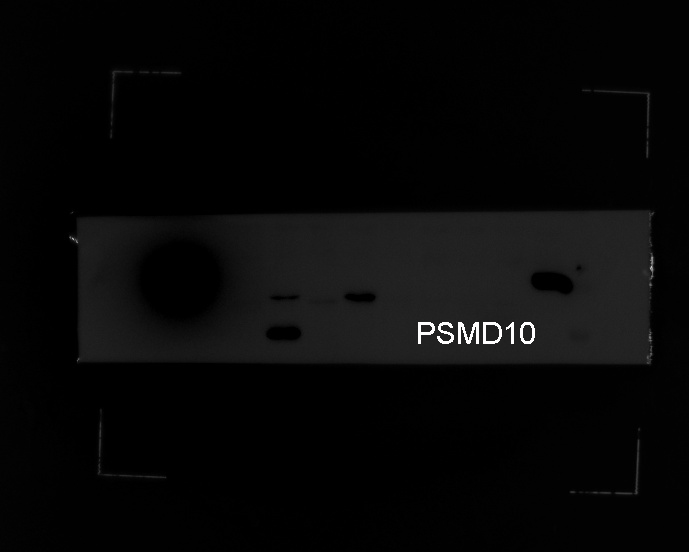

Supplement: Figure 7—source data 1. [file elife-69047-fig7-data1.zip › Figure 7-source data 1. Original western blot files for Figure 7/Figure 7-source data A4.jpg]

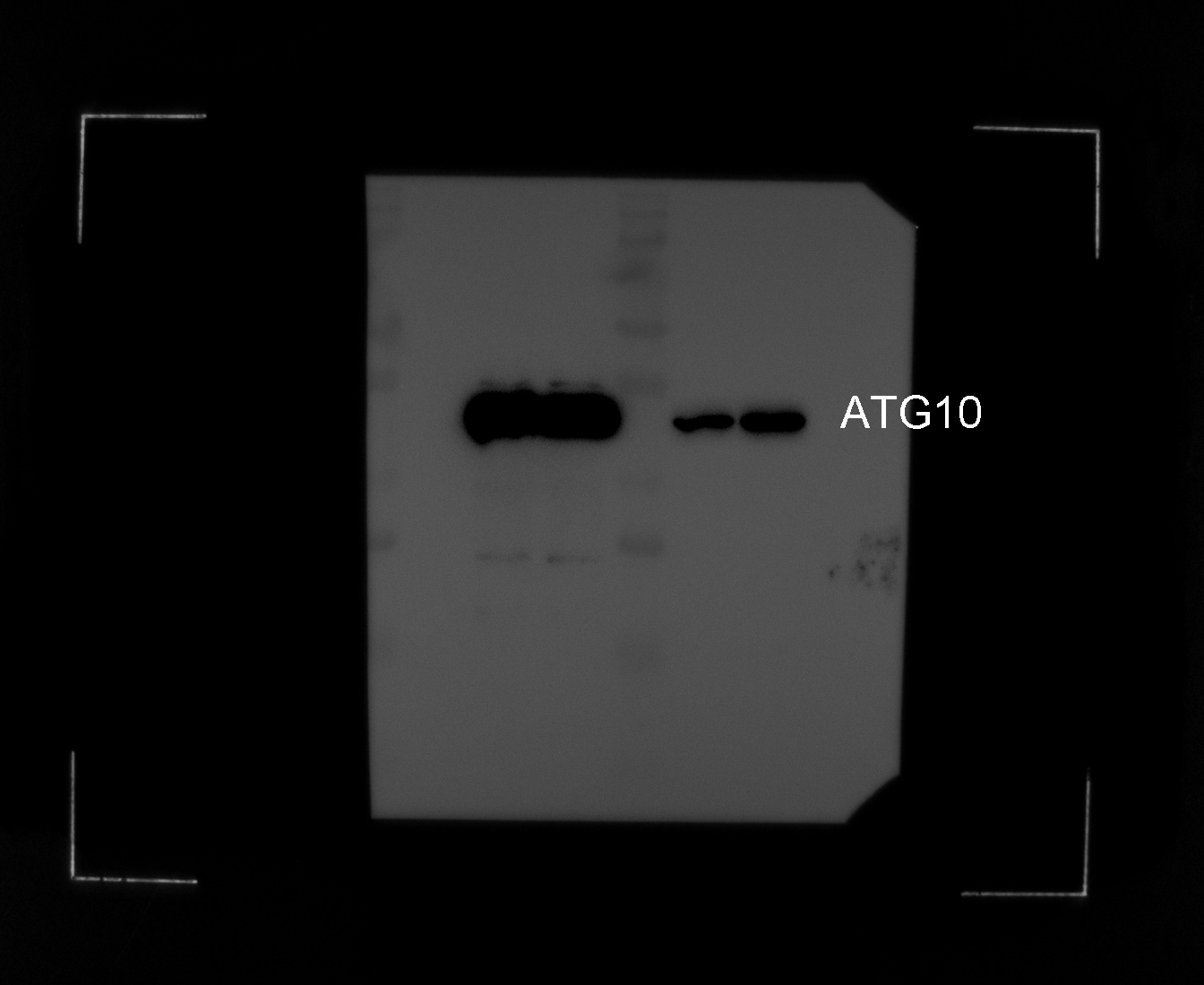

Supplement: Figure 7—source data 1. [file elife-69047-fig7-data1.zip › Figure 7-source data 1. Original western blot files for Figure 7/Figure 7-source data A5.jpg]

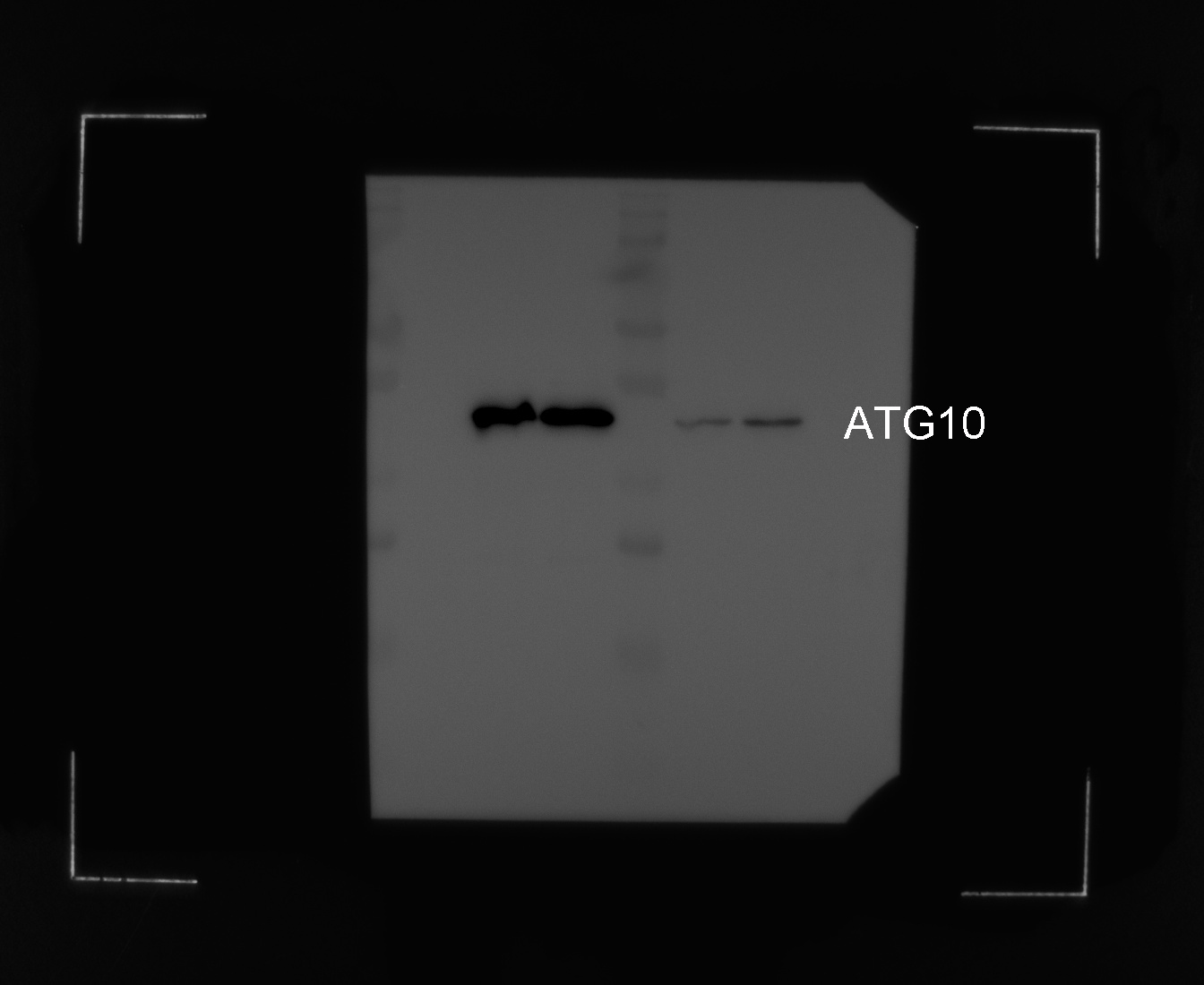

Supplement: Figure 7—source data 1. [file elife-69047-fig7-data1.zip › Figure 7-source data 1. Original western blot files for Figure 7/Figure 7-source data A6.jpg]

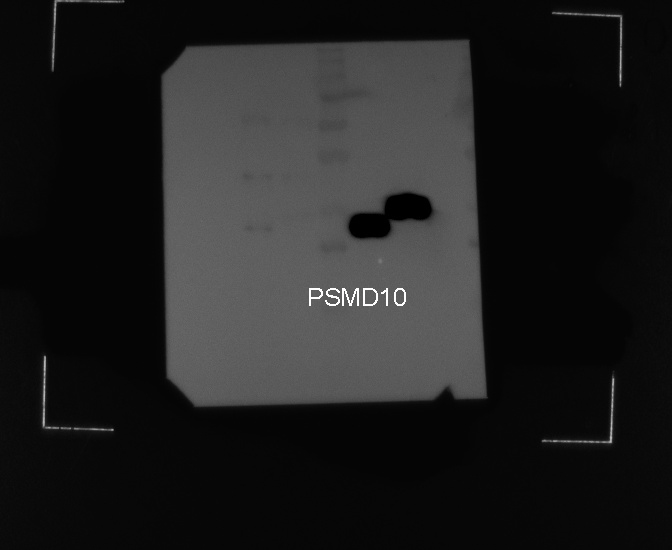

Supplement: Figure 7—source data 1. [file elife-69047-fig7-data1.zip › Figure 7-source data 1. Original western blot files for Figure 7/Figure 7-source data A7.jpg]

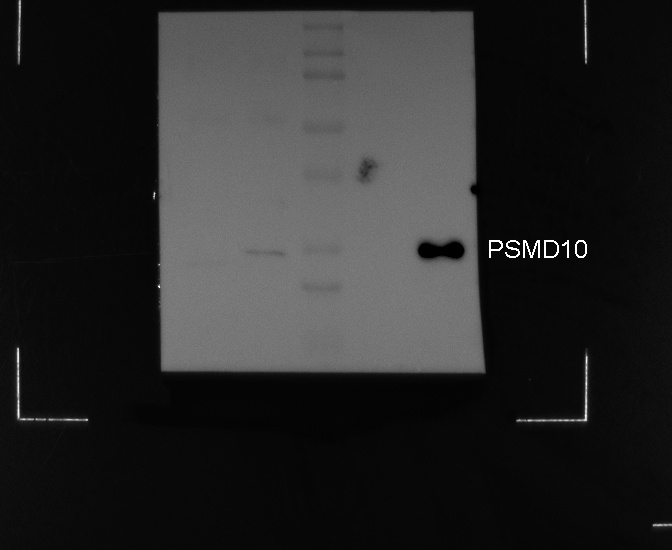

Supplement: Figure 7—source data 1. [file elife-69047-fig7-data1.zip › Figure 7-source data 1. Original western blot files for Figure 7/Figure 7-source data A8.jpg]

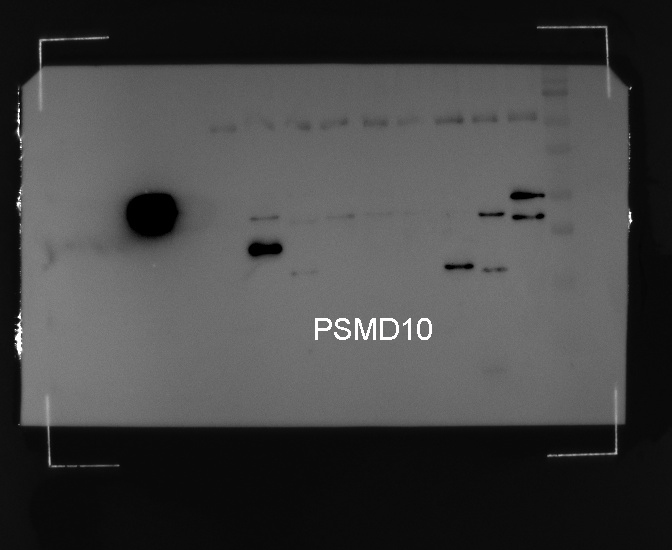

Supplement: Figure 7—source data 1. [file elife-69047-fig7-data1.zip › Figure 7-source data 1. Original western blot files for Figure 7/Figure 7-source data B1.jpg]

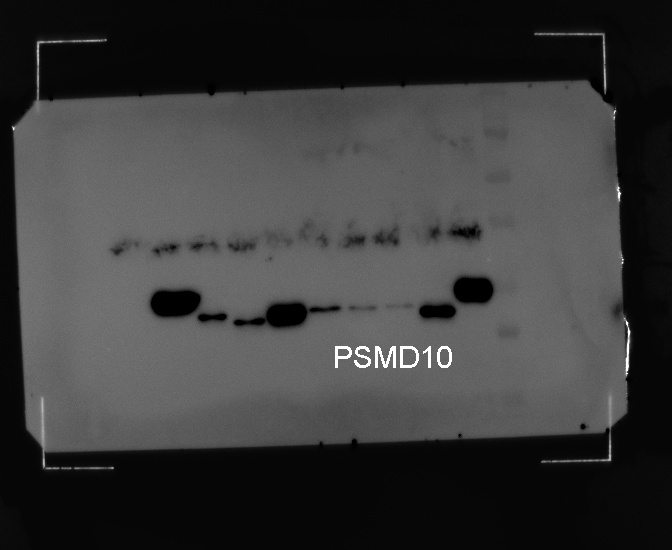

Supplement: Figure 7—source data 1. [file elife-69047-fig7-data1.zip › Figure 7-source data 1. Original western blot files for Figure 7/Figure 7-source data B2.jpg]

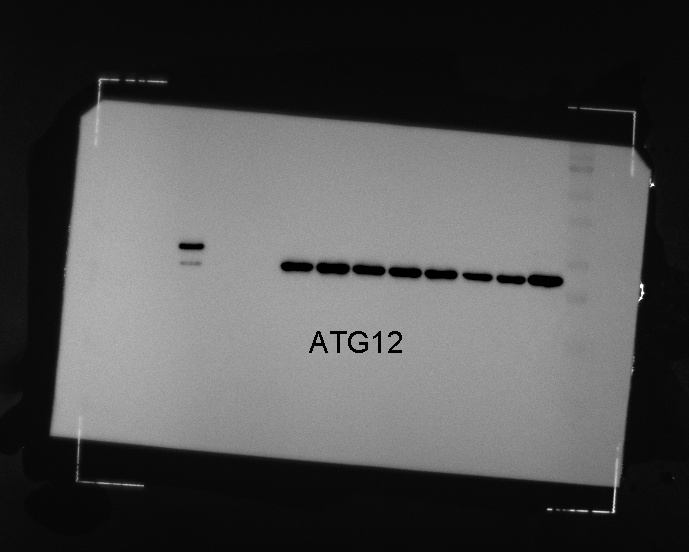

Supplement: Figure 7—source data 1. [file elife-69047-fig7-data1.zip › Figure 7-source data 1. Original western blot files for Figure 7/Figure 7-source data B3.jpg]

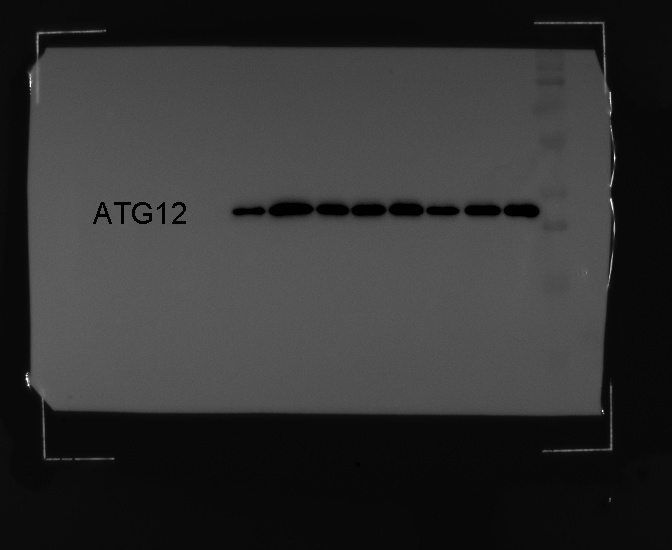

Supplement: Figure 7—source data 1. [file elife-69047-fig7-data1.zip › Figure 7-source data 1. Original western blot files for Figure 7/Figure 7-source data B4.jpg]

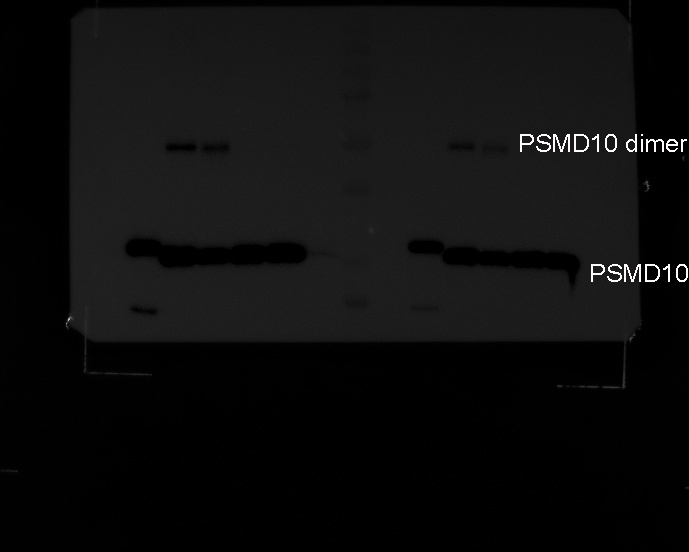

Supplement: Figure 7—source data 1. [file elife-69047-fig7-data1.zip › Figure 7-source data 1. Original western blot files for Figure 7/Figure 7-source data E1.jpg]

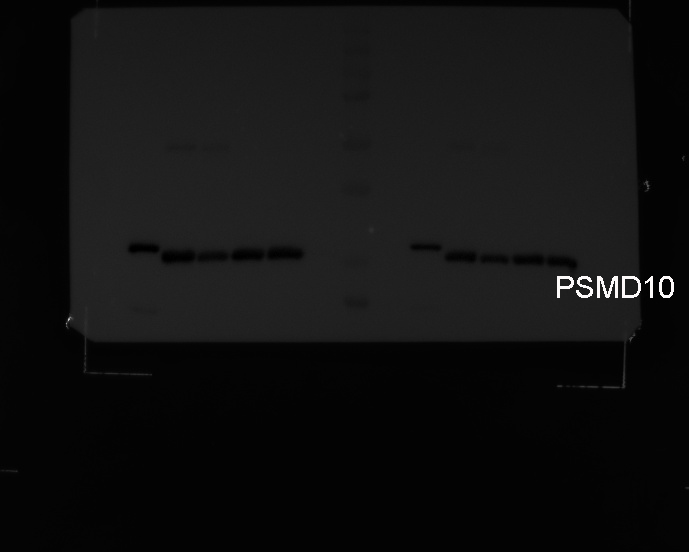

Supplement: Figure 7—source data 1. [file elife-69047-fig7-data1.zip › Figure 7-source data 1. Original western blot files for Figure 7/Figure 7-source data E2.jpg]

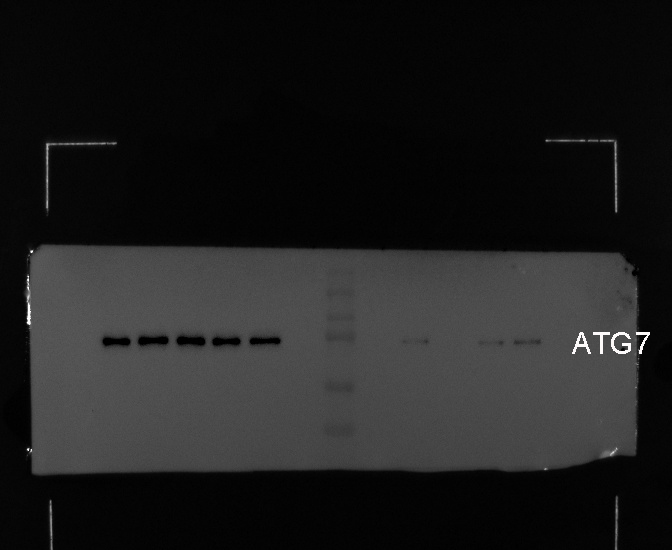

Supplement: Figure 7—source data 1. [file elife-69047-fig7-data1.zip › Figure 7-source data 1. Original western blot files for Figure 7/Figure 7-source data E3.jpg]

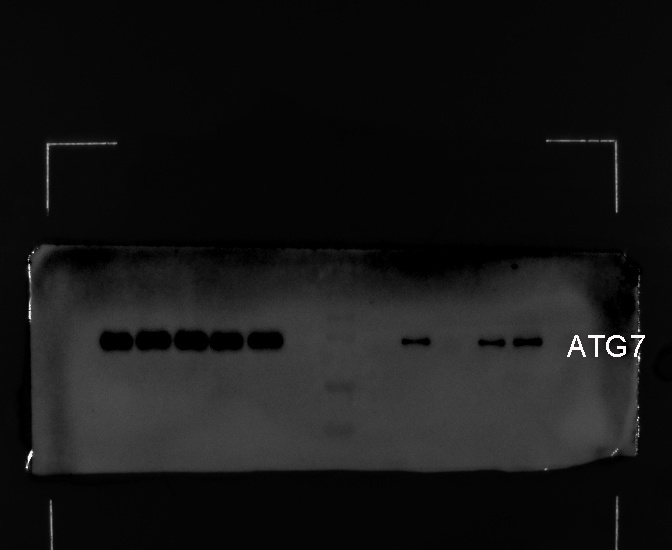

Supplement: Figure 7—source data 1. [file elife-69047-fig7-data1.zip › Figure 7-source data 1. Original western blot files for Figure 7/Figure 7-source data E4.jpg]

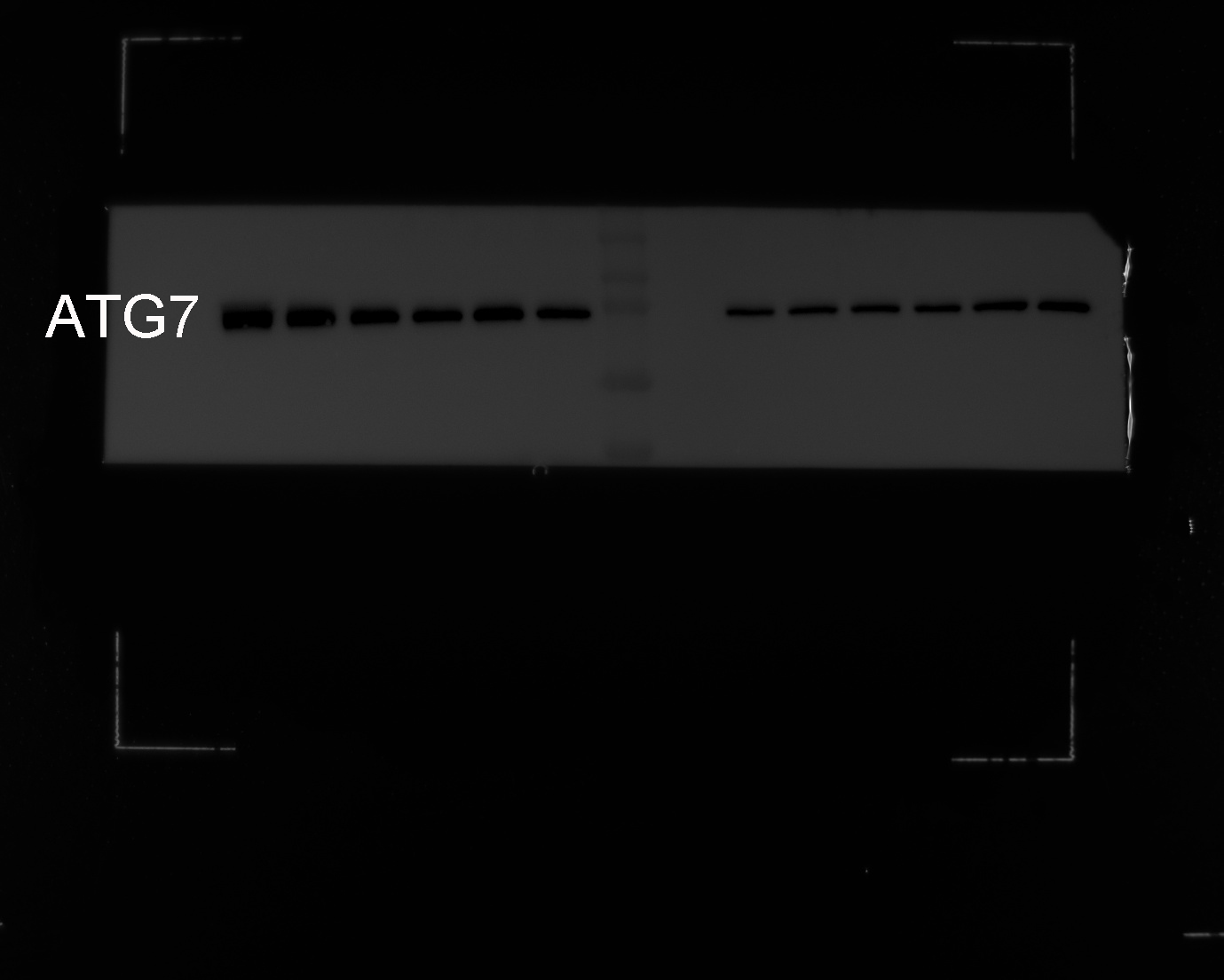

Supplement: Figure 7—source data 1. [file elife-69047-fig7-data1.zip › Figure 7-source data 1. Original western blot files for Figure 7/Figure 7-source data F1.jpg]

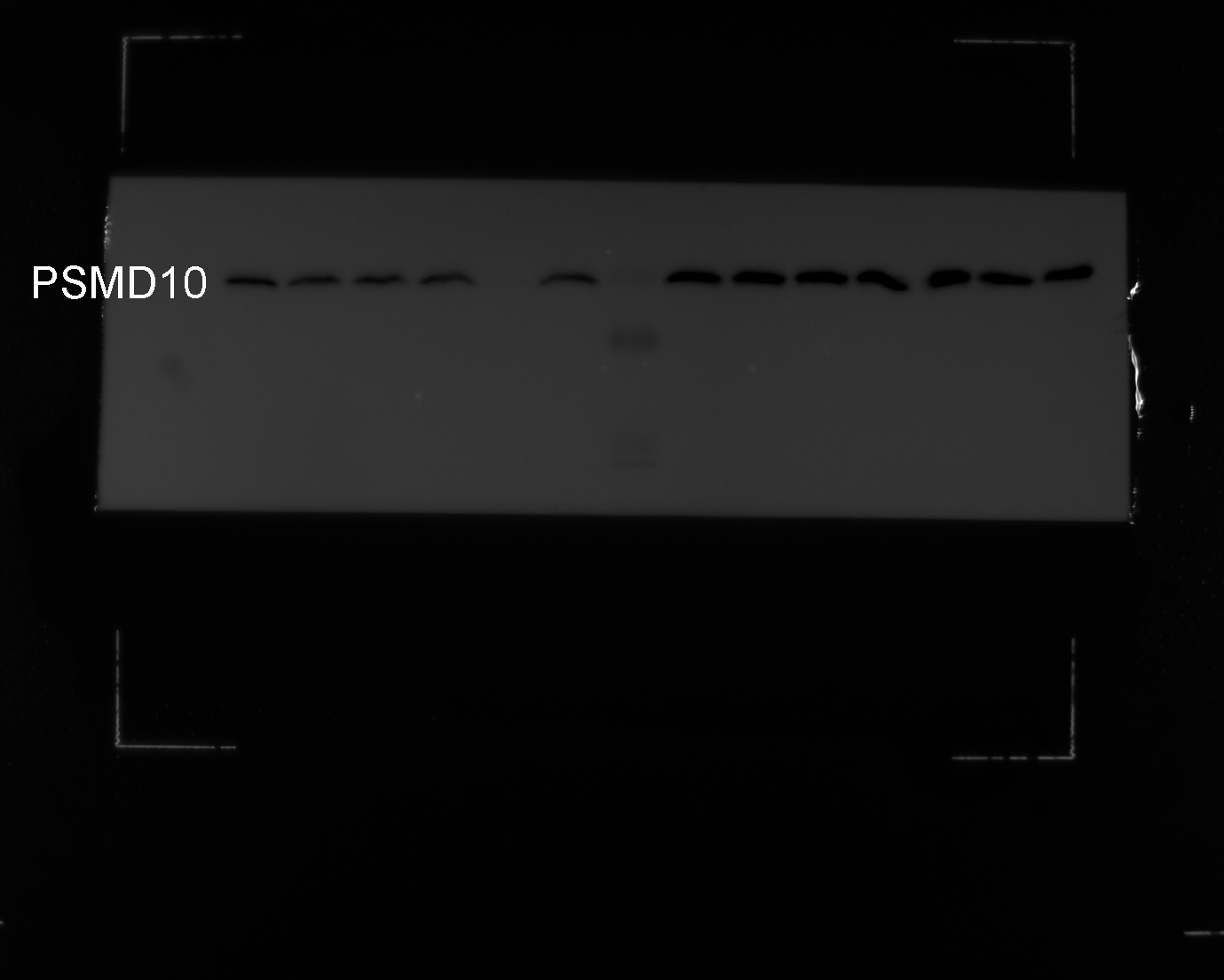

Supplement: Figure 7—source data 1. [file elife-69047-fig7-data1.zip › Figure 7-source data 1. Original western blot files for Figure 7/Figure 7-source data F2.jpg]

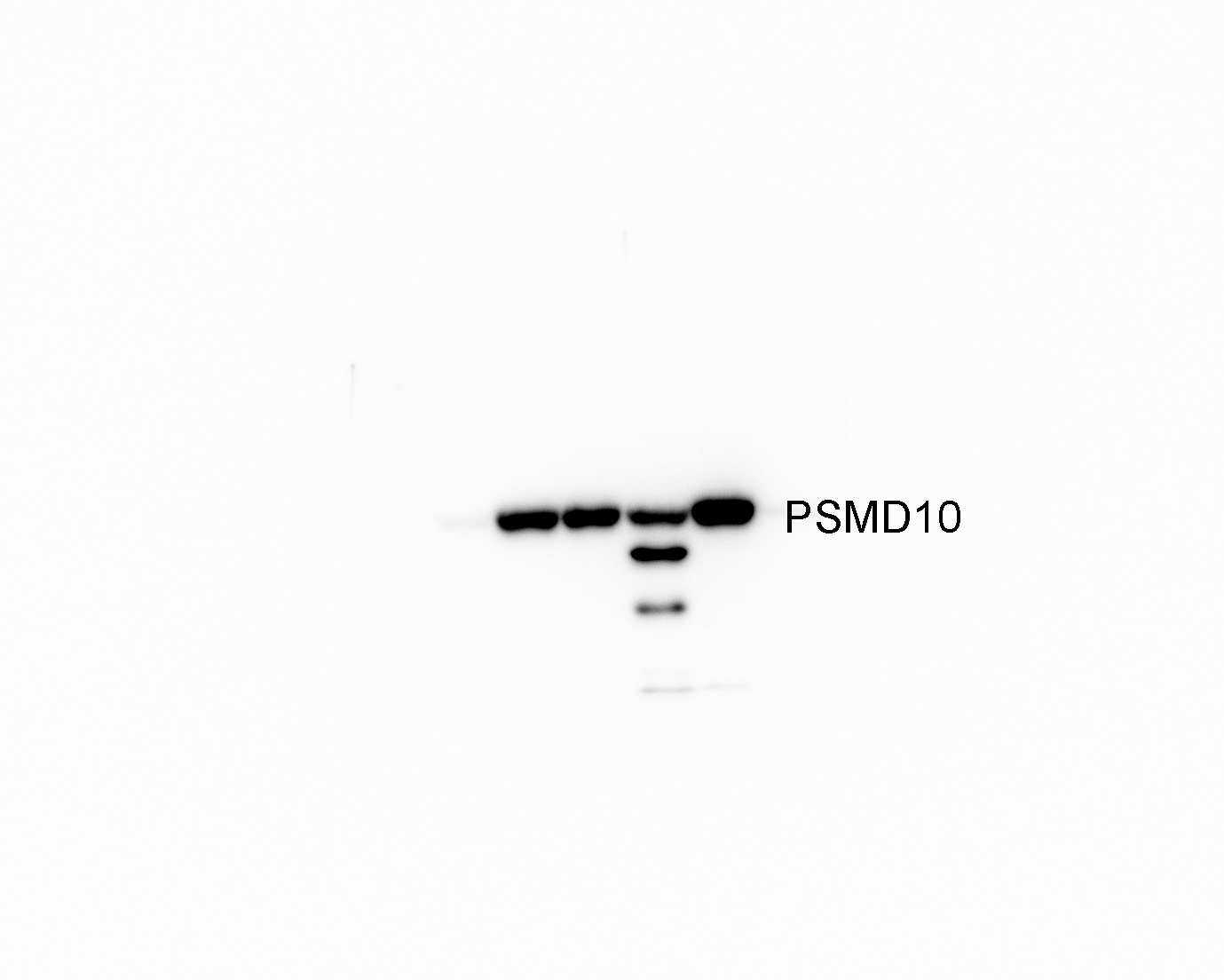

Supplement: Figure 7—figure supplement 1—source data 1. [file elife-69047-fig7-figsupp1-data1.zip › Figure 7-figure supplement 1-source data 1. Original western blot files for Figure 7-figure supplement 1/Figure 7-figure supplement 1-source data A1.jpg]

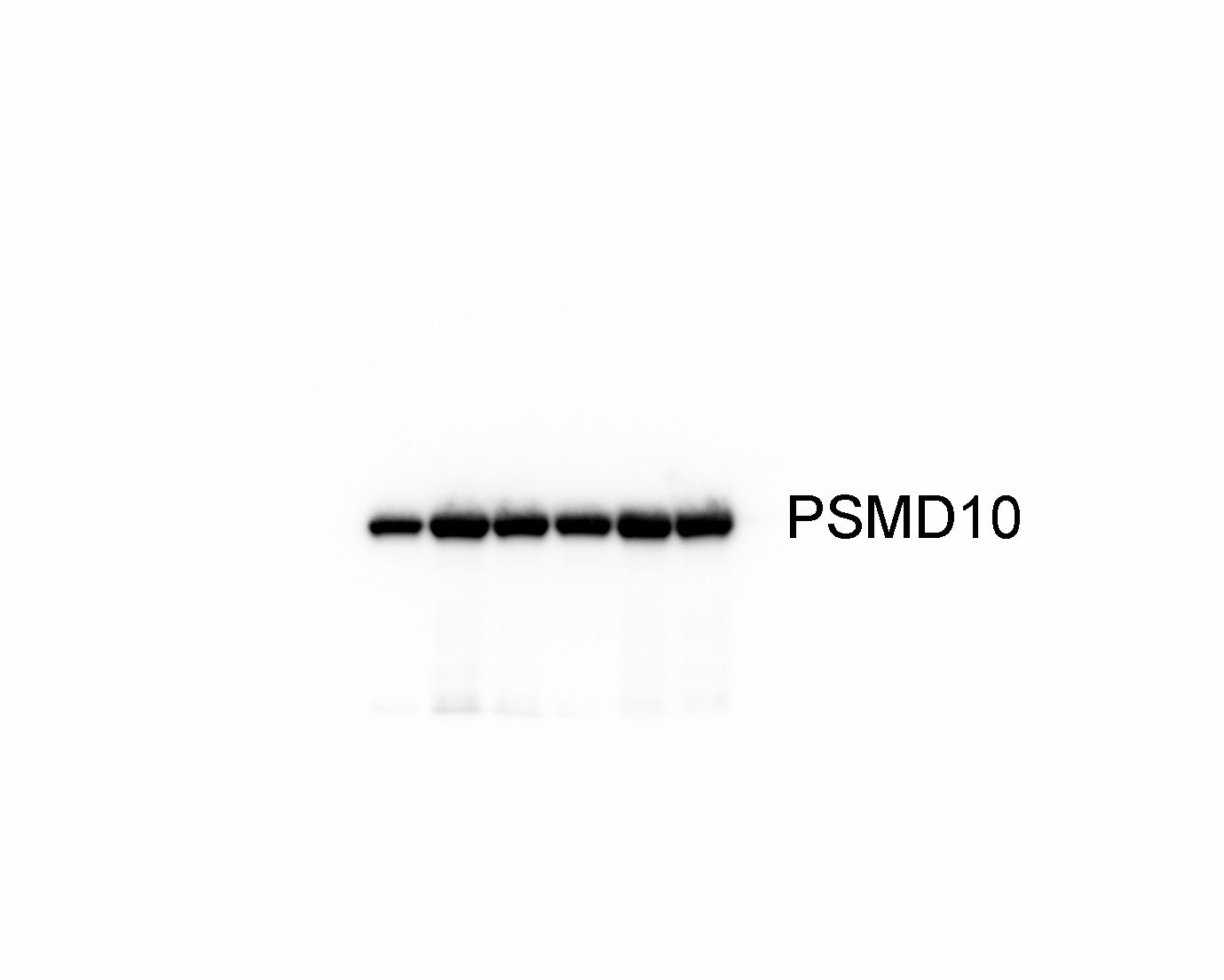

Supplement: Figure 7—figure supplement 1—source data 1. [file elife-69047-fig7-figsupp1-data1.zip › Figure 7-figure supplement 1-source data 1. Original western blot files for Figure 7-figure supplement 1/Figure 7-figure supplement 1-source data A2.jpg]
