# Supplementary figures and images for: Genetically incorporated crosslinkers reveal NleE attenuates host autophagy dependent on PSMD10 (part 2 of 2)
Source: eLife. 2021 Jul 13;10:e69047. doi: 10.7554/eLife.69047 (PMC8324295; doi:10.7554/eLife.69047)

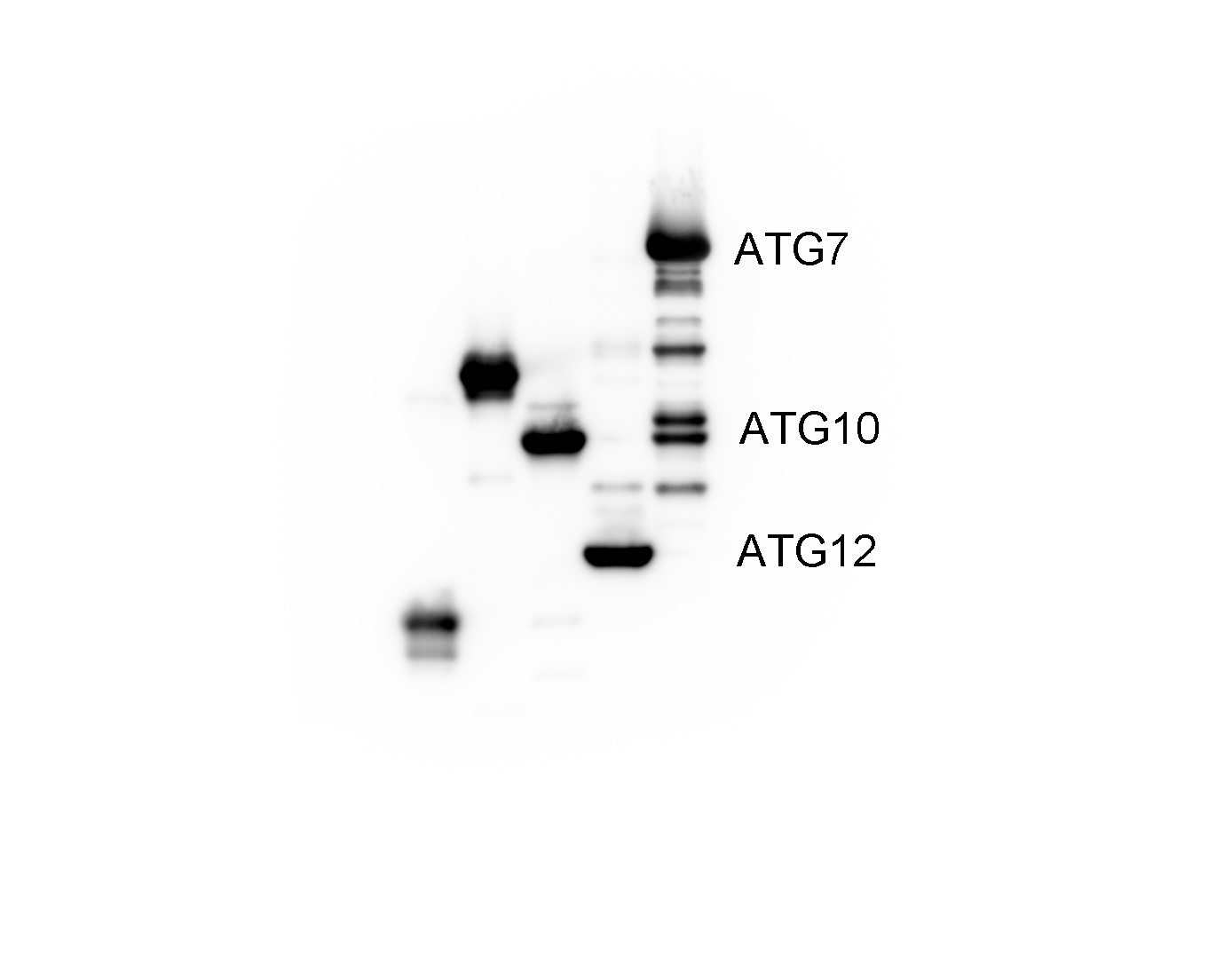

Supplement: Figure 7—figure supplement 1—source data 1. [file elife-69047-fig7-figsupp1-data1.zip › Figure 7-figure supplement 1-source data 1. Original western blot files for Figure 7-figure supplement 1/Figure 7-figure supplement 1-source data A3.jpg]

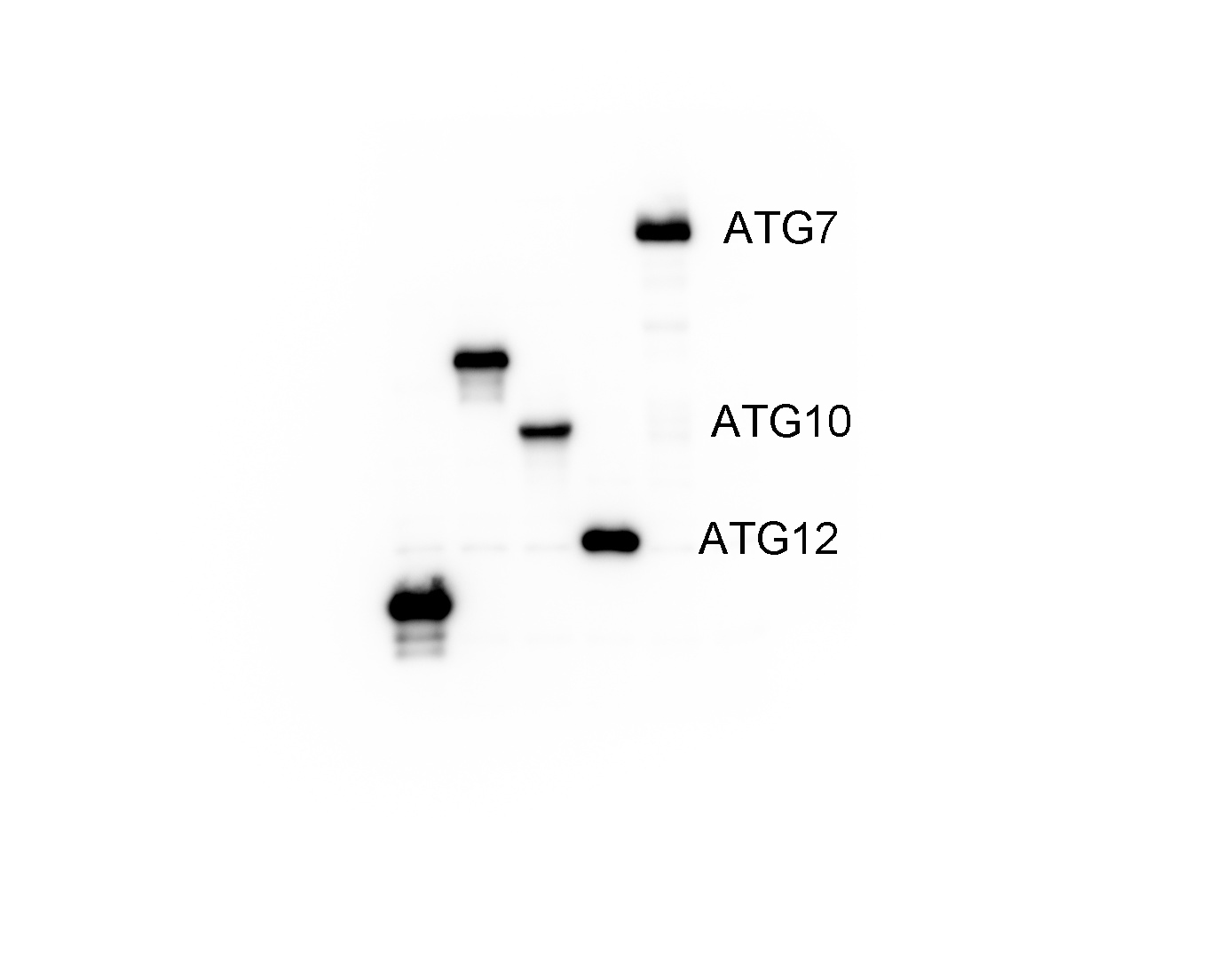

Supplement: Figure 7—figure supplement 1—source data 1. [file elife-69047-fig7-figsupp1-data1.zip › Figure 7-figure supplement 1-source data 1. Original western blot files for Figure 7-figure supplement 1/Figure 7-figure supplement 1-source data A4.jpg]

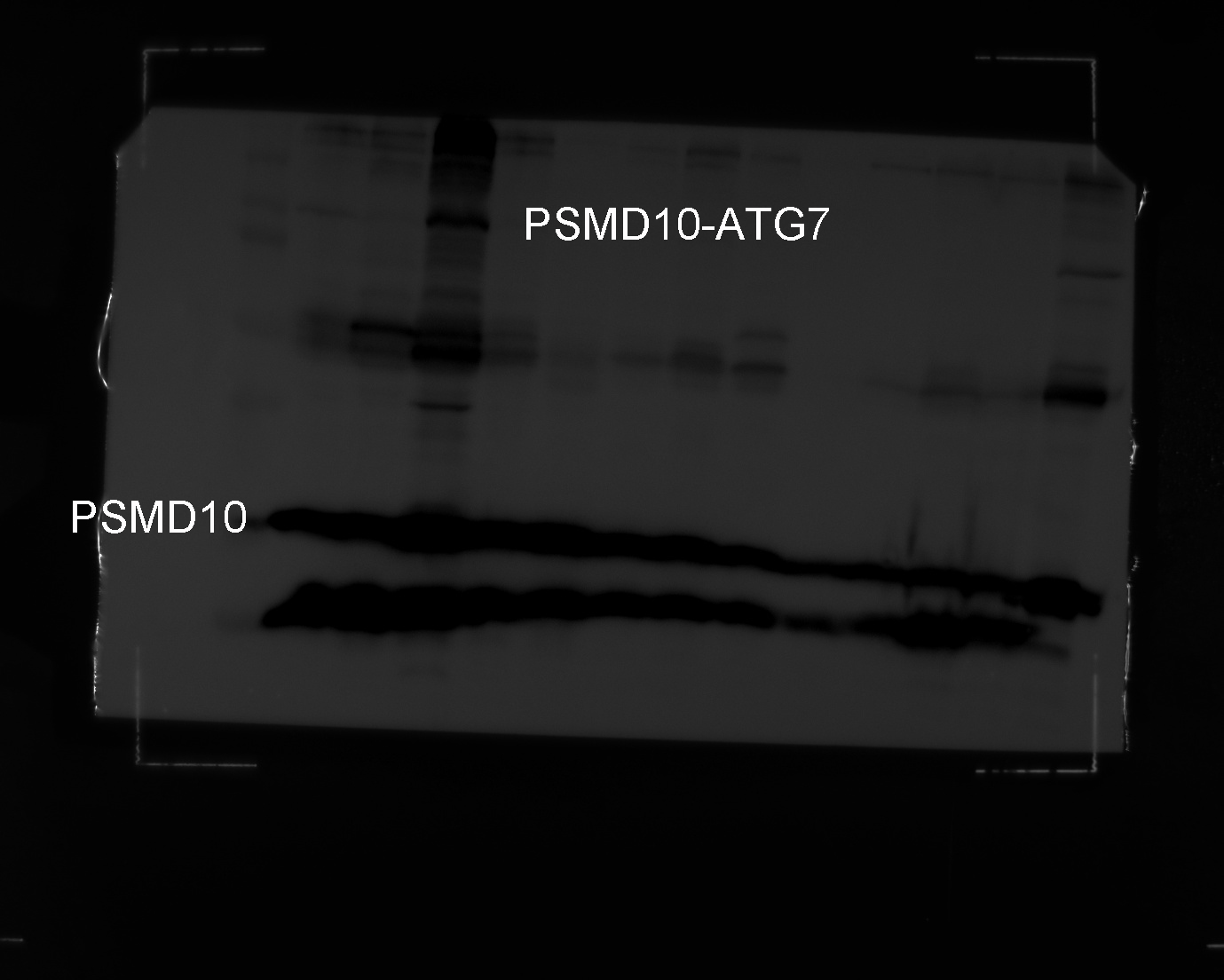

Supplement: Figure 7—figure supplement 1—source data 1. [file elife-69047-fig7-figsupp1-data1.zip › Figure 7-figure supplement 1-source data 1. Original western blot files for Figure 7-figure supplement 1/Figure 7-figure supplement 1-source data B1.jpg]

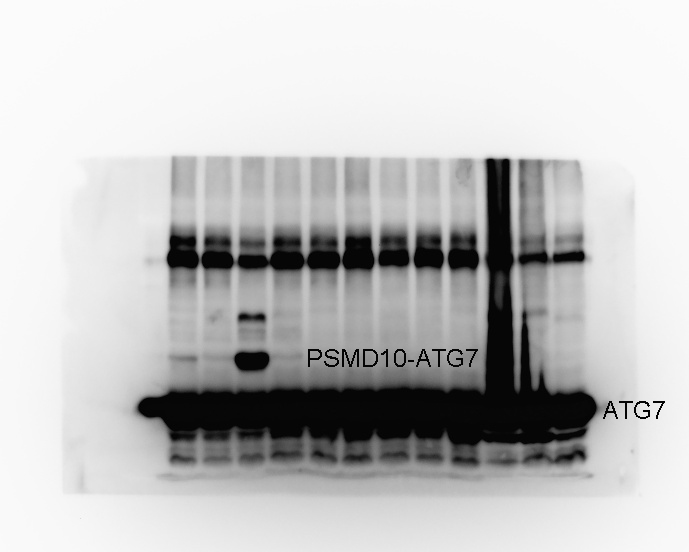

Supplement: Figure 7—figure supplement 1—source data 1. [file elife-69047-fig7-figsupp1-data1.zip › Figure 7-figure supplement 1-source data 1. Original western blot files for Figure 7-figure supplement 1/Figure 7-figure supplement 1-source data B2.jpg]

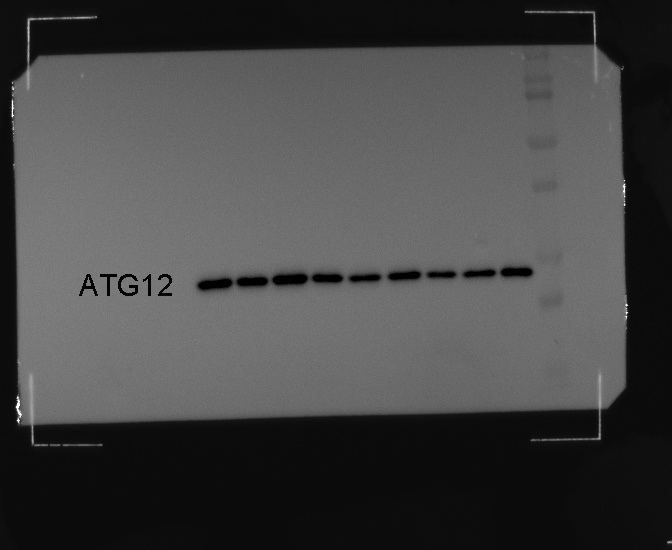

Supplement: Figure 7—figure supplement 1—source data 1. [file elife-69047-fig7-figsupp1-data1.zip › Figure 7-figure supplement 1-source data 1. Original western blot files for Figure 7-figure supplement 1/Figure 7-figure supplement 1-source data D1.jpg]

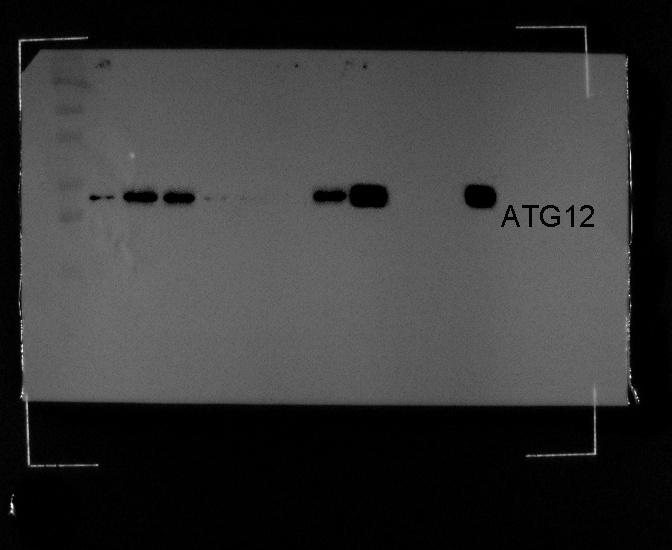

Supplement: Figure 7—figure supplement 1—source data 1. [file elife-69047-fig7-figsupp1-data1.zip › Figure 7-figure supplement 1-source data 1. Original western blot files for Figure 7-figure supplement 1/Figure 7-figure supplement 1-source data D2.jpg]

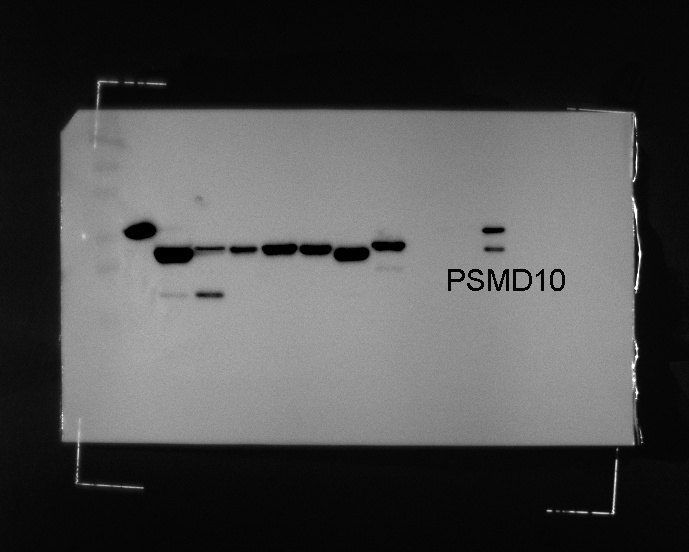

Supplement: Figure 7—figure supplement 1—source data 1. [file elife-69047-fig7-figsupp1-data1.zip › Figure 7-figure supplement 1-source data 1. Original western blot files for Figure 7-figure supplement 1/Figure 7-figure supplement 1-source data D3.jpg]

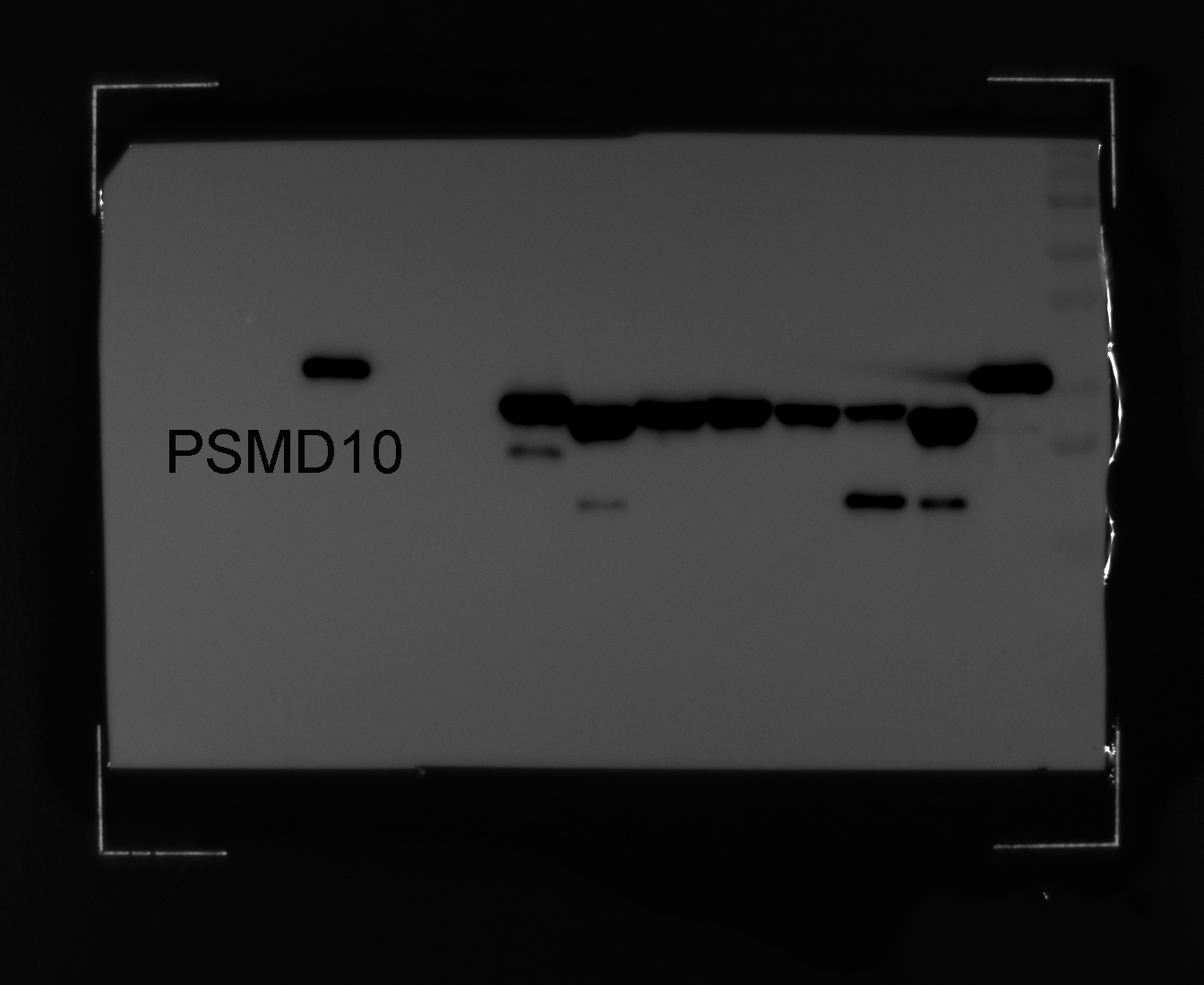

Supplement: Figure 7—figure supplement 1—source data 1. [file elife-69047-fig7-figsupp1-data1.zip › Figure 7-figure supplement 1-source data 1. Original western blot files for Figure 7-figure supplement 1/Figure 7-figure supplement 1-source data D4.jpg]

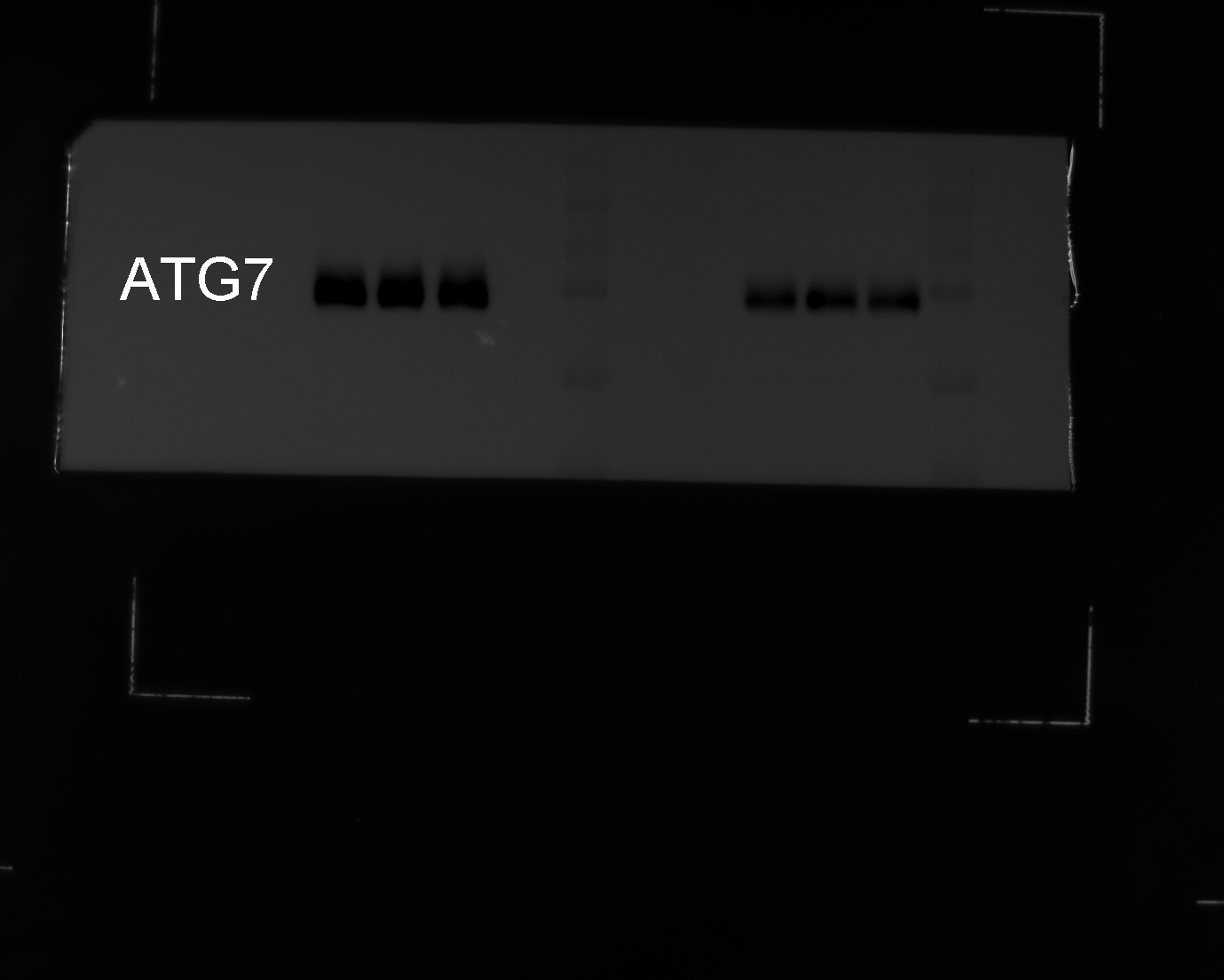

Supplement: Figure 7—figure supplement 1—source data 1. [file elife-69047-fig7-figsupp1-data1.zip › Figure 7-figure supplement 1-source data 1. Original western blot files for Figure 7-figure supplement 1/Figure 7-figure supplement 1-source data E1.jpg]

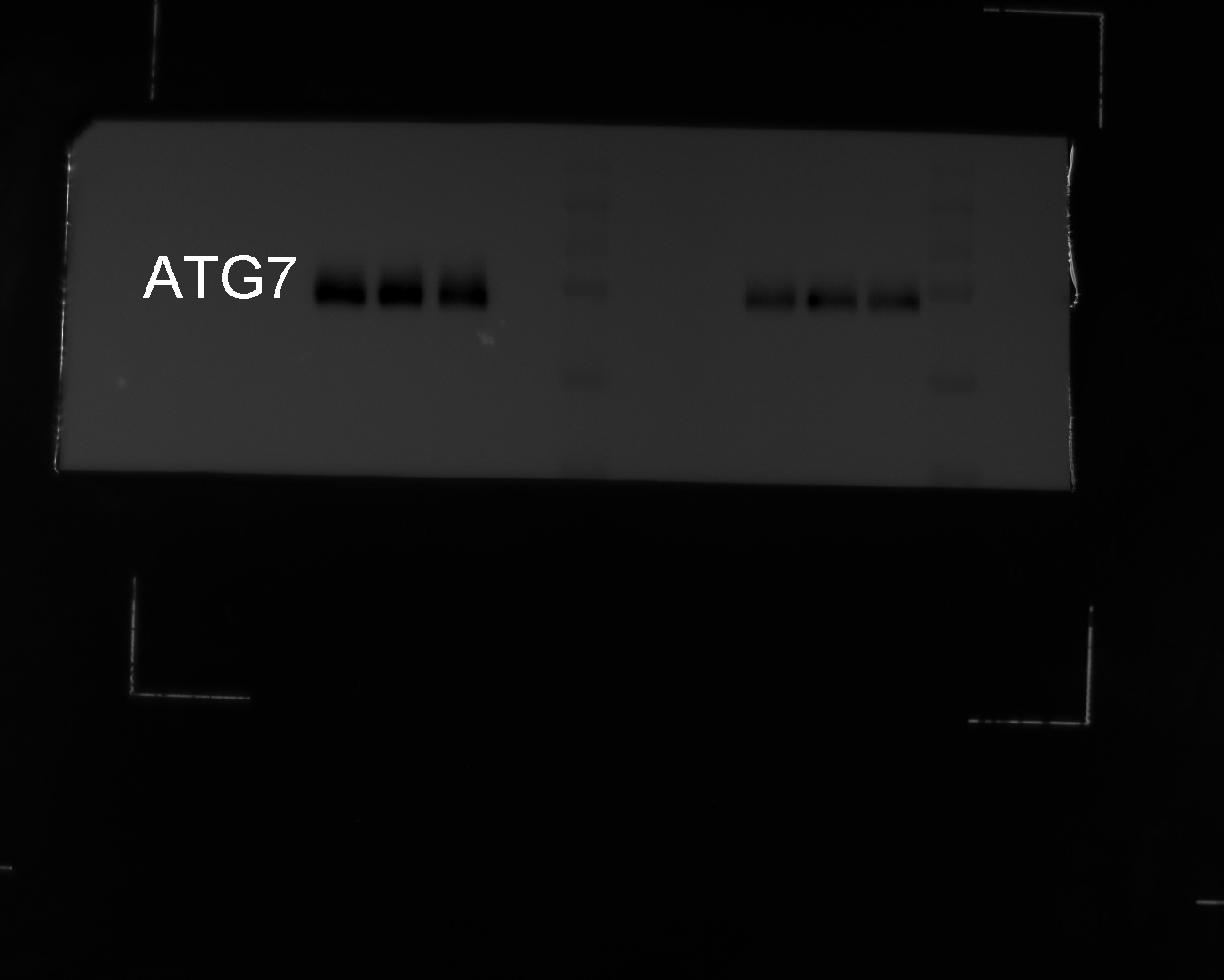

Supplement: Figure 7—figure supplement 1—source data 1. [file elife-69047-fig7-figsupp1-data1.zip › Figure 7-figure supplement 1-source data 1. Original western blot files for Figure 7-figure supplement 1/Figure 7-figure supplement 1-source data E2.jpg]

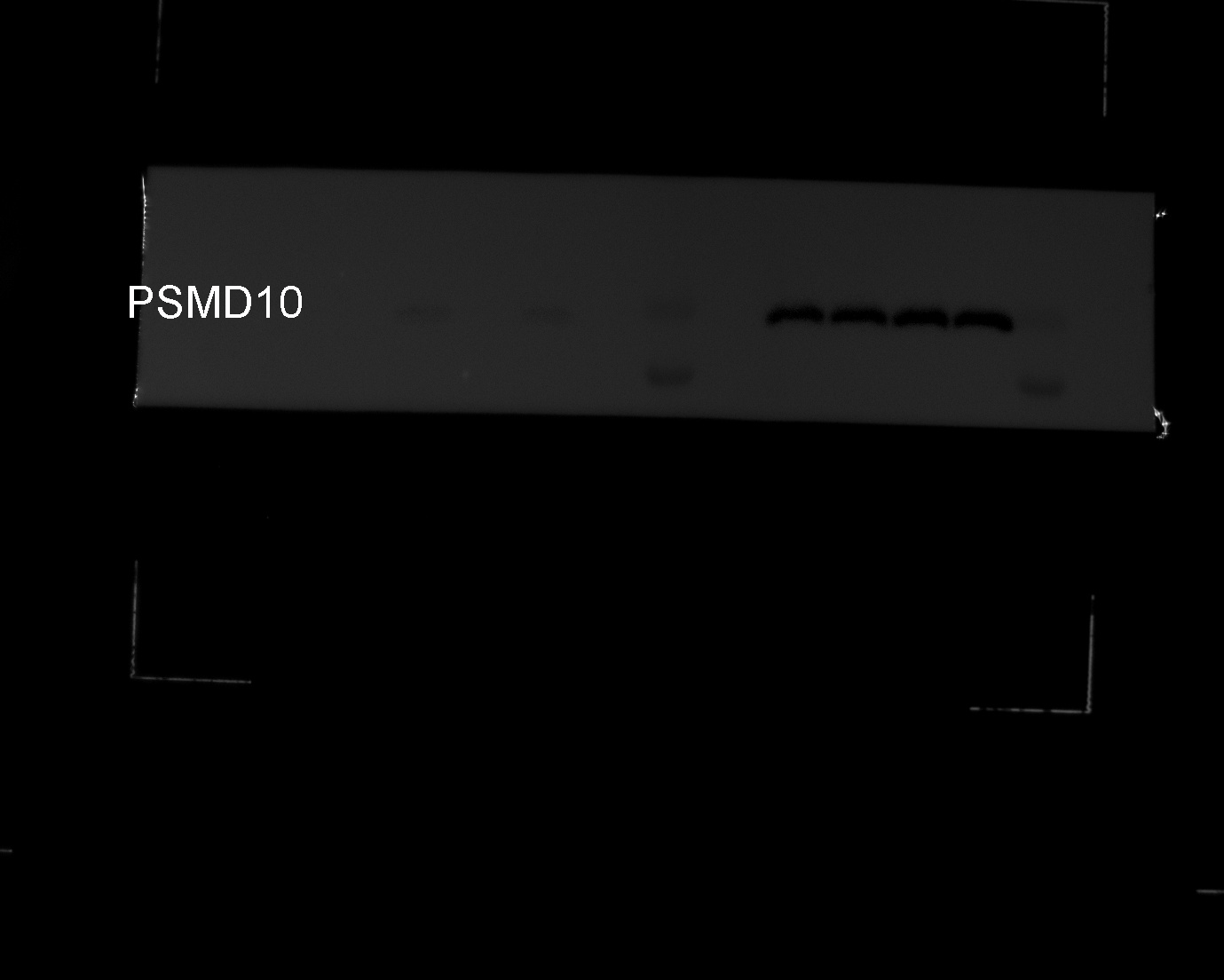

Supplement: Figure 7—figure supplement 1—source data 1. [file elife-69047-fig7-figsupp1-data1.zip › Figure 7-figure supplement 1-source data 1. Original western blot files for Figure 7-figure supplement 1/Figure 7-figure supplement 1-source data E3.jpg]

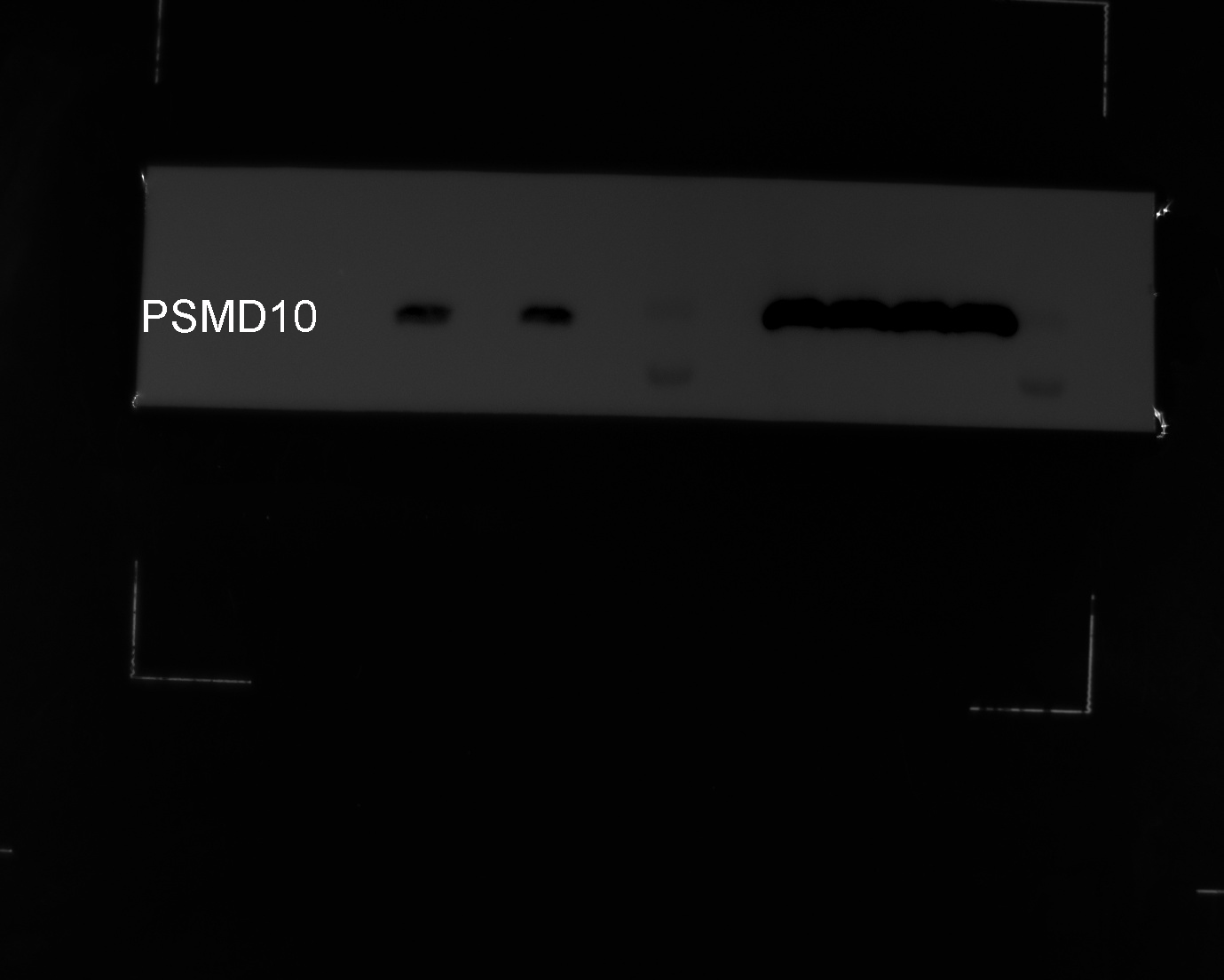

Supplement: Figure 7—figure supplement 1—source data 1. [file elife-69047-fig7-figsupp1-data1.zip › Figure 7-figure supplement 1-source data 1. Original western blot files for Figure 7-figure supplement 1/Figure 7-figure supplement 1-source data E4.jpg]

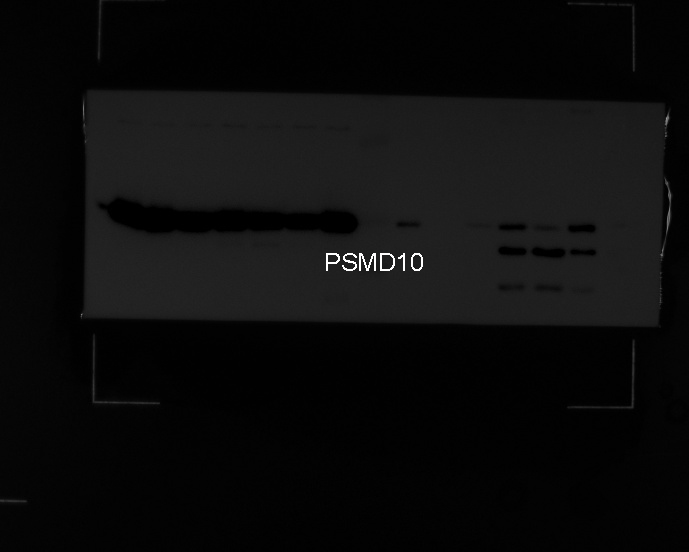

Supplement: Figure 7—figure supplement 1—source data 1. [file elife-69047-fig7-figsupp1-data1.zip › Figure 7-figure supplement 1-source data 1. Original western blot files for Figure 7-figure supplement 1/Figure 7-figure supplement 1-source data F1.jpg]

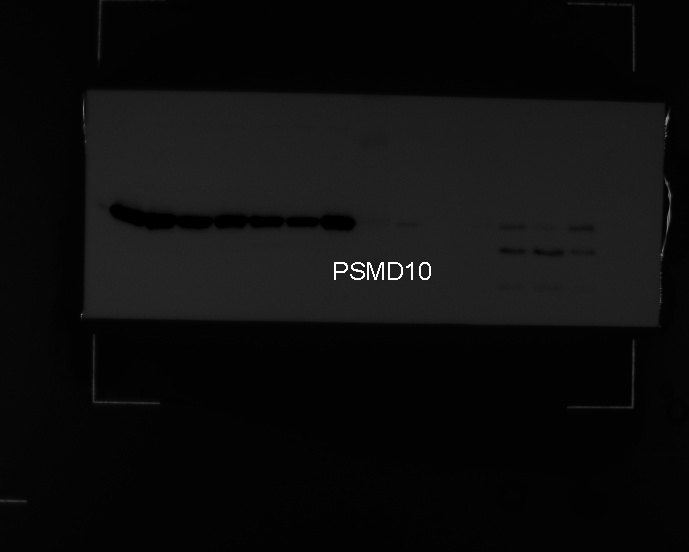

Supplement: Figure 7—figure supplement 1—source data 1. [file elife-69047-fig7-figsupp1-data1.zip › Figure 7-figure supplement 1-source data 1. Original western blot files for Figure 7-figure supplement 1/Figure 7-figure supplement 1-source data F2.jpg]

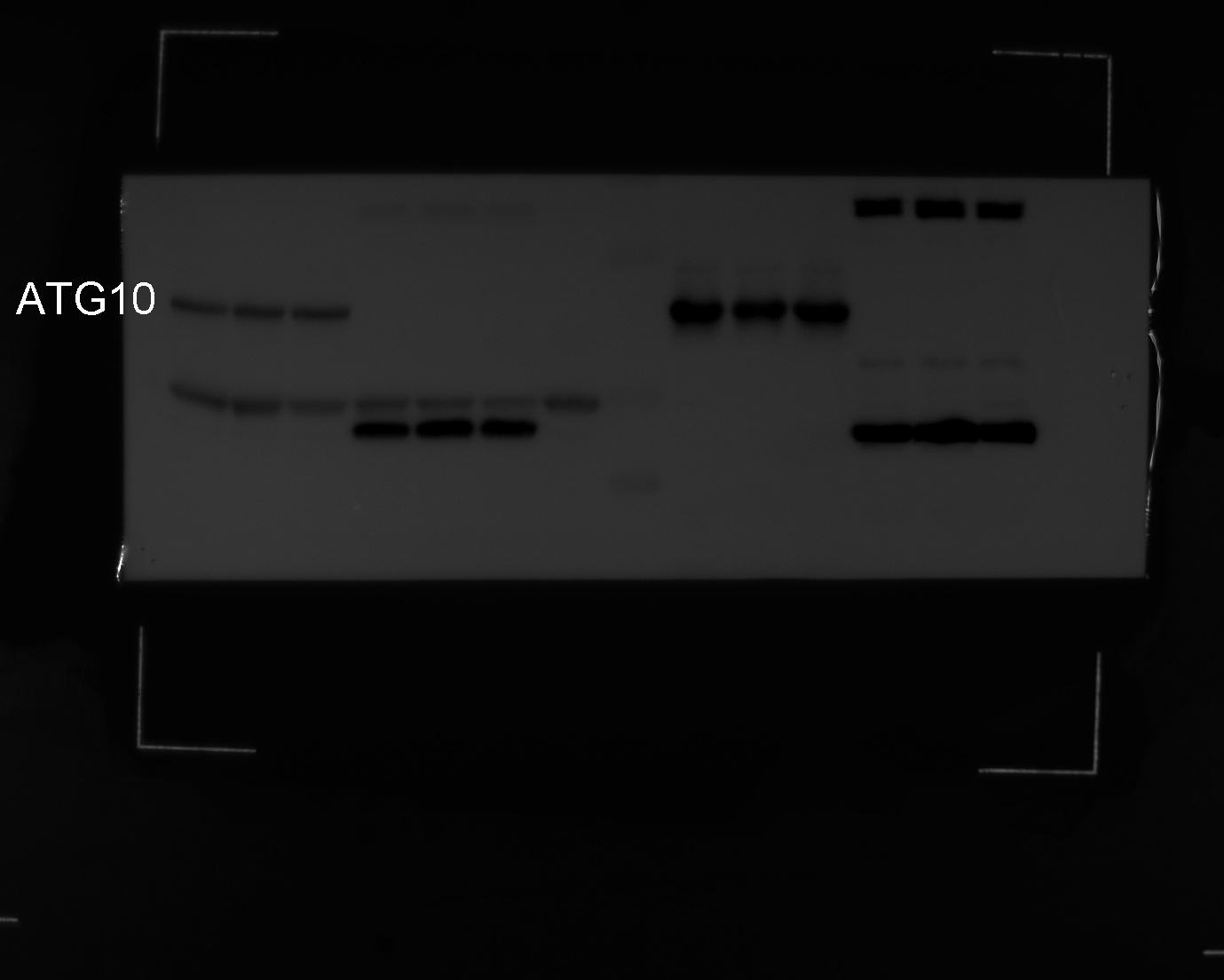

Supplement: Figure 7—figure supplement 1—source data 1. [file elife-69047-fig7-figsupp1-data1.zip › Figure 7-figure supplement 1-source data 1. Original western blot files for Figure 7-figure supplement 1/Figure 7-figure supplement 1-source data F3.jpg]

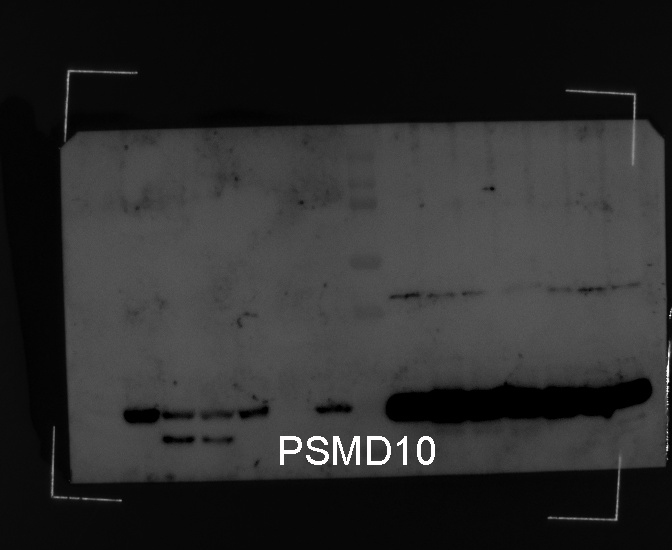

Supplement: Figure 7—figure supplement 3—source data 1. [file elife-69047-fig7-figsupp3-data1.zip › Figure 7-figure supplement 3-source data 1. Original western blot files for Figure 7-figure supplement 3/Figure 7-figure supplement 3-source data B1.jpg]

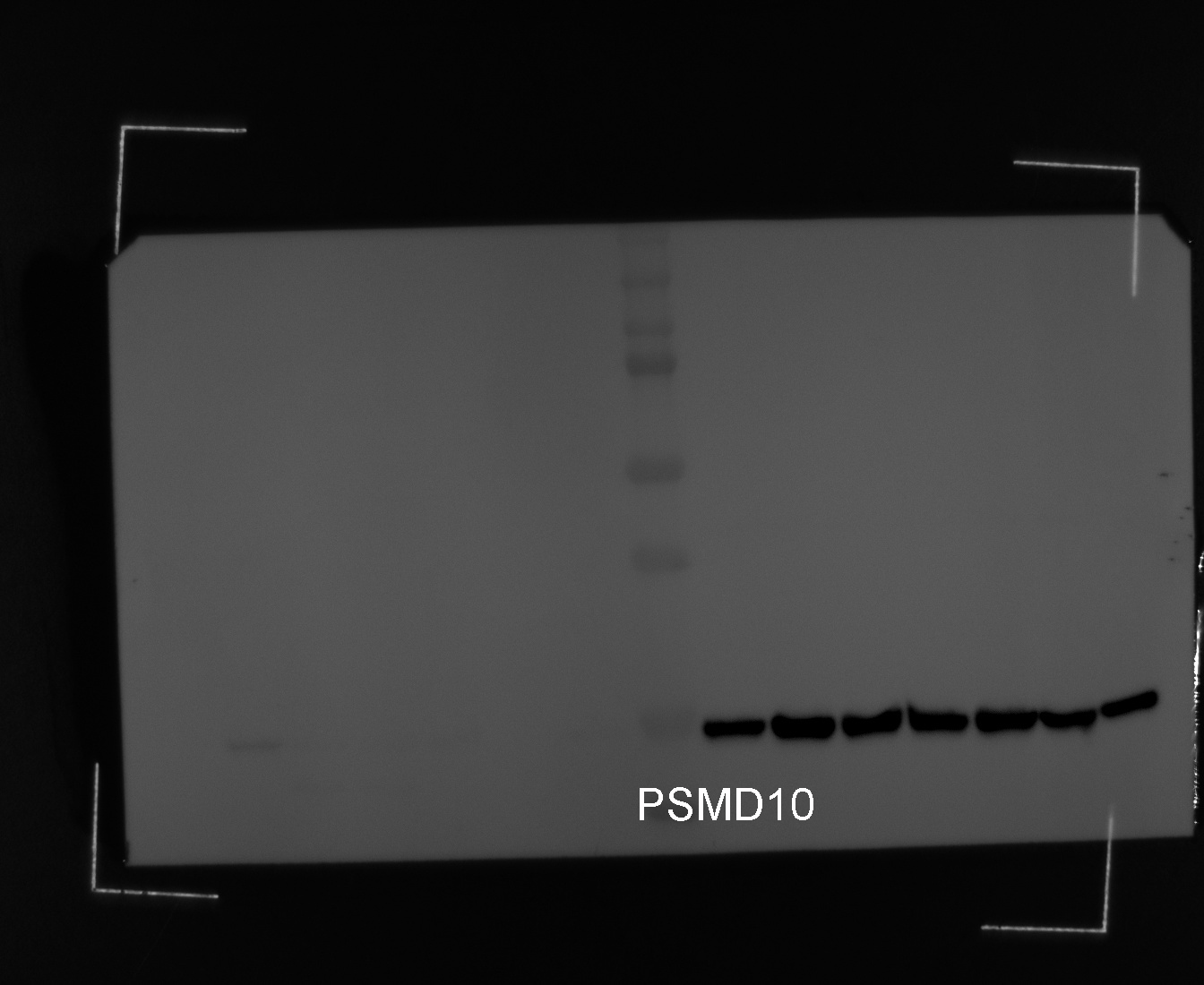

Supplement: Figure 7—figure supplement 3—source data 1. [file elife-69047-fig7-figsupp3-data1.zip › Figure 7-figure supplement 3-source data 1. Original western blot files for Figure 7-figure supplement 3/Figure 7-figure supplement 3-source data B2.jpg]

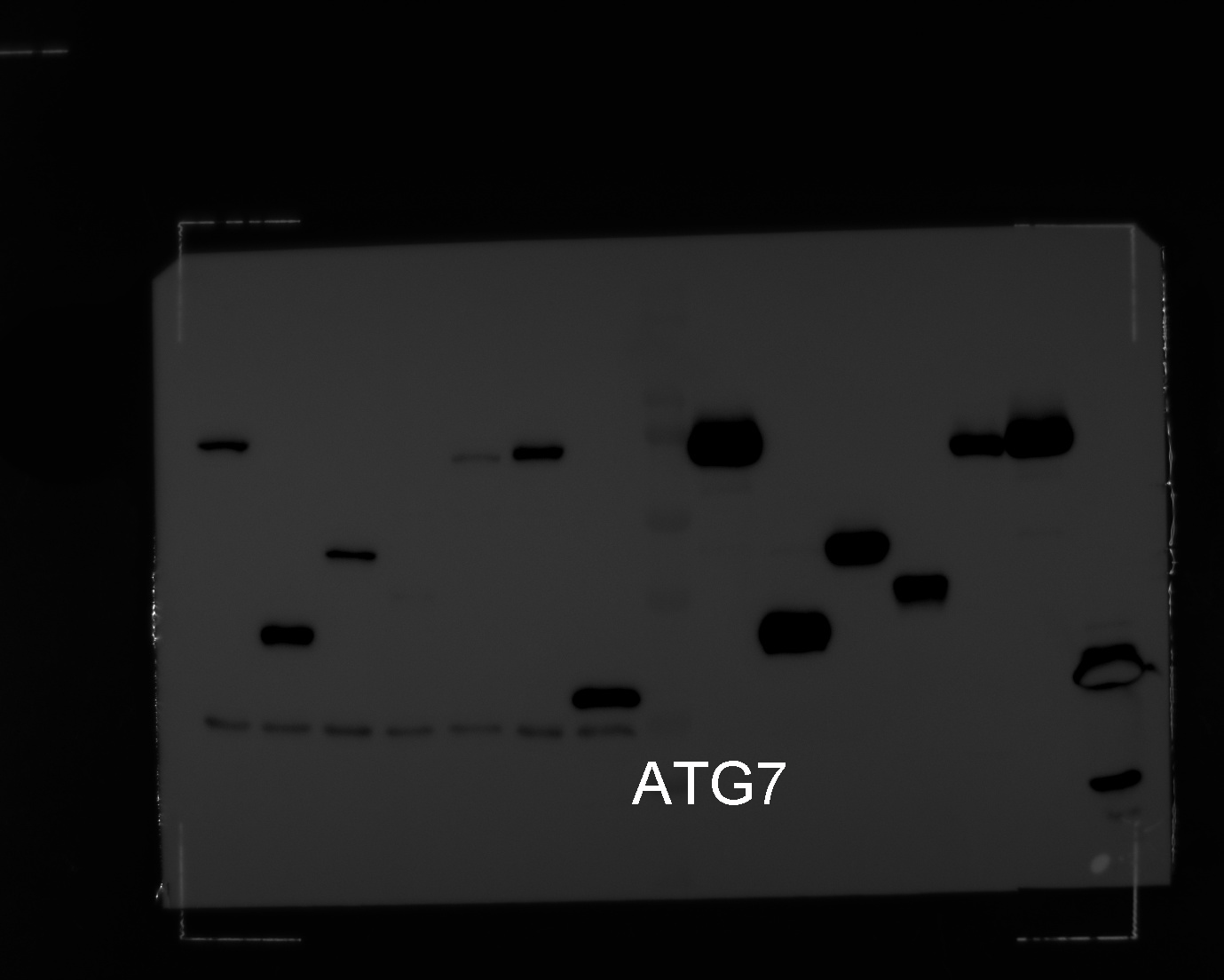

Supplement: Figure 7—figure supplement 3—source data 1. [file elife-69047-fig7-figsupp3-data1.zip › Figure 7-figure supplement 3-source data 1. Original western blot files for Figure 7-figure supplement 3/Figure 7-figure supplement 3-source data B3.jpg]

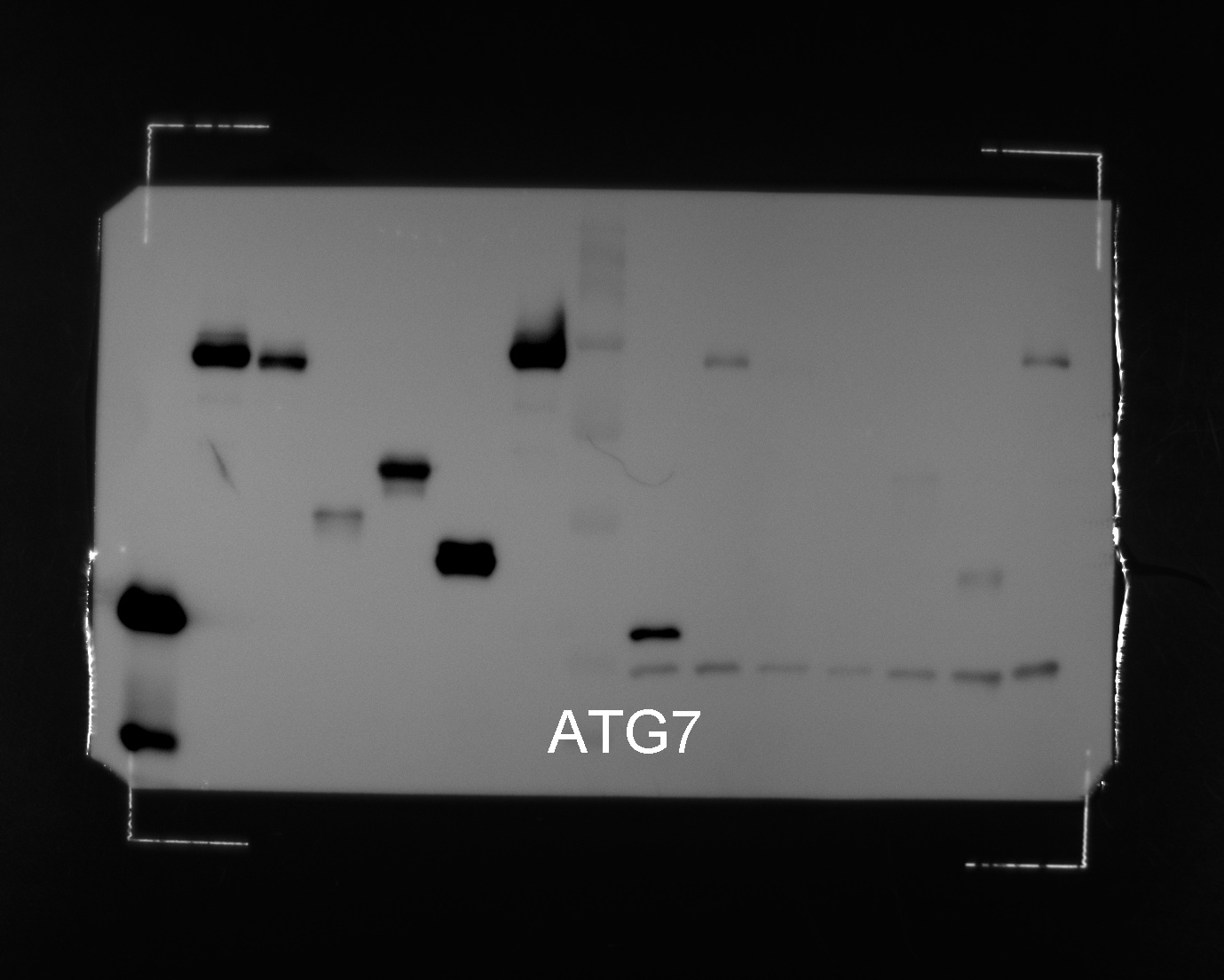

Supplement: Figure 7—figure supplement 3—source data 1. [file elife-69047-fig7-figsupp3-data1.zip › Figure 7-figure supplement 3-source data 1. Original western blot files for Figure 7-figure supplement 3/Figure 7-figure supplement 3-source data B4.jpg]

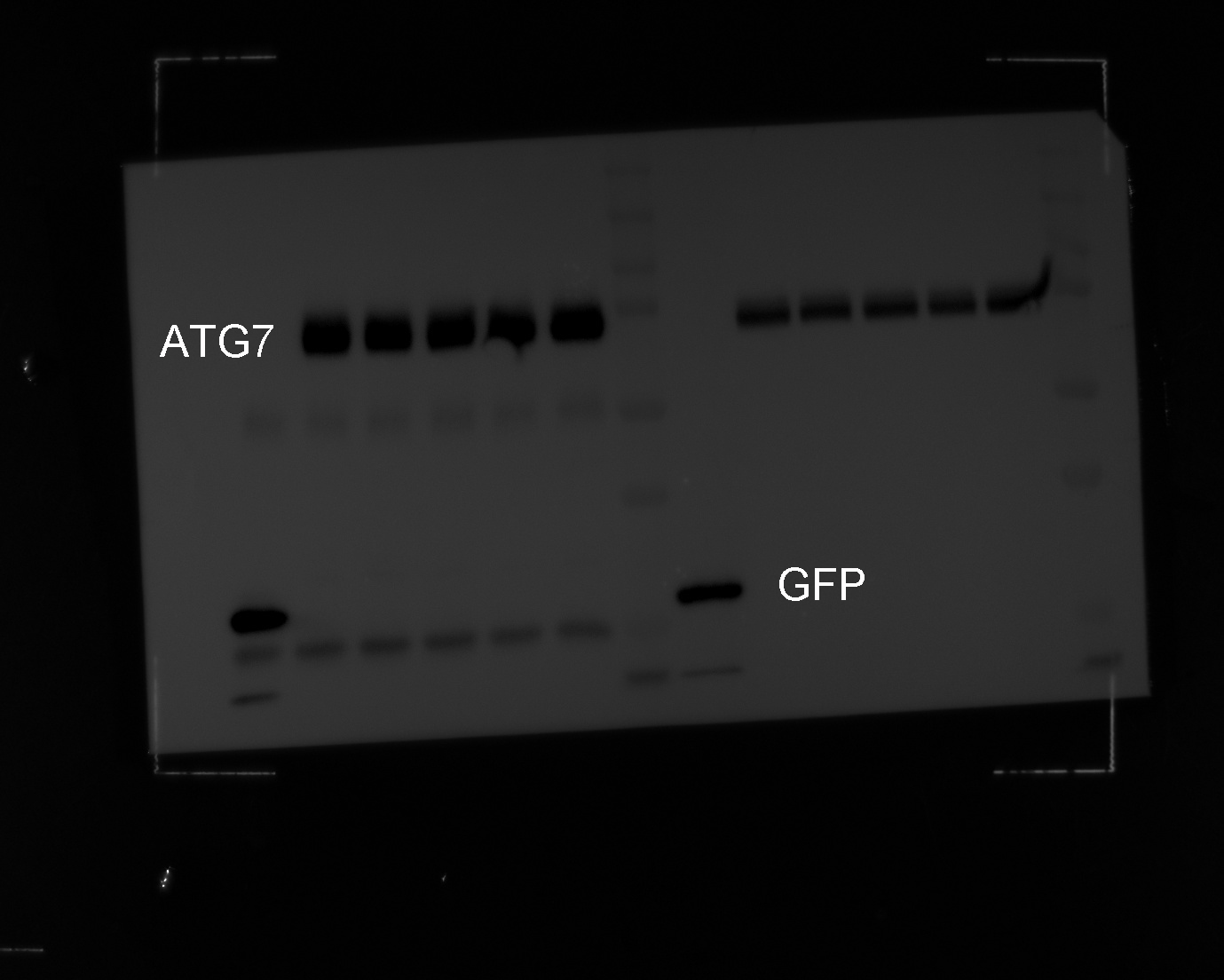

Supplement: Figure 7—figure supplement 3—source data 1. [file elife-69047-fig7-figsupp3-data1.zip › Figure 7-figure supplement 3-source data 1. Original western blot files for Figure 7-figure supplement 3/Figure 7-figure supplement 3-source data C1.jpg]

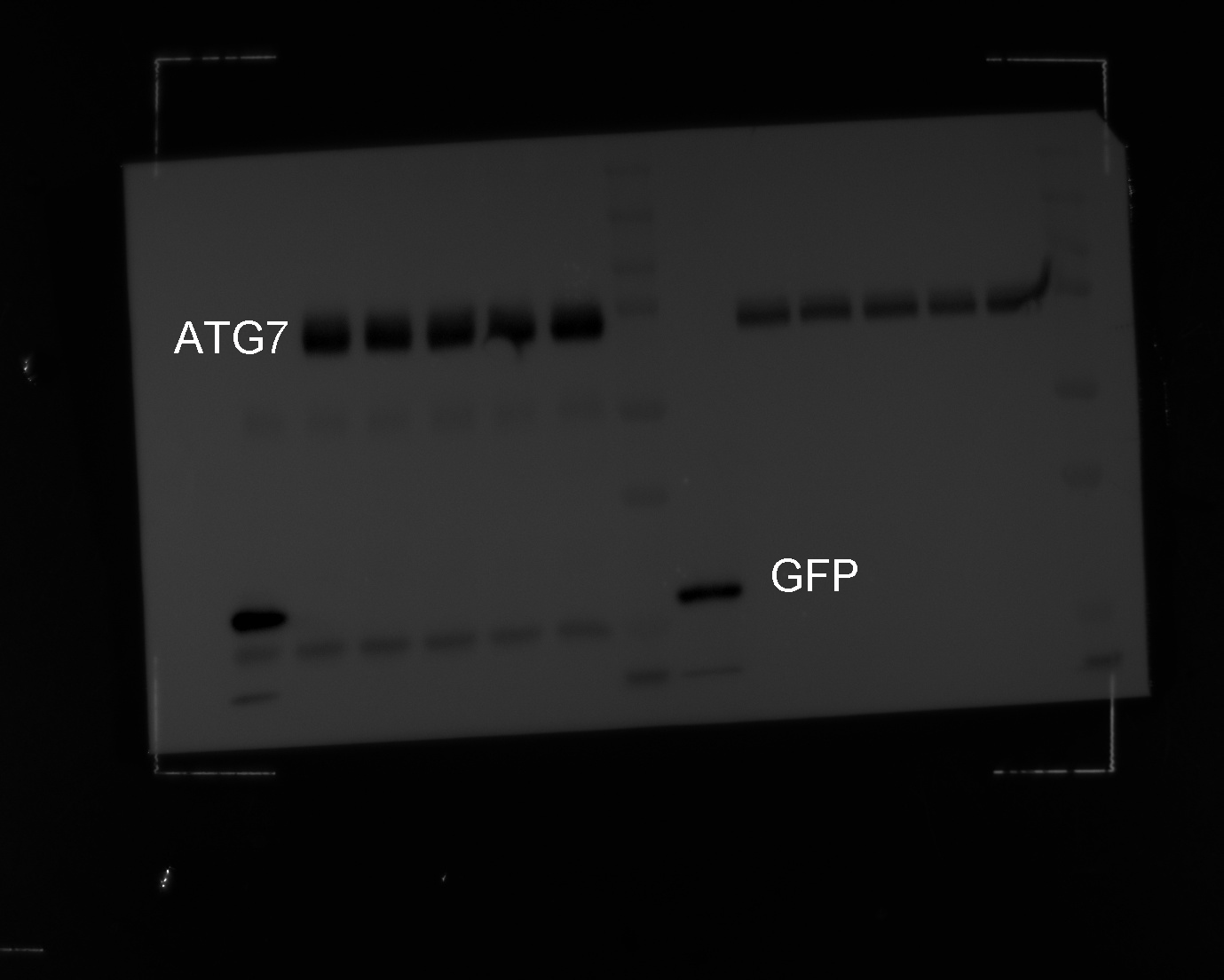

Supplement: Figure 7—figure supplement 3—source data 1. [file elife-69047-fig7-figsupp3-data1.zip › Figure 7-figure supplement 3-source data 1. Original western blot files for Figure 7-figure supplement 3/Figure 7-figure supplement 3-source data C2.jpg]

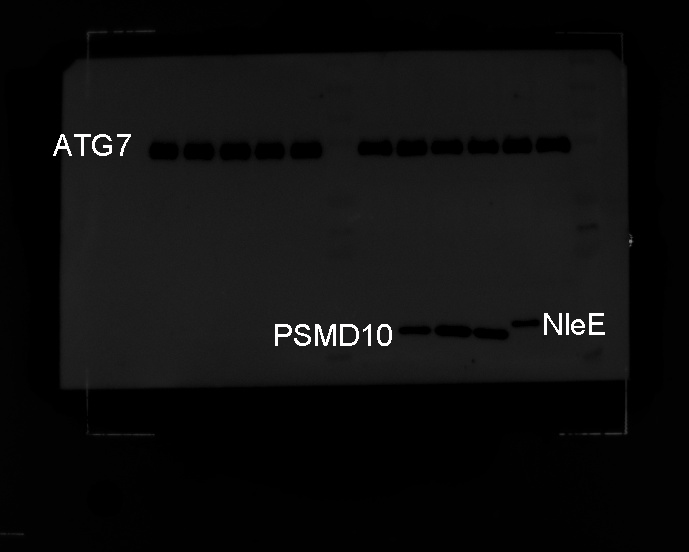

Supplement: Figure 7—figure supplement 3—source data 1. [file elife-69047-fig7-figsupp3-data1.zip › Figure 7-figure supplement 3-source data 1. Original western blot files for Figure 7-figure supplement 3/Figure 7-figure supplement 3-source data C3.jpg]

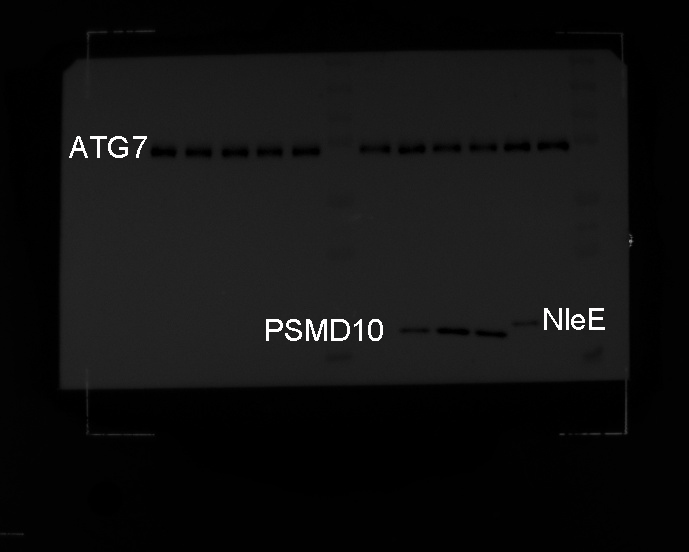

Supplement: Figure 7—figure supplement 3—source data 1. [file elife-69047-fig7-figsupp3-data1.zip › Figure 7-figure supplement 3-source data 1. Original western blot files for Figure 7-figure supplement 3/Figure 7-figure supplement 3-source data C4.jpg]

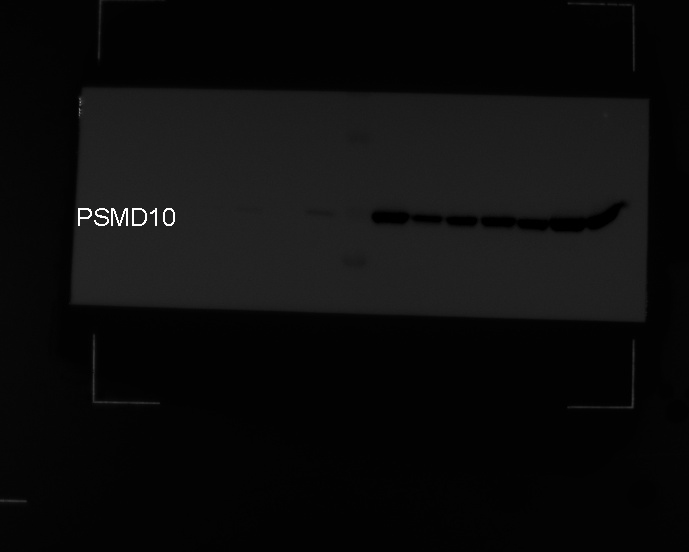

Supplement: Figure 7—figure supplement 3—source data 1. [file elife-69047-fig7-figsupp3-data1.zip › Figure 7-figure supplement 3-source data 1. Original western blot files for Figure 7-figure supplement 3/Figure 7-figure supplement 3-source data DE1.jpg]

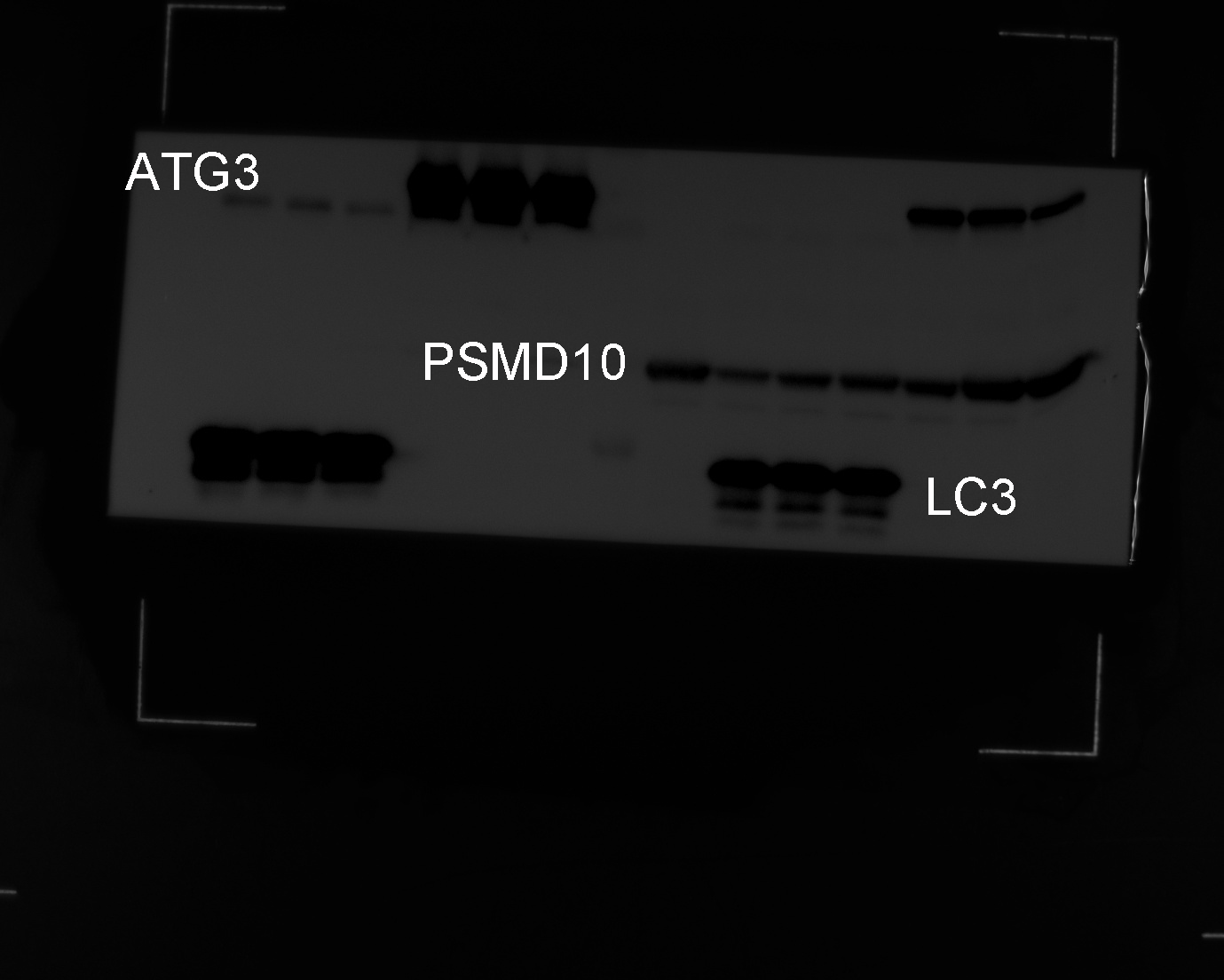

Supplement: Figure 7—figure supplement 3—source data 1. [file elife-69047-fig7-figsupp3-data1.zip › Figure 7-figure supplement 3-source data 1. Original western blot files for Figure 7-figure supplement 3/Figure 7-figure supplement 3-source data DE2.jpg]

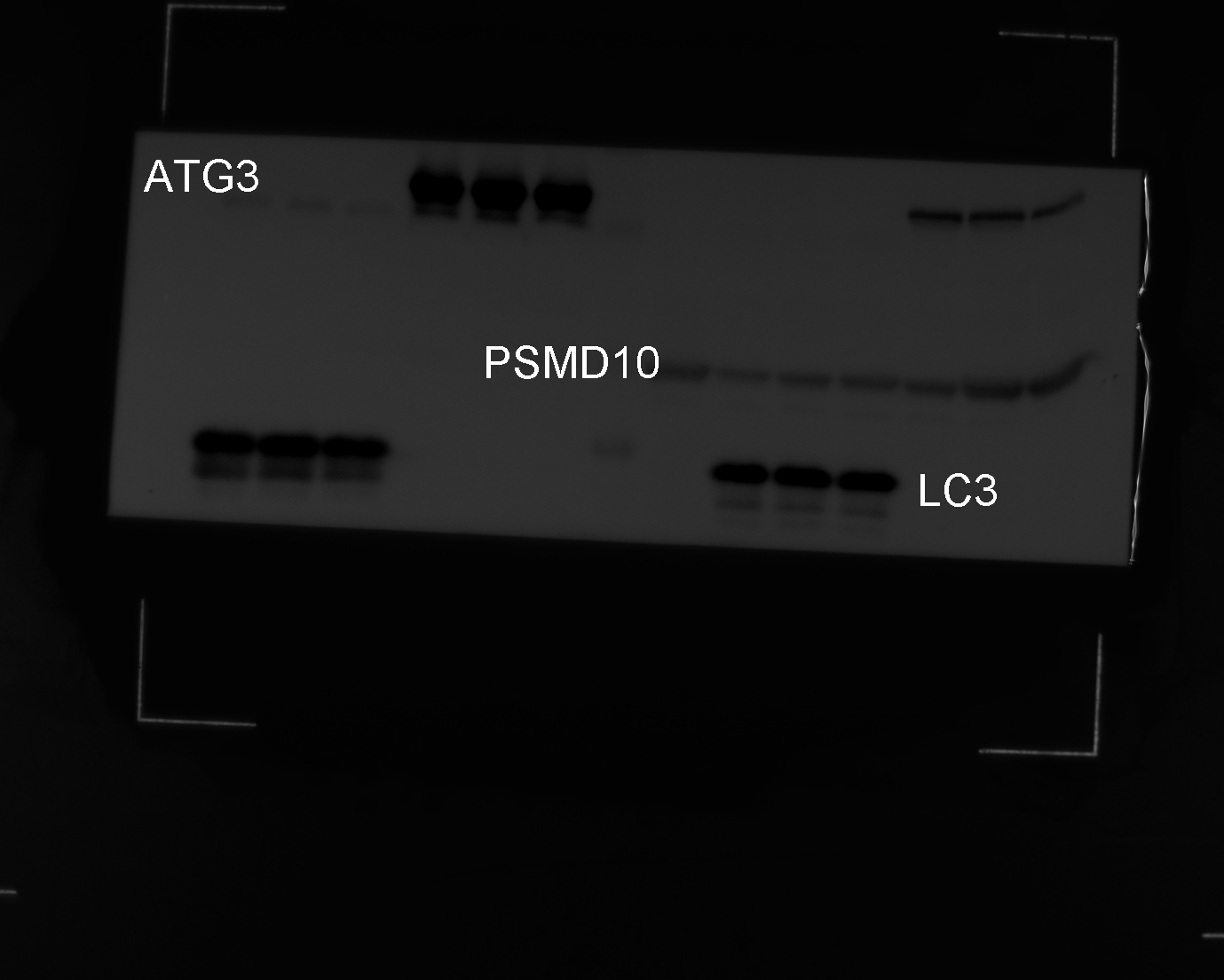

Supplement: Figure 7—figure supplement 3—source data 1. [file elife-69047-fig7-figsupp3-data1.zip › Figure 7-figure supplement 3-source data 1. Original western blot files for Figure 7-figure supplement 3/Figure 7-figure supplement 3-source data DE3.jpg]

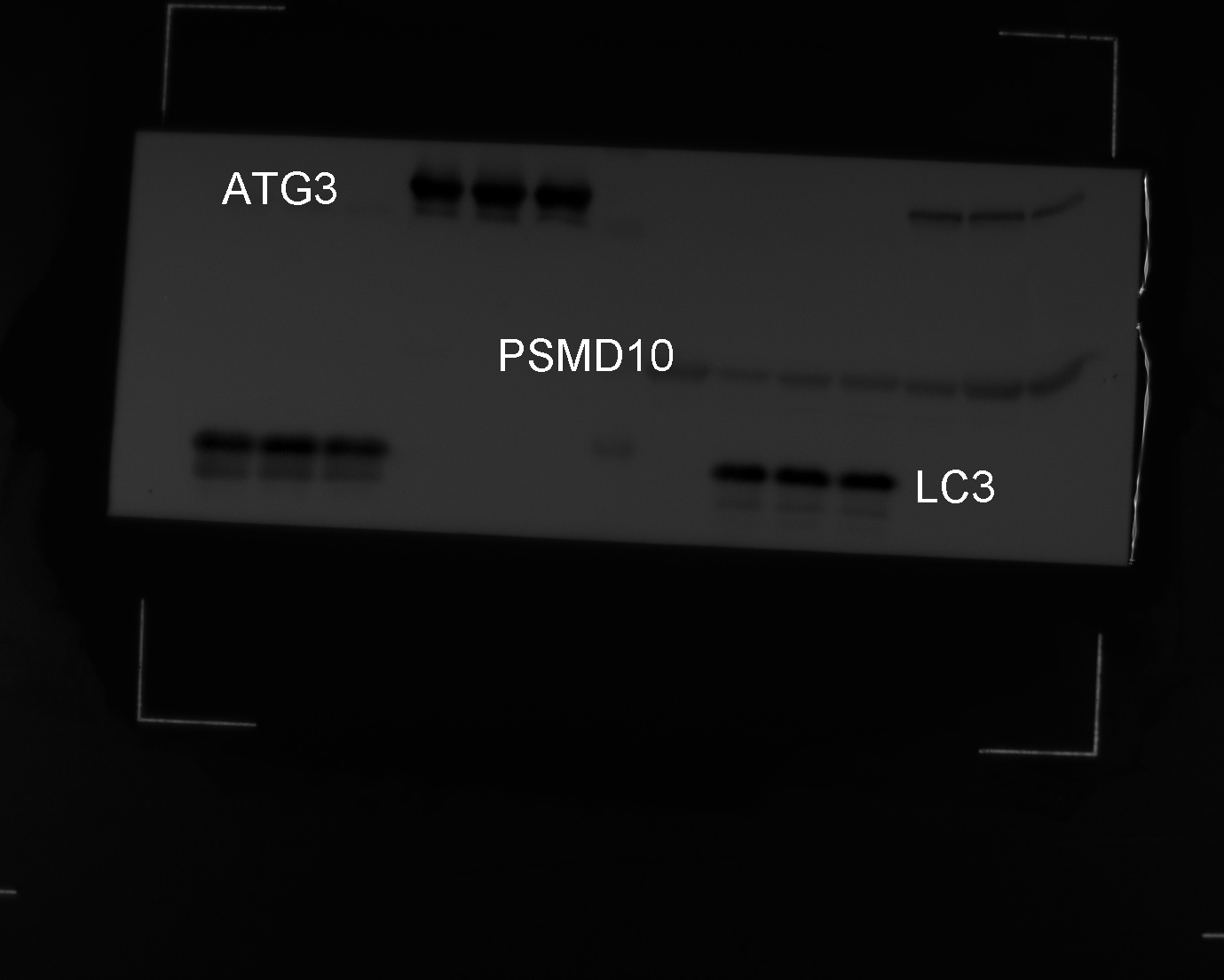

Supplement: Figure 7—figure supplement 3—source data 1. [file elife-69047-fig7-figsupp3-data1.zip › Figure 7-figure supplement 3-source data 1. Original western blot files for Figure 7-figure supplement 3/Figure 7-figure supplement 3-source data DE4.jpg]

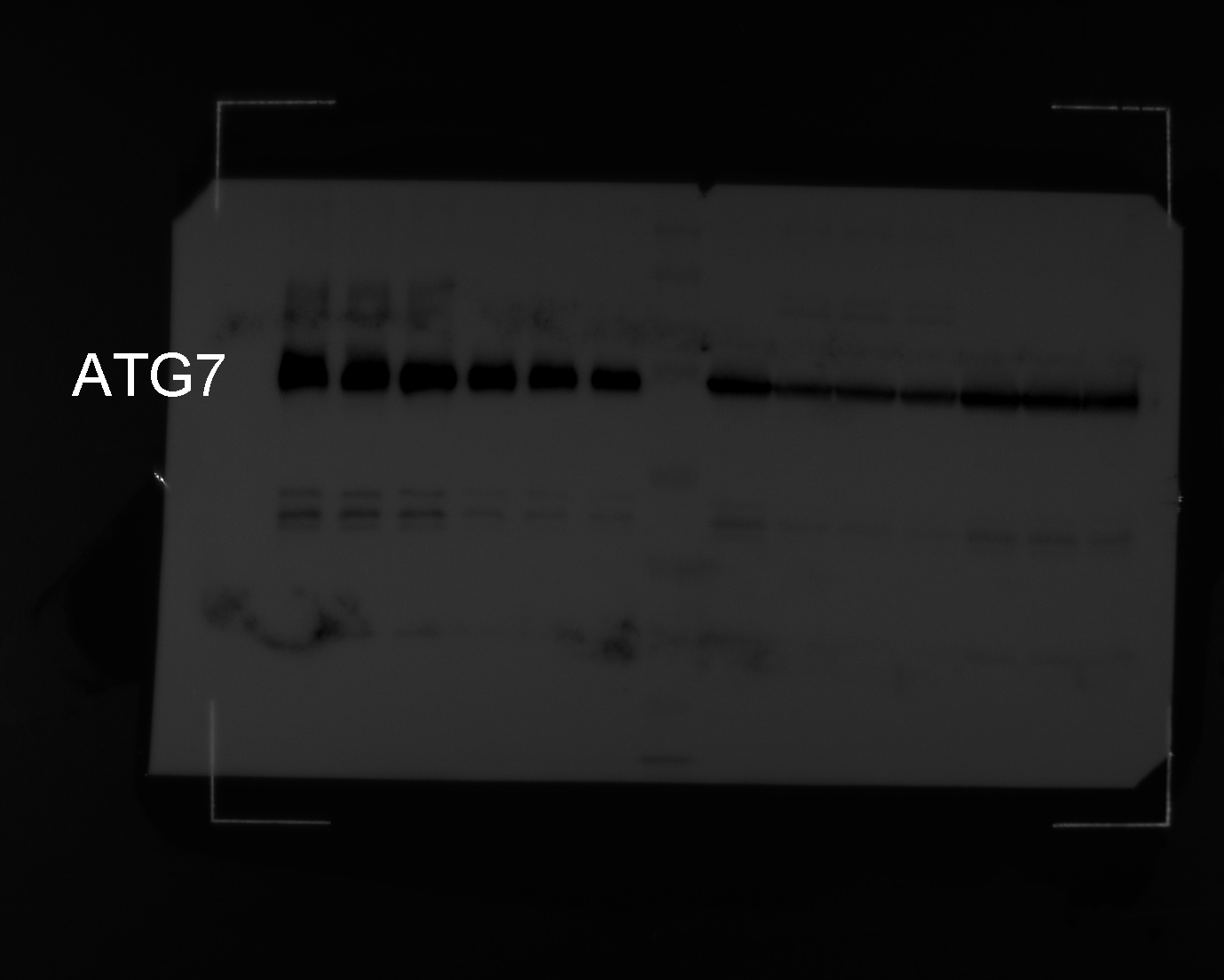

Supplement: Figure 7—figure supplement 3—source data 1. [file elife-69047-fig7-figsupp3-data1.zip › Figure 7-figure supplement 3-source data 1. Original western blot files for Figure 7-figure supplement 3/Figure 7-figure supplement 3-source data DE5.jpg]

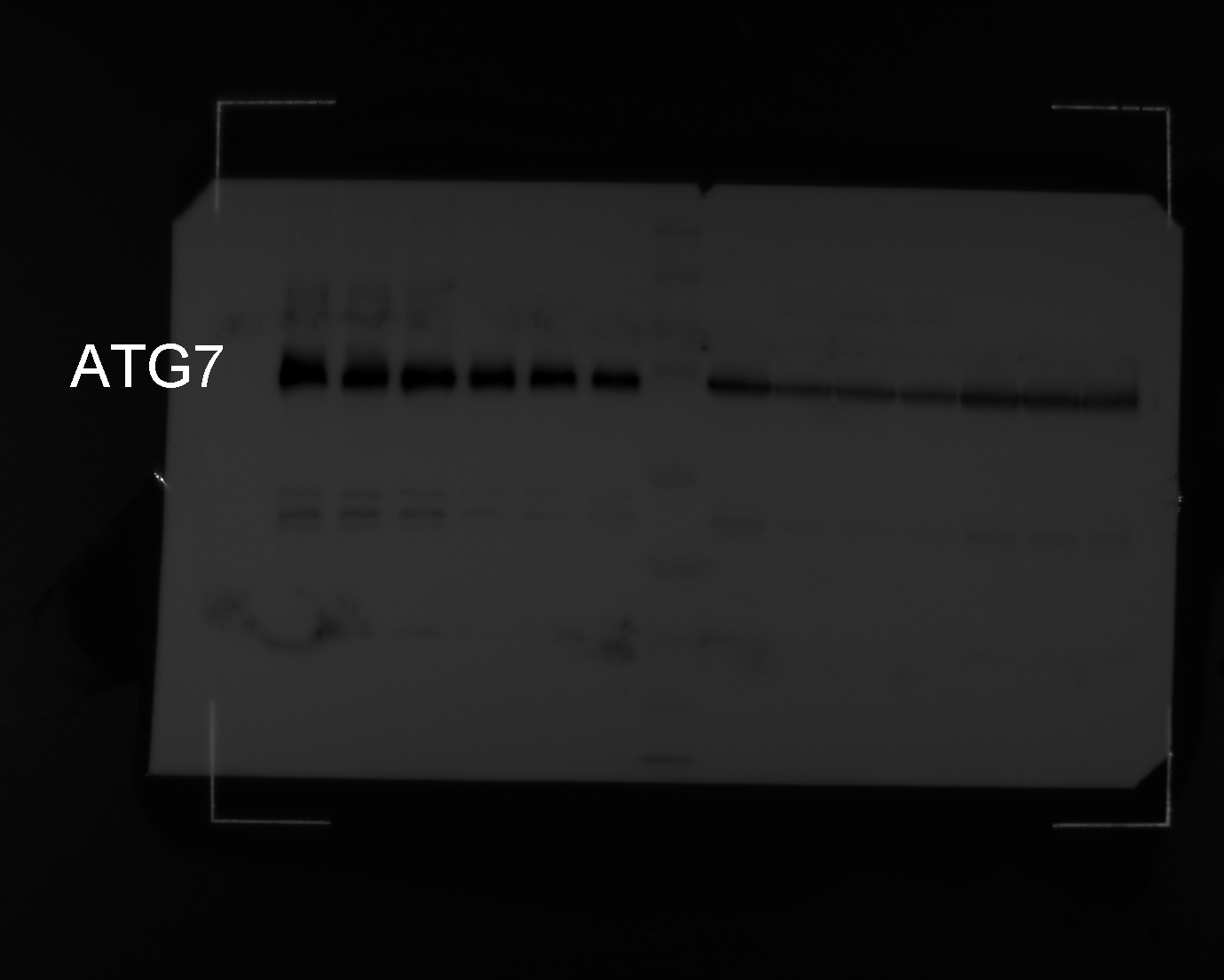

Supplement: Figure 7—figure supplement 3—source data 1. [file elife-69047-fig7-figsupp3-data1.zip › Figure 7-figure supplement 3-source data 1. Original western blot files for Figure 7-figure supplement 3/Figure 7-figure supplement 3-source data DE6.jpg]
