# Supplementary material for: Genetically incorporated crosslinkers reveal NleE attenuates host autophagy dependent on PSMD10
Source: eLife. 2021 Jul 13;10:e69047. doi: 10.7554/eLife.69047 (PMC8324295; doi:10.7554/eLife.69047)
Supplement: Supplementary file 1. [file elife-69047-supp1.doc]

| **Key Resources Table** | | | | |
| --- | --- | --- | --- | --- |
| **Reagent type (species) or resource** | **Designation** | **Source or reference** | **Identifiers** | **Additional information** |
| sequence-based reagent | NleE-Y42TAG-F | This paper | PCR primers | GCCCGAATATTTAGGATAAAAAGTATATTAGCGGCATTACCC |
| sequence-based reagent | NleE-Y42TAG-R | This paper | PCR primers | ATCCTAAATATTCGGGCTTTCATTAAAATAATC |
| sequence-based reagent | NleE-S113TAG-F | This paper | PCR primers | GCCAGTTTTAGGTGGATAGCCTGTATAACCCGG |
| sequence-based reagent | NleE-S113TAG-R | This paper | PCR primers | ATCCACCTAAAACTGGCCCATCGGGCGTTTAC |
| sequence-based reagent | NleE-V114TAG-F | This paper | PCR primers | GTTTAGCTAGGATAGCCTGTATAACCCGGACCT |
| sequence-based reagent | NleE-V114TAG-R | This paper | PCR primers | GGCTATCCTAGCTAAACTGGCCCATCGGGCGT |
| sequence-based reagent | NleE-D115TAG-F | This paper | PCR primers | TAGCGTGTAGAGCCTGTATAACCCGGACCTGC |
| sequence-based reagent | NleE-D115TAG-R | This paper | PCR primers | ACAGGCTCTACACGCTAAACTGGCCCATCGGG |
| sequence-based reagent | NleE-K134TAG-F | This paper | PCR primers | GCTGCTAGATTTTTCCGAAAGAAAACAATGATT |
| sequence-based reagent | NleE-K134TAG-R | This paper | PCR primers | CGGAAAAATCTAGCAGCAGATATCCGGCAGTT |
| sequence-based reagent | NleE-F136TAG-F | This paper | PCR primers | GCTGCAAAATTTAGCCGAAAGAAAACAATGATTTCCTG |
| sequence-based reagent | NleE-F136TAG-R | This paper | PCR primers | CGGCTAAATTTTGCAGCAGATATCCGGCAGTT |
| sequence-based reagent | NleE-K138TAG-F | This paper | PCR primers | TCCGTAGGAAAACAATGATTTCCTGTATATCGTT |
| sequence-based reagent | NleE-K138TAG-R | This paper | PCR primers | CATTGTTTTCCTACGGAAAAATTTTGCAGCAGATA |
| sequence-based reagent | NleE-E139TAG-F | This paper | PCR primers | TCCGAAATAGAACAATGATTTCCTGTATATCGTTGTG |
| sequence-based reagent | NleE-E139TAG-R | This paper | PCR primers | CATTGTTCTATTTCGGAAAAATTTTGCAGCAG |
| sequence-based reagent | NleE-N140TAG-F | This paper | PCR primers | CCGAAAGAATAGAATGATTTCCTGTATATCGTTGTGGT |
| sequence-based reagent | NleE-N140TAG-R | This paper | PCR primers | TCATTCTATTCTTTCGGAAAAATTTTGCAGCA |
| sequence-based reagent | NleE-N141TAG-F | This paper | PCR primers | CCGAAAGAAAACTAGGATTTCCTGTATATCGTTGTGGTGTAC |
| sequence-based reagent | NleE-N141TAG-R | This paper | PCR primers | TCCTAGTTTTCTTTCGGAAAAATTTTGCAGCA |
| sequence-based reagent | NleE-F143TAG-F | This paper | PCR primers | TGATTAGCTGTATATCGTTGTGGTGTACCGCA |
| sequence-based reagent | NleE-F143TAG-R | This paper | PCR primers | CGATATACAGCTAATCATTGTTTTCTTTCGGAAAAATT |
| sequence-based reagent | NleE-L144TAG-F | This paper | PCR primers | CTAGTATATCGTTGTGGTGTACCGCAACGACA |
| sequence-based reagent | NleE-L144TAG-R | This paper | PCR primers | CCACAACGATATACTAGAAATCATTGTTTTCTTTCGGAAA |
| sequence-based reagent | NleE-V148TAG-F | This paper | PCR primers | ATCGTTTAGGTGTACCGCAACGACAGCCCGCT |
| sequence-based reagent | NleE-V148TAG-R | This paper | PCR primers | CGGTACACCTAAACGATATACAGGAAATCATTGTTTTC |
| sequence-based reagent | NleE-F164TAG-F | This paper | PCR primers | CAATCGCTAGATCGAACTGTACAACATCAAGCGC |
| sequence-based reagent | NleE-F164TAG-R | This paper | PCR primers | GTTCGATCTAGCGATTGGCACGCTGTTCGCCC |
| sequence-based reagent | NleE-I174TAG-F | This paper | PCR primers | AAGCGCGACTAGATGCAGGAGCTGAACTACGAACT |
| sequence-based reagent | NleE-I174TAG-R | This paper | PCR primers | TGCATCTAGTCGCGCTTGATGTTGTACAGTTC |
| sequence-based reagent | NleE-M175TAG-F | This paper | PCR primers | GACATCTAGCAGGAGCTGAACTACGAACTGCC |
| sequence-based reagent | NleE-M175TAG-R | This paper | PCR primers | AGCTCCTGCTAGATGTCGCGCTTGATGTTGTAC |
| sequence-based reagent | NleE-A187TAG-F | This paper | PCR primers | CGGAACTGAAATAGGTGAAGAGTGAAATGATCATCGCC |
| sequence-based reagent | NleE-A187TAG-R | This paper | PCR primers | CACCTATTTCAGTTCCGGCAGTTCGTAGTTCA |
| sequence-based reagent | NleE-V188TAG-F | This paper | PCR primers | GAAAGCCTAGAAGAGTGAAATGATCATCGCCC |
| sequence-based reagent | NleE-V188TAG-R | This paper | PCR primers | CACTCTTCTAGGCTTTCAGTTCCGGCAGTTCG |
| sequence-based reagent | NleE-K189TAG-F | This paper | PCR primers | AGCCGTGTAGAGTGAAATGATCATCGCCCGCG |
| sequence-based reagent | NleE-K189TAG-R | This paper | PCR primers | TTTCACTCTACACGGCTTTCAGTTCCGGCAGT |
| sequence-based reagent | NleE-S190TAG-F | This paper | PCR primers | CCGTGAAGTAGGAAATGATCATCGCCCGCGAA |
| sequence-based reagent | NleE-S190TAG-R | This paper | PCR primers | CATTTCCTACTTCACGGCTTTCAGTTCCGGCA |
| sequence-based reagent | NleE-E191TAG-F | This paper | PCR primers | CCGTGAAGAGTTAGATGATCATCGCCCGCGAA |
| sequence-based reagent | NleE-E191TAG-R | This paper | PCR primers | CATCTAACTCTTCACGGCTTTCAGTTCCGGCA |
| sequence-based reagent | NleE-M192TAG-F | This paper | PCR primers | GTGAATAGATCATCGCCCGCGAAATGGGCGAG |
| sequence-based reagent | NleE-M192TAG-R | This paper | PCR primers | GGCGATGATCTATTCACTCTTCACGGCTTTCAGTT |
| sequence-based reagent | NleE-Y212TAG-F | This paper | PCR primers | CGAAATCGACAGCTAGATGAAATATATTAACAACAAACTGAGCAA |
| sequence-based reagent | NleE-Y212TAG-R | This paper | PCR primers | TCTAGCTGTCGATTTCGCCCGGCATATAGCTG |
| sequence-based reagent | NleE-M213TAG-F | This paper | PCR primers | CGACAGCTATTAGAAATATATTAACAACAAACTGAGCAAAATT |
| sequence-based reagent | NleE-M213TAG-R | This paper | PCR primers | ATTTCTAATAGCTGTCGATTTCGCCCGGCATA |
| sequence-based reagent | NleE-K214TAG-F | This paper | PCR primers | CGACAGCTATATGTAGTATATTAACAACAAACTGAGCAAAATTGA |
| sequence-based reagent | NleE-K214TAG-R | This paper | PCR primers | ACTACATATAGCTGTCGATTTCGCCCGGCATA |
| sequence-based reagent | NleE-Y215TAG-F | This paper | PCR primers | CAGCTATATGAAATAGATTAACAACAAACTGAGCAAAATTGA |
| sequence-based reagent | NleE-Y215TAG-R | This paper | PCR primers | TCTATTTCATATAGCTGTCGATTTCGCCCGGC |
| sequence-based reagent | NleE-I216TAG-F | This paper | PCR primers | TAGAACAACAAACTGAGCAAAATTGAAGATTA |
| sequence-based reagent | NleE-I216TAG-R | This paper | PCR primers | CTCAGTTTGTTGTTCTAATATTTCATATAGCTGTCGATTTCGC |
| sequence-based reagent | NleE-N217TAG-F | This paper | PCR primers | TTTAGAACAAACTGAGCAAAATTGAAGATTAT |
| sequence-based reagent | NleE-N217TAG-R | This paper | PCR primers | GCTCAGTTTGTTCTAAATATATTTCATATAGCTGTCGATTTCG |
| sequence-based reagent | NleE-N218TAG-F | This paper | PCR primers | TTAACTAGAAACTGAGCAAAATTGAAGATTATAAAGA |
| sequence-based reagent | NleE-N218TAG-R | This paper | PCR primers | GCTCAGTTTCTAGTTAATATATTTCATATAGCTGTCGATTTCG |
| sequence-based reagent | NleE-K219TAG-F | This paper | PCR primers | TAACAACTAGCTGAGCAAAATTGAATACCCATACG |
| sequence-based reagent | NleE-K219TAG-R | This paper | PCR primers | TGCTCAGCTAGTTGTTAATATATTTCATATAGCTGTCGATT |
| sequence-based reagent | NleE-R107A-F | This paper | PCR primers | ATTAGCGGTAAAGCCCCGATGGGCCAGTTTAGCG |
| sequence-based reagent | NleE-R107A-R | This paper | PCR primers | GGGGCTTTACCGCTAATCACTGTCAGCCAGCT |
| sequence-based reagent | NleE-del I209-K214-F | This paper | PCR primers | TATATGCCGGGCGAATATATTAACAACAAACTGAGCAAAA |
| sequence-based reagent | NleE-del I209-K214-R | This paper | PCR primers | TTCGCCCGGCATATAGCTGAAAATC |
| sequence-based reagent | NleE-49AAAA52-F | This paper | PCR primers | ATTAGCGCCGCAGCCGCAGGCGTGGCAGAACTGAAACA |
| sequence-based reagent | NleE-49AAAA52-R | This paper | PCR primers | TGCGGCTGCGGCGCTAATATACTTTTTATCATAAATATTCGGG |
| sequence-based reagent | NleE-F | This paper | PCR primers | GTGCCGCGCGGCAGCCATATGATGATTAACCCGGTTACCAACACCC |
| sequence-based reagent | NleE-R | This paper | PCR primers | CTCGAGTGCGGCCGCAAGCTTTTATTTATCGTCATCATCTTTATAA |
| sequence-based reagent | NleE-HR-LF | This paper | PCR primers | GTGGAATTCCCGGGAGCCAGTGAGAGGGATAATTATCTG |
| sequence-based reagent | NleE-HR-LR | This paper | PCR primers | CTGAATCTATACCTAAAGAACGATA |
| sequence-based reagent | NleE-HR-RF | This paper | PCR primers | TAGGTATAGATTCAGAGAAGCTCAGCCACTTGTGTAGGG |
| sequence-based reagent | NleE-HR-RR | This paper | PCR primers | TAAAAAGGATCGATCCAGATATGCTCAAACGGCCAG |
| sequence-based reagent | PSMD10-F | This paper | PCR primers | TGGCCATGGAGGCCCGAATTCTGGAGGGGTGTGTGTCTAACCTAATGG |
| sequence-based reagent | PSMD10-R | This paper | PCR primers | GATCCCCGCGGCCGCGGTACCTTAACCTTCCACCATTCTCTTGAGT |
| sequence-based reagent | PSMD10-F2 | This paper | PCR primers | GGGCCCCTGGGATCCCTTATGGCCATGGAGGCCCGAATTC |
| sequence-based reagent | PSMD10-R2 | This paper | PCR primers | ATGCGGCCGCTCGAGTCTGGATCCCCGCGGCCGCGGTACC |
| sequence-based reagent | PSMD10-F3 | This paper | PCR primers | GTGCCGCGCGGCAGCCATATGGAGGGGTGTGTGTCTAACCTAATGG |
| sequence-based reagent | PSMD10-R3 | This paper | PCR primers | CTCGAGTGCGGCCGCAAGCTTTCTGGATCCCCGCGGCCGCGGTACC |
| sequence-based reagent | PSMD10-C4S-F | This paper | PCR primers | GGAGCGTGTCTAACCTAATGGTCTGCAACCTG |
| sequence-based reagent | PSMD10-C4S-R | This paper | PCR primers | TAGGTTAGACACGCTCCCCTCCAGAATTCGGGC |
| sequence-based reagent | PSMD10-C11S-F | This paper | PCR primers | AATGGTCAGCAACCTGGCCTACAGCGGGAAGC |
| sequence-based reagent | PSMD10-C11S-R | This paper | PCR primers | CCAGGTTGCTGACCATTAGGTTAGACACACACCCC |
| sequence-based reagent | PSMD10-C48S-F | This paper | PCR primers | CAAGCTCAGCTGGACATACAGAAATTGTTGAA |
| sequence-based reagent | PSMD10-C48S-R | This paper | PCR primers | ATGTCCAGCTGAGCTTGCCCAGTGCAATGCAGTT |
| sequence-based reagent | PSMD10-C107S-F | This paper | PCR primers | AAATGGCTCTACTCCCTTACATTATGCAGCTTCG |
| sequence-based reagent | PSMD10-C107S-R | This paper | PCR primers | AGGGAGTAGAGCCATTTTGATTGACAGCATTCA |
| sequence-based reagent | PSMD10-C180S-F | This paper | PCR primers | ACTTAGCCTCGGATGAGGAGAGAGTGGAAGAAGCA |
| sequence-based reagent | PSMD10-C180S-R | This paper | PCR primers | CTCATCCGAGGCTAAGTGTAGAGGAGTGTTACCC |
| sequence-based reagent | PSMD10-C4S-M1C-F | This paper | PCR primers | AATTTGCGAGGGGAGCGTGTCTAACCTAATGGTCTGCAACC |
| sequence-based reagent | PSMD10-C4S-M1C-R | This paper | PCR primers | ACGCTCCCCTCGCAAATTCGGGCCTCCATGGCC |
| sequence-based reagent | PSMD10-C4S-G3C-F | This paper | PCR primers | AATTCTGGAGTGCAGCGTGTCTAACCTAATGGTCTGCAACC |
| sequence-based reagent | PSMD10-C4S-G3C-R | This paper | PCR primers | ACGCTGCACTCCAGAATTCGGGCCTCCATGGCC |
| sequence-based reagent | PSMD10-C4S-V5C-F | This paper | PCR primers | GGAGCTGCTCTAACCTAATGGTCTGCAACCTGG |
| sequence-based reagent | PSMD10-C4S-V5C-R | This paper | PCR primers | TTAGGTTAGAGCAGCTCCCCTCCAGAATTCGGGC |
| sequence-based reagent | PSMD10-C4S-N7C-F | This paper | PCR primers | GAGCGTGTCTTGCCTAATGGTCTGCAACCTGGCC |
| sequence-based reagent | PSMD10-C4S-N7C-R | This paper | PCR primers | ATTAGGCAAGACACGCTCCCCTCCAGAATTCGGGC |
| sequence-based reagent | PSMD10-delANK1-F | This paper | PCR primers | GAATTCTGGACCAGGACAGCAGAACTGC |
| sequence-based reagent | PSMD10-delANK1-R | This paper | PCR primers | TCCTGGTCCAGAATTCGGGCCTCCATGG |
| sequence-based reagent | PSMD10-delANK2-F | This paper | PCR primers | CTAGAACTGACGATGCAGGTTGGTCTCC |
| sequence-based reagent | PSMD10-delANK2-R | This paper | PCR primers | GCATCGTCAGTTCTAGTAGCCAGGGATTTATCGG |
| sequence-based reagent | PSMD10-delANK3-F | This paper | PCR primers | ATGATAAAAATCAAAATGGCTGTACTCCCTTAC |
| sequence-based reagent | PSMD10-delANK3-R | This paper | PCR primers | TTTTGATTTTTATCATTCACTGGCACTCCAAGT |
| sequence-based reagent | PSMD10-delANK4-F | This paper | PCR primers | ATGCTGTCGACCATTATGAGGCTACAGCAATG |
| sequence-based reagent | PSMD10-delANK4-R | This paper | PCR primers | TAATGGTCGACAGCATTCACTTGAGCACCT |
| sequence-based reagent | PSMD10-delANK5-F | This paper | PCR primers | ATGCTAAGGACACTGAGGGTAACACTCCTC |
| sequence-based reagent | PSMD10-delANK5-R | This paper | PCR primers | TCAGTGTCCTTAGCATCTGGATTAGCCCCG |
| sequence-based reagent | PSMD10-delANK6-F | This paper | PCR primers | ACATCCAAAATAAAGAAGAAAAGACACCCCTGC |
| sequence-based reagent | PSMD10-delANK6-R | This paper | PCR primers | TCTTTATTTTGGATGTTTGTGGATGCTTTGT |
| sequence-based reagent | PSMD10-delANK7-F | This paper | PCR primers | ACATTGAGTAAGGTACCGCGGCCGCGG |
| sequence-based reagent | PSMD10-delANK7-R | This paper | PCR primers | GTACCTTACTCAATGTAAATACTTGCTCCTTGG |
| sequence-based reagent | PSMD10-sgRNA-F | This paper | PCR primers | CACCGTATTCTGGCCGATAAATCCC |
| sequence-based reagent | PSMD10-sgRNA-R | This paper | PCR primers | AAACGGGATTTATCGGCCAGAATAC |
| sequence-based reagent | PSMD10-HR-L-F | This paper | PCR primers | CCCACGGCATCACACCCTGCCTTTG |
| sequence-based reagent | PSMD10-HR-L-R | This paper | PCR primers | TTTATCGGCCAGAATACTCTCCTTC |
| sequence-based reagent | PSMD10-HR-R-F | This paper | PCR primers | CTACTAGAACTGACCAGGTAAAGCA |
| sequence-based reagent | PSMD10-HR-R-R | This paper | PCR primers | CTAGTGCTTACGACACTGTCTGCGA |
| sequence-based reagent | PSMD10-G3TAG-F | This paper | PCR primers | TCTGGAGTAGTGTGTGTCTAACCTAATGGTCTGCA |
| sequence-based reagent | PSMD10-G3TAG-R | This paper | PCR primers | ACACACACTACTCCAGAATTCGGGCCTCCATG |
| sequence-based reagent | PSMD10-C4TAG-F | This paper | PCR primers | GAGGGGTAGGTGTCTAACCTAATGGTCTGCAACC |
| sequence-based reagent | PSMD10-C4TAG-R | This paper | PCR primers | TTAGACACCTACCCCTCCAGAATTCGGGCCTC |
| sequence-based reagent | PSMD10-V5TAG-F | This paper | PCR primers | GGGGTGTTAGTCTAACCTAATGGTCTGCAACCTG |
| sequence-based reagent | PSMD10-V5TAG-R | This paper | PCR primers | GGTTAGACTAACACCCCTCCAGAATTCGGGCC |
| sequence-based reagent | PSMD10-N7TAG-F | This paper | PCR primers | GGTGTGTGTCTTAGCTAATGGTCTGCAACCTGGCC |
| sequence-based reagent | PSMD10-N7TAG-R | This paper | PCR primers | TAGCTAAGACACACACCCCTCCAGAATTCGGG |
| sequence-based reagent | PSMD10-M9TAG-F | This paper | PCR primers | CTAACCTATAGGTCTGCAACCTGGCCTACAGC |
| sequence-based reagent | PSMD10-M9TAG-R | This paper | PCR primers | GCAGACCTATAGGTTAGACACACACCCCTCCA |
| sequence-based reagent | PSMD10-C11TAG-F | This paper | PCR primers | AATGGTCTAGAACCTGGCCTACAGCGGGAAGC |
| sequence-based reagent | PSMD10-C11TAG-R | This paper | PCR primers | CCAGGTTCTAGACCATTAGGTTAGACACACACCCC |
| sequence-based reagent | PSMD10-K23TAG-F | This paper | PCR primers | GAAGAGTTGTAGGAGAGTATTCTGGCCGATAAATCC |
| sequence-based reagent | PSMD10-K23TAG-R | This paper | PCR primers | CTCTCCTACAACTCTTCCAGCTTCCCGCTGTA |
| sequence-based reagent | PSMD10-D37TAG-F | This paper | PCR primers | CTAGAACTTAGCAGGACAGCAGAACTGCATTGC |
| sequence-based reagent | PSMD10-D37TAG-R | This paper | PCR primers | GTCCTGCTAAGTTCTAGTAGCCAGGGATTTATCG |
| sequence-based reagent | PSMD10-W46TAG-F | This paper | PCR primers | ATTGCACTAGGCATGCTCAGCTGGACATACAG |
| sequence-based reagent | PSMD10-W46TAG-R | This paper | PCR primers | AGCATGCCTAGTGCAATGCAGTTCTGCTGTCC |
| sequence-based reagent | PSMD10-A72TAG-F | This paper | PCR primers | AGACGATTAGGGTTGGTCTCCTCTTCATATTGCG |
| sequence-based reagent | PSMD10-A72TAG-R | This paper | PCR primers | ACCAACCCTAATCGTCTTTATCATTCACTGGCAC |
| sequence-based reagent | PSMD10-K90TAG-F | This paper | PCR primers | GATTGTATAGGCCCTTCTGGGAAAAGGTGCTC |
| sequence-based reagent | PSMD10-K90TAG-R | This paper | PCR primers | GAAGGGCCTATACAATCTCATCCCGGCCAGCA |
| sequence-based reagent | PSMD10-L152TAG-F | This paper | PCR primers | CCAAGGGTAACTAGAAGATGATTCATATCCTTCTGTACTACAAA |
| sequence-based reagent | PSMD10-L152TAG-R | This paper | PCR primers | CTTCTAGTTACCCTTGGCTGCTGCCCGGTGCA |
| sequence-based reagent | PSMD10-V185TAG-F | This paper | PCR primers | GGAGAGATAGGAAGAAGCAAAACTGCTGGTGTC |
| sequence-based reagent | PSMD10-V185TAG-R | This paper | PCR primers | CTTCTTCCTATCTCTCCTCATCACAGGCTAAGTG |
| sequence-based reagent | PSMD10-E186TAG-F | This paper | PCR primers | GAGAGTGTAGGAAGCAAAACTGCTGGTGTCCC |
| sequence-based reagent | PSMD10-E186TAG-R | This paper | PCR primers | TTGCTTCCTACACTCTCTCCTCATCACAGGCTAA |
| sequence-based reagent | PSMD10-K189TAG-F | This paper | PCR primers | AGAAGCATAGCTGCTGGTGTCCCAAGGAGCAA |
| sequence-based reagent | PSMD10-K189TAG-R | This paper | PCR primers | CCAGCAGCTATGCTTCTTCCACTCTCTCCTCATC |
| sequence-based reagent | PSMD10-L190TAG-F | This paper | PCR primers | GCAAAATAGCTGGTGTCCCAAGGAGCAAGTAT |
| sequence-based reagent | PSMD10-L190TAG-R | This paper | PCR primers | GACACCAGCTATTTTGCTTCTTCCACTCTCTCCTC |
| sequence-based reagent | PSMD10-S193TAG-F | This paper | PCR primers | TGCTGGTGTAGCAAGGAGCAAGTATTTACATTGAGAAT |
| sequence-based reagent | PSMD10-S193TAG-R | This paper | PCR primers | TCCTTGCTACACCAGCAGTTTTGCTTCTTCCA |
| sequence-based reagent | PSMD10-Q194TAG-F | This paper | PCR primers | TGTCCTAGGGAGCAAGTATTTACATTGAGAATAAAGA |
| sequence-based reagent | PSMD10-Q194TAG-R | This paper | PCR primers | ACTTGCTCCCTAGGACACCAGCAGTTTTGCTTCT |
| sequence-based reagent | PSMD10-G195TAG-F | This paper | PCR primers | GTCCCAATAGGCAAGTATTTACATTGAGAATAAAGAAGAA |
| sequence-based reagent | PSMD10-G195TAG-R | This paper | PCR primers | TACTTGCCTATTGGGACACCAGCAGTTTTGCT |
| sequence-based reagent | PSMD10-A196TAG-F | This paper | PCR primers | GTCCCAAGGATAGAGTATTTACATTGAGAATAAAGAAGAAAAGAC |
| sequence-based reagent | PSMD10-A196TAG-R | This paper | PCR primers | TACTCTATCCTTGGGACACCAGCAGTTTTGCT |
| sequence-based reagent | PSMD10-S197TAG-F | This paper | PCR primers | CCAAGGAGCAAGTTAGATTTACATTGAGAATAAAGAAGAAAAGACA |
| sequence-based reagent | PSMD10-S197TAG-R | This paper | PCR primers | TCTAACTTGCTCCTTGGGACACCAGCAGTTTT |
| sequence-based reagent | PSMD10-E204TAG-F | This paper | PCR primers | ATAGGAAAAGACACCCCTGCAAGTGGCCAAAG |
| sequence-based reagent | PSMD10-E204TAG-R | This paper | PCR primers | GGGGTGTCTTTTCCTATTTATTCTCAATGTAAATACTTGCTCCTT |
| sequence-based reagent | PSMD10-I219TAG-F | This paper | PCR primers | CCTGGGTTTATAGCTCAAGAGAATGGTGGAAGGTTAAG |
| sequence-based reagent | PSMD10-I219TAG-R | This paper | PCR primers | TGAGCTATAAACCCAGGCCACCTTTGGCCACT |
| sequence-based reagent | ATG7-F | This paper | PCR primers | ATGGCCATGGAGGCCGCGGCAGCTACGGGGGATCCTGGAC |
| sequence-based reagent | ATG7-R | This paper | PCR primers | GATCCCCGCGGCCGCTCAGATGGTCTCATCATCGCTCATG |
| sequence-based reagent | ATG7-NTD-F | This paper | PCR primers | AGGATGGTGAACCTCTGAGCGGCCGCGGGGATCCAGACAT |
| sequence-based reagent | ATG7-NTD-R | This paper | PCR primers | GAGGTTCACCATCCTTGGTCCCATG |
| sequence-based reagent | ATG7-CTD-F | This paper | PCR primers | ATGGCCATGGAGGCCCCTAAAAGGTTAGCTGAGTCATCAG |
| sequence-based reagent | ATG7-CTD-R | This paper | PCR primers | GGCCTCCATGGCCATAAGTTTATCG |
| sequence-based reagent | ATG7-AD-F | This paper | PCR primers | ATGGCCATGGAGGCCCCTAAAAGGTTAGCTGAGTCATCAG |
| sequence-based reagent | ATG7-AD-R | This paper | PCR primers | GATCCCCGCGGCCGCTCAACAAGCTGTACATTTGTCAAATGCC |
| sequence-based reagent | ATG7-del ECTD-F | This paper | PCR primers | AAATGTACAGCTTGTTGAGCGGCCGCGGGGATCCAGACAT |
| sequence-based reagent | ATG7-del ECTD-R | This paper | PCR primers | ACAAGCTGTACATTTGTCAAATGCC |
| sequence-based reagent | ATG7-del C terminal-F | This paper | PCR primers | TCCTTCTTAGAAGACTGAGCGGCCGCGGGGATCCAGACAT |
| sequence-based reagent | ATG7-del C terminal-R | This paper | PCR primers | GTCTTCTAAGAAGGAATGTGAAGAA |
| sequence-based reagent | ATG7-QPCR-F | This paper | PCR primers | TGCTATCCTGCCCTCTGTCTT |
| sequence-based reagent | ATG7-QPCR-R | This paper | PCR primers | TGCCTCCTTTCTGGTTCTTTT |
| sequence-based reagent | β-Actin-QPCR-F | This paper | PCR primers | CCCAAGGCCAACCGCGAGAAGATG |
| sequence-based reagent | β-Actin-QPCR-R | This paper | PCR primers | GTCCCGGCCAGCCAGGTCCAGA |
| sequence-based reagent | ATG3-F | This paper | PCR primers | ATGGCCATGGAGGCCATGCAGAATGTGATTAATACTGTGA |
| sequence-based reagent | ATG3-R | This paper | PCR primers | GATCCCCGCGGCCGCTTACATTGTGAAGTGTCTTGTGTAG |
| sequence-based reagent | LC3B-F | This paper | PCR primers | ATGGCCATGGAGGCCATGCCGTCGGAGAAGACCTTCAAGC |
| sequence-based reagent | LC3B-R | This paper | PCR primers | GATCCCCGCGGCCGCTTACACTGACAATTTCATCCCGAAC |
| sequence-based reagent | ATG10-F | This paper | PCR primers | ATGGCCATGGAGGCCATGGAAGAAGATGAGTTCATTGGAG |
| sequence-based reagent | ATG10-R | This paper | PCR primers | GATCCCCGCGGCCGCTTAAGGGACATTTCGTTCATCCTGA |
| sequence-based reagent | ATG12-F | This paper | PCR primers | ATGGCCATGGAGGCCATGGCGGAGGAGCCGCAGTCTGTGT |
| sequence-based reagent | ATG12-R | This paper | PCR primers | GATCCCCGCGGCCGCTCATCCCCACGCCTGAGACTTGCAG |
| sequence-based reagent | Rpt3-F | This paper | PCR primers | ATGGCCATGGAGGCCGAGGAGATAGGCATCTTGGTG |
| sequence-based reagent | Rpt3-R | This paper | PCR primers | GATCCCCGCGGCCGCTCACTTGTAAAACTCATGCTCCTGC |
| sequence-based reagent | TAB2-NZF-F | This paper | PCR primers | GGGCCCCTGGGATCCGGAGCTCAGTGGAATTGTACCGCCT |
| sequence-based reagent | TAB2-NZF-R | This paper | PCR primers | TCAGAAATGCCTTGGTCAGAAATGCCTTGGCATCTCACAC |
| sequence-based reagent | HSF1-F | This paper | PCR primers | ATGGCCATGGAGGCCATGGATCTGCCCGTGGGCCCCGGCG |
| sequence-based reagent | HSF1-R | This paper | PCR primers | GATCCCCGCGGCCGCCTAGGAGACAGTGGGGTCCTTGGCT |
| sequence-based reagent | Rpt3-F2 | This paper | PCR primers | GGGCCCCTGGGATCCGAGGAGATAGGCATCTTGGTG |
| sequence-based reagent | Rpt3-R2 | This paper | PCR primers | CGCTCGAGTCGACCCTCACTTGTAAAACTCATGCTCCTGC |
